# Chromosomal copy number variation analysis by next generation sequencing confirms ploidy stability in *Trypanosoma brucei* subspecies

**DOI:** 10.1099/mgen.0.000223

**Published:** 2018-09-27

**Authors:** Laila Viana Almeida, Anderson Coqueiro-dos-Santos, Gabriela F. Rodriguez-Luiz, Richard McCulloch, Daniella Castanheira Bartholomeu, João Luís Reis-Cunha

**Affiliations:** ^1^​ Departamento de Parasitologia, Universidade Federal de Minas Gerais – Instituto de Ciências Biológicas, Belo Horizonte, Brazil; ^2^​ University of Glasgow, Wellcome Centre for Molecular Parasitology, Glasgow, UK; ^3^​ Universidade Federal de Minas Gerais, Belo Horizonte, Brazil

**Keywords:** *Trypanosoma brucei*, aneuploidy, genome, chromosomal copy number variation, genome replication

## Abstract

Although aneuploidy usually results in severe abnormalities in multicellular eukaryotes, recent data suggest that it could be beneficial for unicellular eukaryotes, such as yeast and trypanosomatid parasites, providing increased survival under stressful conditions. Among characterized trypanosomatids, *Trypanosoma cruzi*, *Trypanosoma brucei* and species from the genus *Leishmania* stand out due to their importance in public health, infecting around 20 million people worldwide. The presence of aneuploidies in *T. cruzi* and *Leishmania* was recently confirmed by analysis based on next generation sequencing (NGS) and fluorescence *in situ* hybridization, where they have been associated with adaptation during transmission between their insect vectors and mammalian hosts and in promoting drug resistance. Although chromosomal copy number variations (CCNVs) are present in the aforementioned species, PFGE and fluorescence cytophotometry analyses suggest that aneuploidies are absent from *T. brucei*. A re-evaluation of CCNV in *T. b gambiense* based on NGS reads confirmed the absence of aneuploidies in this subspecies. However, the presence of aneuploidies in the other two *T. brucei* subspecies, *T. b. brucei* and *T. b. rhodesiense*, has not been evaluated using NGS approaches. In the present work, we tested for aneuploidies in 26 *T. brucei* isolates, including samples from the three *T. brucei* subspecies, by both allele frequency and read depth coverage analyses. These analyses showed that none of the *T. brucei* subspecies presents aneuploidies, which could be related to differences in the mechanisms of DNA replication and recombination in these parasites when compared with *Leishmania*.

## Data Summary

All read libraries used in this work are listed and characterized in Table S1. All were downloaded from NCBI Sequence Read Archive.

**Impact Statement**

Aneuploidy, the gain or loss of copies of chromosomes, is usually detrimental. For instance, in humans, an extra copy of chromosome 21 results in Down syndrome, a number of conditions arise from imbalanced numbers of sexual chromosomes, and aneuploidies are associated with many types of tumours. On the other hand, some single-celled microbes, such as yeast and trypanosomatid parasites, the latter an important group of pathogens that infect around 20 million people worldwide, appear to rely on aneuploidy as a mechanism to allow adaptation to changing environments, such as different hosts and drug exposure. Surprisingly, among characterized trypanosomatid parasites, some species present aneuploidies, while others do not. Therefore, comparing the presence of aneuploidy in members of the trypanosomatid family with processes associated with genomic stability could reveal cellular mechanisms that lead to aneuploidy and allow it to be tolerated. Understanding the variations in ploidy in these organisms could provide insights into important processes that affect the infectivity of parasites (and other pathogens) and contribute to better knowledge of the cellular processes that dictate the stability and propagation of genomes in all cells.

## Introduction

*Trypanosoma brucei*, a protozoan parasite from the family Trypanosomatidae, is the causative agent of sleeping sickness, an endemic disease in 36 sub-Saharan African countries [1]. Other members of this family include parasites of medical relevance such as *Trypanosoma cruzi* and *Leishmania*, the aetiological agents of Chagas disease and leishmaniasis, respectively. *T. brucei* is subdivided into three subspecies, *T. brucei gambiense*, *T. brucei rhodesiense* and *T. brucei brucei. T. b. gambiense* is responsible for ~90 % of reported human cases of infection and is mainly found in central and western Africa, whereas *T. b. rhodesiense* is primarily found in eastern and southern Africa [1, 2]. *T. b. brucei* is usually restricted to non-human animal infections, although human cases have been reported [3].

As cytogenetic analyses are hampered in trypanosomatids by the lack of chromosome condensation during mitosis, karyotype studies in these parasites were initially evaluated by PFGE and fluorescence cytophotometry [4–8]. The development of next generation sequencing (NGS) methodologies and the availability of chromosomal-level assemblies of protozoans from the family Trypanosomatidae [9–12] have enabled a re-evaluation of chromosomal copy number variation (CCNV) occurrence based on read depth coverage (RDC) and allele frequency [13–16]. This new methodology was able to confirm the presence of mosaic aneuploidy in *Leishmania*, explaining the non-stoichiometric staining intensities of different chromosomal bands observed in PFGE analysis [4]. CCNV was observed in several species of the genus *Leishmania*, where the pattern of aneuploidies varies among species and even within a population [13, 14, 17–19]. Aneuploidies were shown to greatly impact the phenotype of the parasite, altering gene expression levels, promoting drug resistance and influencing host interchange adaptations [20–22]. Therefore, while aneuploidy is usually lethal or results in severe abnormalities in multicellular eukaryotes [23–25], it could provide rapid adaptation to stressful conditions in unicellular eukaryotes. Chromosomal gain/loss events were also observed among and within *T. cruzi* discrete typing units (DTUs) [15, 26], suggesting that aneuploidies are a common event in trypanosomatids.

In contrast to what has been observed for the genus *Leishmania* and *T. cruzi* DTUs, PFGE and fluorescence cytophotometry analyses suggest that *T. brucei* is mainly diploid [5, 6, 8, 27–29]. Although triploid *T. brucei* parasites have been observed following experimental crossing [30, 31], no triploid *T. brucei* isolates have been previously reported from the field. From the three subspecies, only *T. b. gambiense* ploidy has been evaluated based on whole genome sequencing and RDC, and no aneuploidies were detected [32]. However, the occurrence of aneuploidies in *T. b. brucei* and *T. b. rhodesiense* has not been evaluated using NGS approaches. Therefore, the objective of this work was to estimate CCNV in all three *T. brucei* subspecies using RDC analysis, compare this with what has already been reported for other trypanosomatids and correlate the pattern of aneuploidy with cellular processes associated with genomic stability.

## Methods

### Read libraries and processing

A total of 27 *T. brucei* whole genome read libraries were downloaded from the NCBI Sequence Read Archive (SRA). These included four samples from *T. b. brucei*, two from *T. b. gambiense* and 21 from *T. b. rhodesiense* (Table S1, available in the online version of this article). The reads from each library were submitted to a quality check using the FASTQc tool (http://www.bioinformatics.babraham.ac.uk/projects/fastqc/). Read libraries were filtered using Trimmomatic [33], with a minimum threshold of phred quality 20 and a minimal length of 40 nucleotides.

### Read mapping

The 27 quality trimmed read libraries were mapped on the 11 chromosomal-size *T. b. brucei* 927 version 28 scaffolds (TritrypDB, http://tritrypdb.org/tritrypdb/), using the BWA-mem software [34, 35]. The mapped reads were trimmed based on a mapping quality of 30 using SAMtools v1.1 [36]. The percentage of mapped reads was estimated with SAMtools flagstat, where only libraries in which more than 70 % of the reads mapped were used (Table S1).

### Chromosomal copy number variation estimations

Two methodologies were used to evaluate the occurrence of CCNV in *T. brucei*: allele frequency of heterozygous positions and RDC analysis.

To evaluate CCNV based on allele frequency, we performed SNP calling using GATK v3.3 [37, 38] in each read library mapped to the *T. b. brucei* 927 reference genome. Initially, duplicated reads were marked using Picard v1.119 (https://github.com/broadinstitute/picard). Illumina reads were then re-aligned with GATK RealignerTargetCreator and SNP records were obtained with GATK HaplotypeCaller, with minimal confidence threshold for calling of 30 (Phred scale) and minimum threshold confidence for emission of 10 (Phred scale). Next, GATK SelectVariants was used to report only SNPs, excluding insertion/deletions, and VCFfilter was used to select SNP positions with read depth of at least 10 and quality higher than 10. Next, the heterozygous SNP positions in all genes, excluding variant surface glycoproteins (VSGs) and expression site associated genes (ESAGs), were retrieved using in-house Perl scripts, where only SNPs with a support of at least five reads in each variant allele were reported. For each chromosome, the proportion of read depth in alleles in each variant of a predicted heterozygous site was obtained and rounded to the second decimal place, ranging from 0.01 to 1.00, and an approximate distribution of base frequencies for each chromosome was obtained based on Perl scripts and plotted in R (www.r-project.org, R Development 2010). To estimate the overall ploidy of each genome, the same methodology was applied, but the heterozygous positions of all coding sequences (CDS) from all chromosomes were computed simultaneously. Disomic chromosomes are expected to have a peak of 0.50, while trisomic chromosomes are expected to have peaks of 0.33 and 0.66, and tetrasomic chromosomes a combination of 0.25, 0.50 and 0.75 peaks.

The estimation of ploidy variation based on RDC assumes that if the median RDC of a chromosome is higher or lower than the median RDC of the whole genome, this could represent chromosomal gains or losses, respectively. To that end, the chromosomal copy numbers were estimated by the median of *d*_c_/*d*_g_ in a chromosome using in-house Perl scripts and BEDtools, where *d*_c_ represents the median RDC of all genes in a given chromosome, excluding the multigene VSG and ESAG families, and *d*_g_ corresponds to the median genome coverage. Initially, the median RDC of all genes, excluding VSGs and ESAGs, for each chromosome was obtained. Next, genes with a coverage lower than 50 % of the gene length were excluded. Then, genes with outlier coverage for each chromosome were excluded, based on an iterative Grubb’s test, with *P*<0.05. Finally, the median and quantile coverages of each chromosome were plotted in boxplots using R. Median values close to 1 confirm that the chromosomal somy is close to the genome ploidy. This methodology is similar to the one used by Downing *et al.* [14] for *Leishmania*, Reis-Cunha *et al.* [15] for *T. cruzi* and Tihon *et al.* for *Trypanosoma congolense* [39]. Genome coverage was estimated based on the median RDC of all genes in the genome, excluding VSGs and ESAGs, using Perl scripts.

### Principal component analysis (PCA) and maximum-likelihood phylogeny of the *T. brucei* SNPs

To estimate the distance among the 26 *T. brucei* samples based on whole genome differential SNPs, a consensus nuclear genomic sequence was generated for each sample, using GATK FastaAlternateReferenceMaker (https://software.broadinstitute.org/gatk/documentation/tooldocs/current/org_broadinstitute_gatk_tools_walkers_fasta_FastaAlternateReferenceMaker.php). This genomic sequence was used as input to generate PCA plots and maximum-likelihood phylogeny estimations.

To generate the PCA plot, a distance matrix based on differential SNPs was generated and loaded in the R caret package (http://topepo.github.io/caret/index.html). To evaluate the maximum-likelihood phylogeny, the best-fitting nucleotide substitution model for the phylogenetic analysis was determined using Jmodeltest [40]. The maximum-likelihood phylogenetic tree was built using PhyML [41], with the Generalized Time Reversible model with 1000 bootstrap replicates, proportion of invariable sites of 0.96 and gamma distribution of 0.56. The final phylogenetic tree images were built using FigTree v.1.4.2 software (http://tree.bio.ed.ac.uk/software/figtree/).

## Results and Discussion

### Chromosomal copy number evaluations

**Fig. 1.**
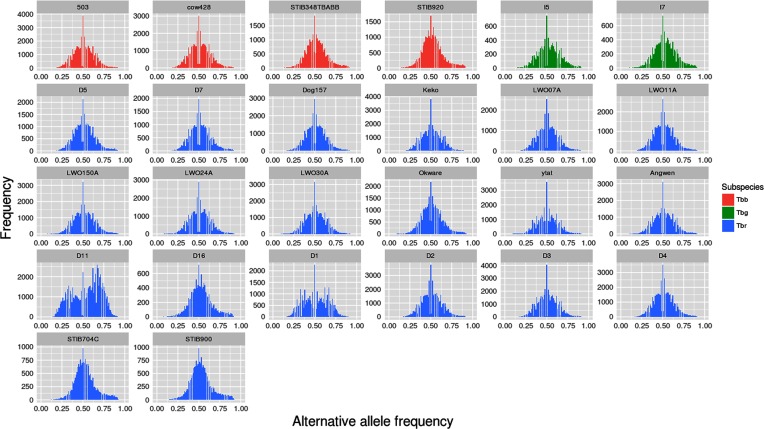
*T. brucei* subspecies whole genome allele frequency ratio. In each image, the *x*-axis denotes the allele frequency ratio of heterozygous positions from 0 (0 %) to 1 (100 %), while the *y*-axis denotes the occurrence of that allele frequency in the genome. An allele frequency ratio peak of 0.5 denotes that the majority of heterozygous positions in the genome had a similar depth of coverage for both alleles, suggesting diploidy. Peaks of 0.33 and/or 0.66 suggest triploidy, and peaks of 0.25, 0.5 and 0.75 suggest tetraploidy. *T. b. brucei* samples are in red, *T. b. gambiense* in green and *T. b. rhodesiense* in blue.

From the 27 *T. brucei* read libraries evaluated in this work, 26 presented with more than 70 % of the trimmed reads mapping to the 11 *T. b. brucei* 927 chromosomal-size scaffolds and were used in the parasite ploidy estimations (Table S1). Initially, the overall genome ploidy of each *T. brucei* strain/isolate was estimated based on the allele frequency of heterozygous positions in all non-VSG and non-ESAG genes, comprising 9295 genes (Fig. 1, Table S2). Based on this analysis, 25 samples presented a single mode of 0.5, suggesting that they are mainly diploid, while *T. b. rhodesiense* isolate D11 had modes of ~0.33, ~0.5 and ~0.66, suggesting mixed disomic/trisomic chromosomes, or that isolate D11 corresponds to a mixed infection, with more than one parasite population in the same isolate (Fig. 1). This isolate clustered together with other *T. b. rhodesiense* isolates in a PCA based on SNP data (Fig. S1). Although mainly diploid, *T. brucei* isolate D1 also presented discrete peaks of ~0.33 and ~0.66, suggesting that a part of its population could also be triploid. The absence of any strong evidence for triploidy in D1 could be due to the lower genome coverage when compared to D11 (Table S1 and Fig. S2).

**Fig. 2.**
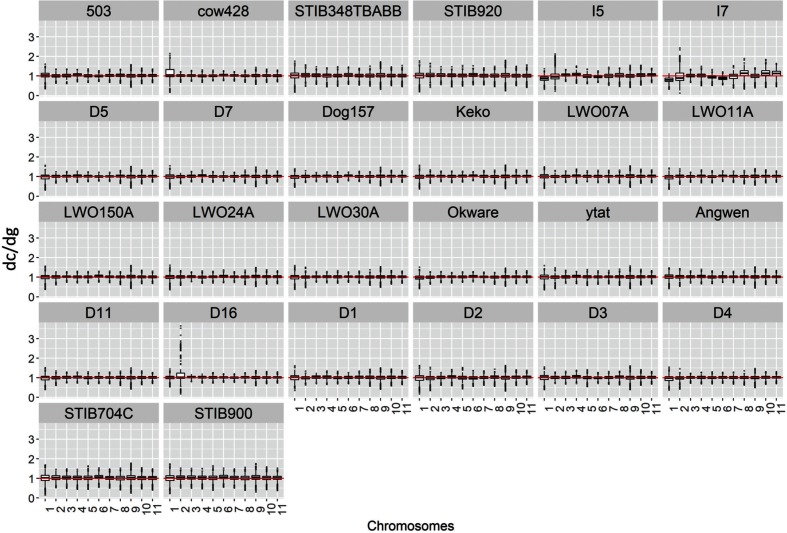
*T. brucei* subspecies chromosome somy estimated based on *d*_c_/*d*_g_. In each image, the *y*-axis corresponds to a boxplot of the median coverage of all genes in a chromosome normalized by genome coverage, where the median value corresponds to the chromosome-predicted somy. Each bar on the *x*-axis represents a *T. brucei* chromosome, numbered from 1 to 11. A median *d*_c_/*d*_g_ value of ~1 means that the chromosomally estimated copy number was similar to the genome ploidy.

**Fig. 3.**
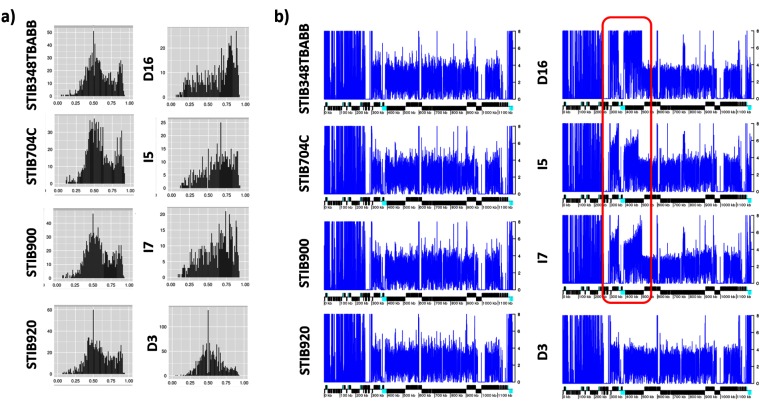
*T. brucei* chromosome 2 allele frequency ratio and segmental duplication. (a) Allele frequency ratio distribution for chromosome 2. The *x*-axis denotes the allele frequency ratio of heterozygous positions from 0 (0 %) to 1 (100 %), while the *y*-axis denotes the occurrence of that allele frequency in chromosome 2. (b) In this image, the blue line corresponds to the normalized RDC of each position in a chromosome, estimated by the ratio between the RDC and the genome coverage. Below, the protein-coding genes are depicted as rectangles drawn proportional to their length, and their coding strand is indicated by their position above (top strand) or below (bottom strand) the central line. Cyan boxes represent VSGs and ESAGs. Black boxes represent all other genes. Segmental duplications not located in sub-telomeric regions are highlighted by a red box. Isolates D16, I5 and I7 show a major skewed allele frequency distribution, while STIB348TBABB, STIB704C, STIB900 and STIB920 show a minor skewed allele frequency distribution. Isolate D3 was added as an example of a disomic chromosome.

An evaluation of the somy of each chromosome from each isolate by RDC revealed that the chromosomal somy is in accordance with the whole genome ploidy, where the median RDC of each chromosome was similar to the median genome coverage, even for isolate D11 (Fig. 2, and Table S3). Similar results were obtained based on chromosomal somy estimations by allele frequency (Fig. S3). The only exceptions were for chromosome 2 from isolates D16, I5 and I7, which showed a major skewed allele frequency ratio, and isolates STIB348TBABB, STIB704C, STIB900 and STIB920, which presented a minor skewed allele frequency ratio (Fig. 3a). The evaluation of the RDC variations alongside each chromosome (Fig. S4) revealed a large segmental duplication in chromosome 2 from isolates D16, I5 and I7, which was not observed in the other *T. brucei* isolates (Fig. 3b). Interestingly, I5 and I7 are *T. b. gambiense* isolates, while D16 was previously classified as a *T. b. rhodesiense* isolate [42]. In our PCA evaluations (Fig. S1) as well as maximum-likelihood phylogenetic analysis (Fig. S5), isolate D16 clustered together with I5 and I7, suggesting that it could actually be a *T. b. gambiense* isolate. Alternatively, D16 could actually be a *T. b. rhodesiense* isolate and the three *T. brucei* subspecies may not be monophyletic as previously suggested [42].

These combined results confirm an overall absence of aneuploidies in all three *T. brucei* subspecies, as previously observed by RDC analysis in *T. b. gambiense* [32] and by PFGE and fluorescence cytophotometry analyses for the three subspecies [6, 27]. The only isolate with a non-diploid pattern in our analysis, *T. b. rhodesiense* D11, presents all its chromosomes in the same polysomic state (Fig. S3). Triploid *T. brucei* parasites have already been obtained in experimental mating in the tsetse fly, suggesting that *T. brucei* can sustain whole genome aneuploidies in laboratory conditions [30, 31]. However, polyploidy has not yet been documented in *T. brucei* field isolates [43]. Recent ploidy estimations in 56 *Trypanosoma congolense* field isolates led to the identification of one triploid lineage, BANANCL2 [39]. This triploid isolate was viable and stable during all the life-cycle stages of the parasite and was efficiently transmitted to mice, resulting in systemic infections [43]. These results suggest that parasites from the Salivarian evolutionary branch, *T. brucei* and *T. congolense*, can sustain polysomy, but do not appear to be aneuploid as observed in *T. cruzi* [15, 26] and in *Leishmania* [13], where different chromosomes present different somies. CCNV evaluations in other parasites from the Salivaria clade, such as *Trypanosoma vivax* and *Trypanosoma evansi*, as well as from protozoans closely related to *T. cruzi* (e.g. *Trypanosoma rangeli*, *Trypanosoma grayi*) and *Leishmania* (e.g. *Crithidia fasciculata*, *Leptomonas pyrrhicoris*) would be valuable to understand this potential dichotomy in genome structure in kinetoplastids.

**Fig. 4.**
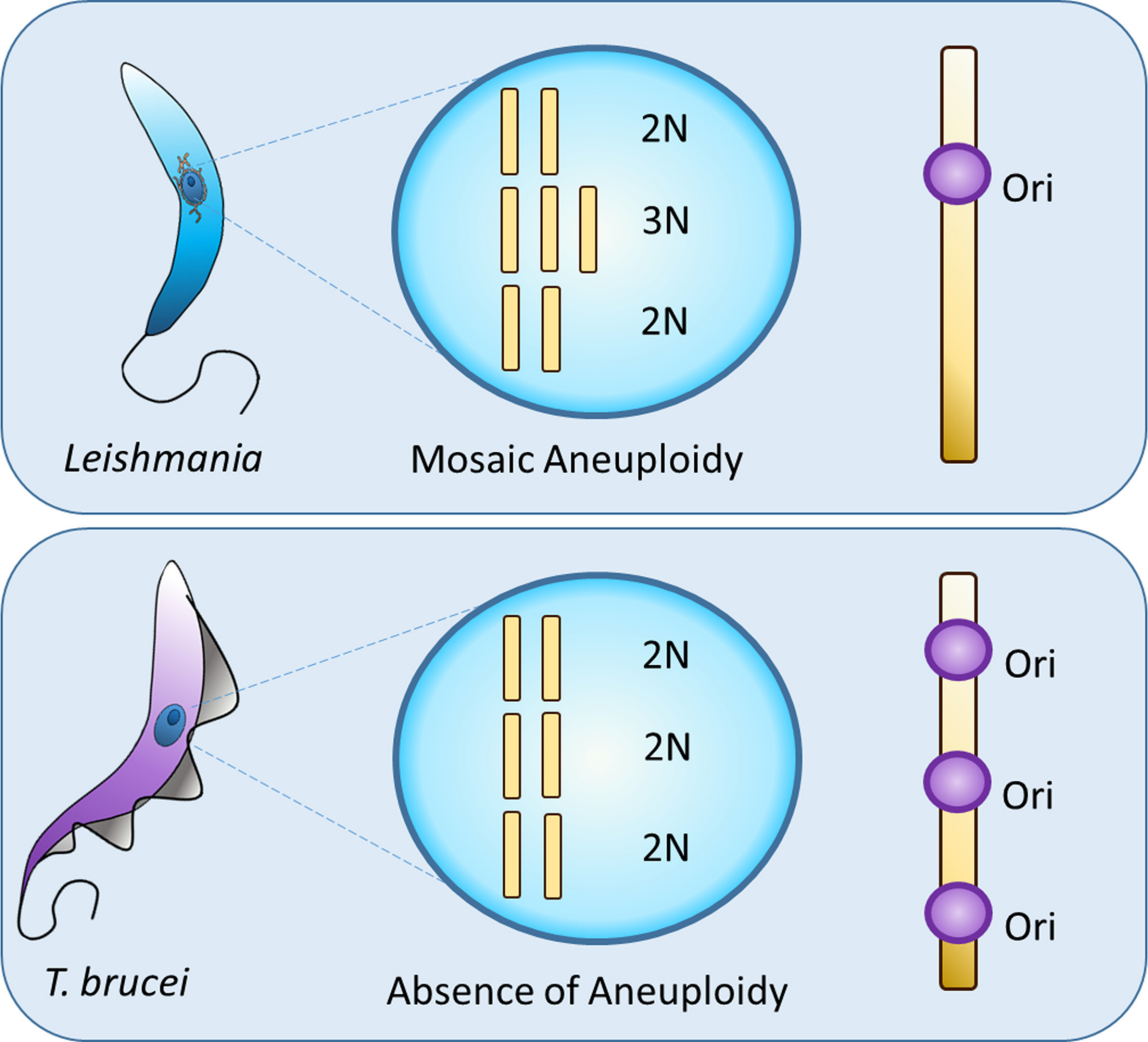
Schematic representation of aneuploidy in trypanosomatids. Parasites from the genus *Leishmania* that present mosaic aneuploidy have a preferential origin of DNA replication (Ori) in each chromosome, while all three *T. brucei* subspecies lack aneuploidies and present several origins of replication in each chromosome.

Transcription in trypanosomatids is polycistronic, sharing a profound overlap with DNA replication, where both processes frequently start at strand-switch regions [44, 45]. However, whereas each of the 11 megabase-sized *T. brucei* chromosomes have several origins of DNA replication [44], the same mapping strategy in *Leishmania* revealed either a single or a highly preferred origin of replication per chromosome [46]. It is possible that this differing pattern of DNA replication initiation accounts for the distinct ploidies of the two related parasites. For instance, a clash between the transcription and replication machineries at the preferential or singular origin in *Leishmania* may result in a duplication failure of a given chromosome, which could compromise survival of the parasite. For this reason, the retention of extra chromosomal copies in *Leishmania* could mitigate against eventual chromosomal loss (Fig. 4). In fact, it has already been suggested that CCNV in *Leishmania* is generated by asymmetric chromosomal replications, yielding chromosome gains and losses after a number of mitotic generations [47, 48]. The presence of several origins of replication in each *T. brucei* chromosome would shield this parasite from chromosomal losses due to clashes between the replication and transcription machineries, as DNA replication that emanates from one origin but suffers a blockade could still be completed using replication from another origin [44]. Alternatively, aneuploidies could be generated by recombination events, as observed in the *Candida albicans* parasexual cycle [49]. In this model, the fusion of parental cells is followed by karyogamy, resulting in a polyploid progeny that undergoes reductional mitotic divisions and genome erosion [49]. Environmental pressures could then select parasite cells that present extra copies of chromosomes whose amplification could be advantageous for parasite survival due to increased copy number of their genes. This mechanism is supported by the subtetraploidy found in *T. cruzi* experimental hybrids, which present 70 % higher DNA content compared to parental strains [50, 51], and by chromosomal amplification variations as *Leishmania* migrates from the insect vector to the mammalian host [20].

### Conclusion

The putative presence of aneuploidies in *Leishmania* and *T. cruzi* but not in *T. brucei* or *T. congolense* suggests that although evolutionarily related, these parasites present different tolerance to CCNVs. Whether Salivarian parasites lost an ancestral ability to sustain aneuploidy, or whether *Leishmania* and *T. cruzi* evolved this mechanism independently is still unknown. The evaluation of CCNV in a larger number of trypanosomatids and kinetoplastids, and correlating this property with DNA replication initiation and recombination, will shed light on the biological mechanisms behind aneuploidy generation in this evolutionary grouping, which will have relevance for all eukaryotes.

## Supplementary Data

Supplementary File 1

Supplementary File 2
