# Supplementary material for: Chromosomal copy number variation analysis by next generation sequencing confirms ploidy stability in Trypanosoma brucei subspecies
Source: Microb Genom. 2018 Sep 27;4(10):e000223. doi: 10.1099/mgen.0.000223 (PMC6249438; doi:10.1099/mgen.0.000223)

Supplementary Figure 1: PCA of *T. brucei* isolates' genomic distance based on differential SNPs. The genomic distance estimated by three dimension principal component analysis of the 26 *T. brucei* isolates is shown in different angles. This three dimensional representation comprises 47.2% of the variability observed in the evaluated *T. brucei* Isolates. *T. b. rhodesiense* isolates are represented in blue, while *T. b. brucei* are in red, and *T. b. gambiense* in green. The black circle correspond to the *T. b. brucei* 927 reference genome, used as template in the SNP calling. The red arrow points to the D11 isolate, the only potentially trisomic *T. brucei* isolate evaluated.

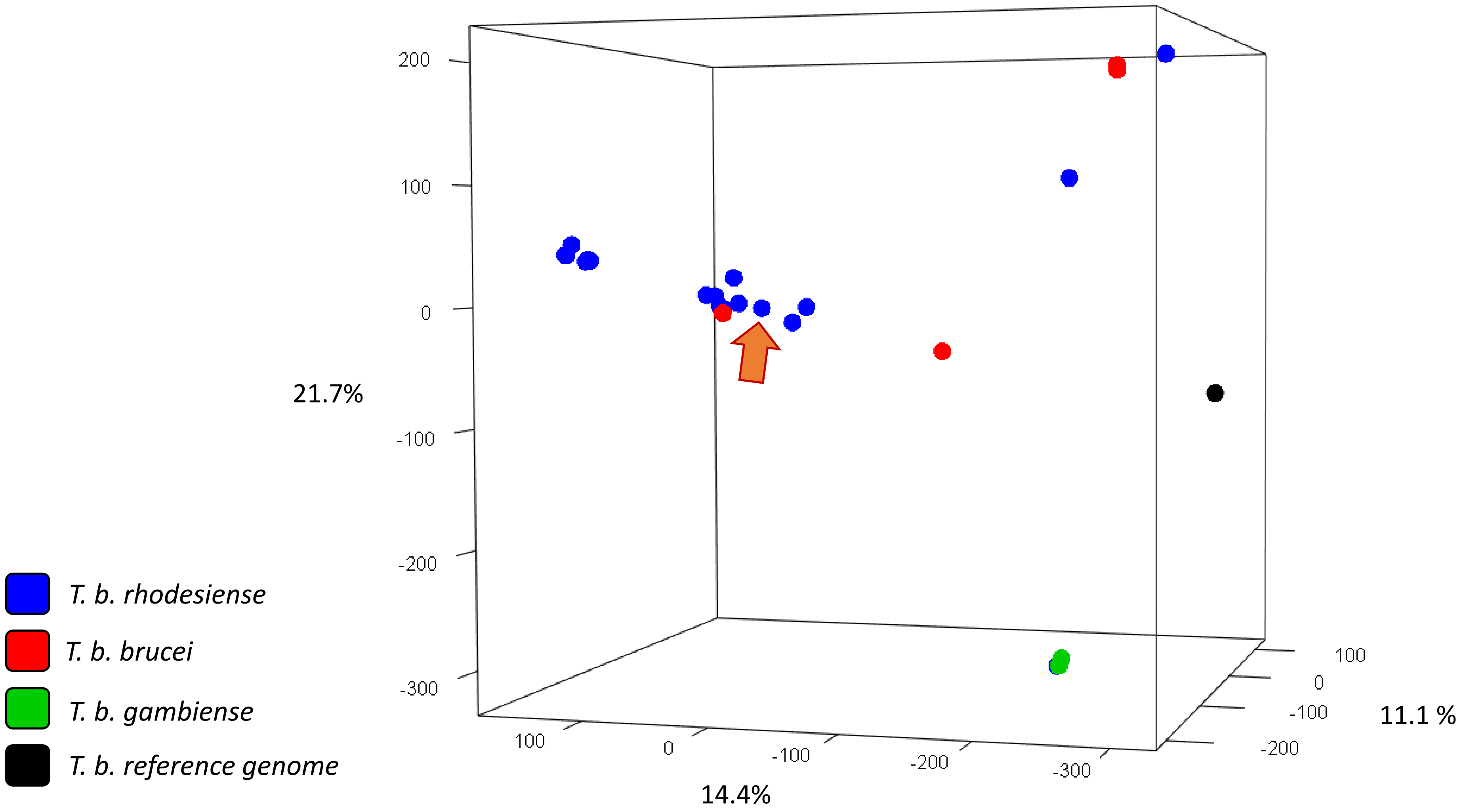

Supplementary Figure 1: PCA of *T. brucei* isolates' genomic distance based on differential SNPs. The genomic distance estimated by three dimension principal component analysis of the 26 *T. brucei* isolates is shown in different angles. This three dimensional representation comprises 47.2% of the variability observed in the evaluated *T. brucei* Isolates. *T. b. rhodesiense* isolates are represented in blue, while *T. b. brucei* are in red, and *T. b. gambiense* in green. The black circle correspond to the *T. b. brucei* 927 reference genome, used as template in the SNP calling. The red arrow points to the D11 isolate, the only potentially trisomic *T. brucei* isolate evaluated.

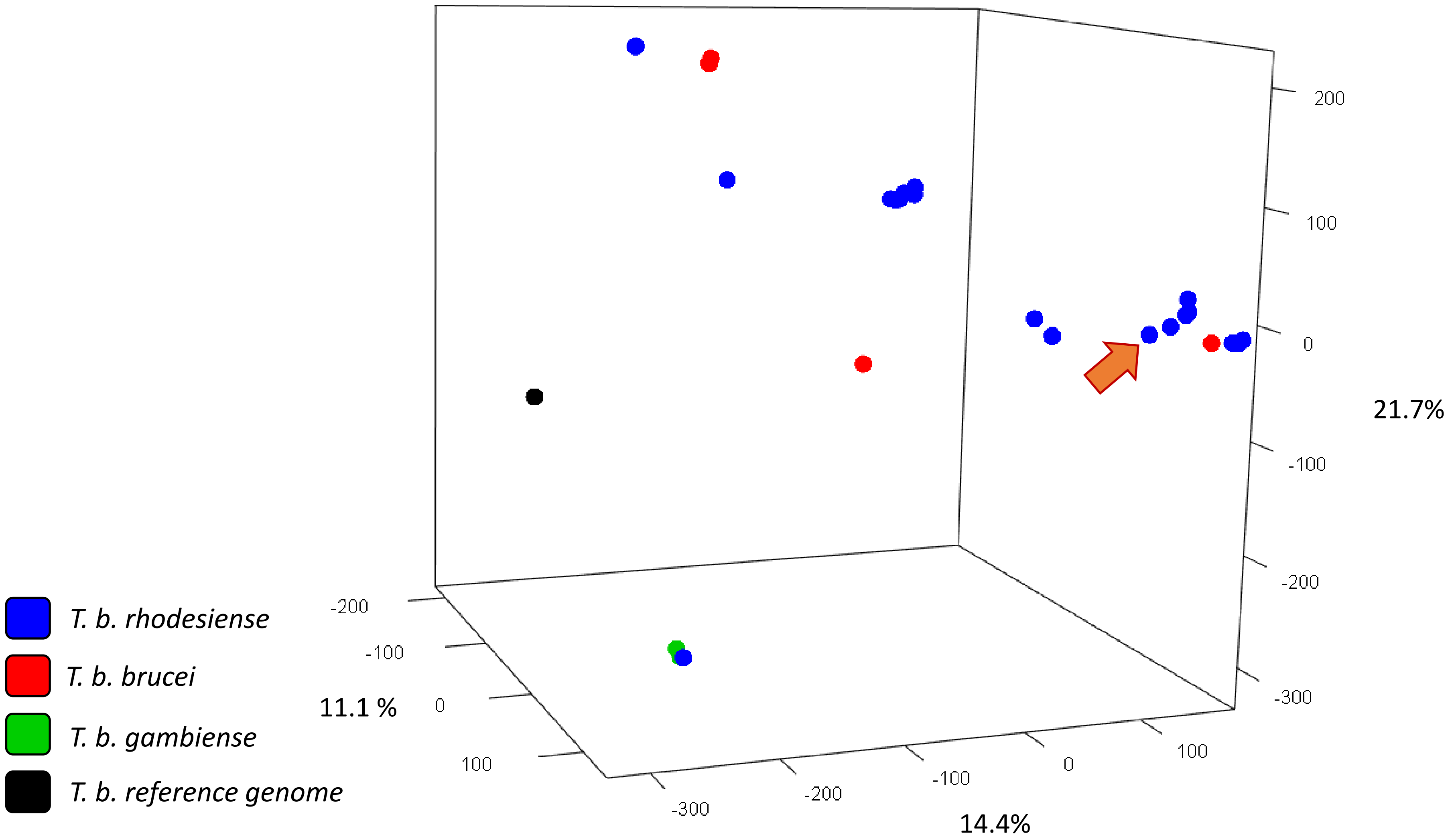

Supplementary Figure 1: PCA of *T. brucei* isolates' genomic distance based on differential SNPs. The genomic distance estimated by three dimension principal component analysis of the 26 *T. brucei* isolates is shown in different angles. This three dimensional representation comprises 47.2% of the variability observed in the evaluated *T. brucei* Isolates. *T. b. rhodesiense* isolates are represented in blue, while *T. b. brucei* are in red, and *T. b. gambiense* in green. The black circle correspond to the *T. b. brucei* 927 reference genome, used as template in the SNP calling. The red arrow points to the D11 isolate, the only potentially trisomic *T. brucei* isolate evaluated.

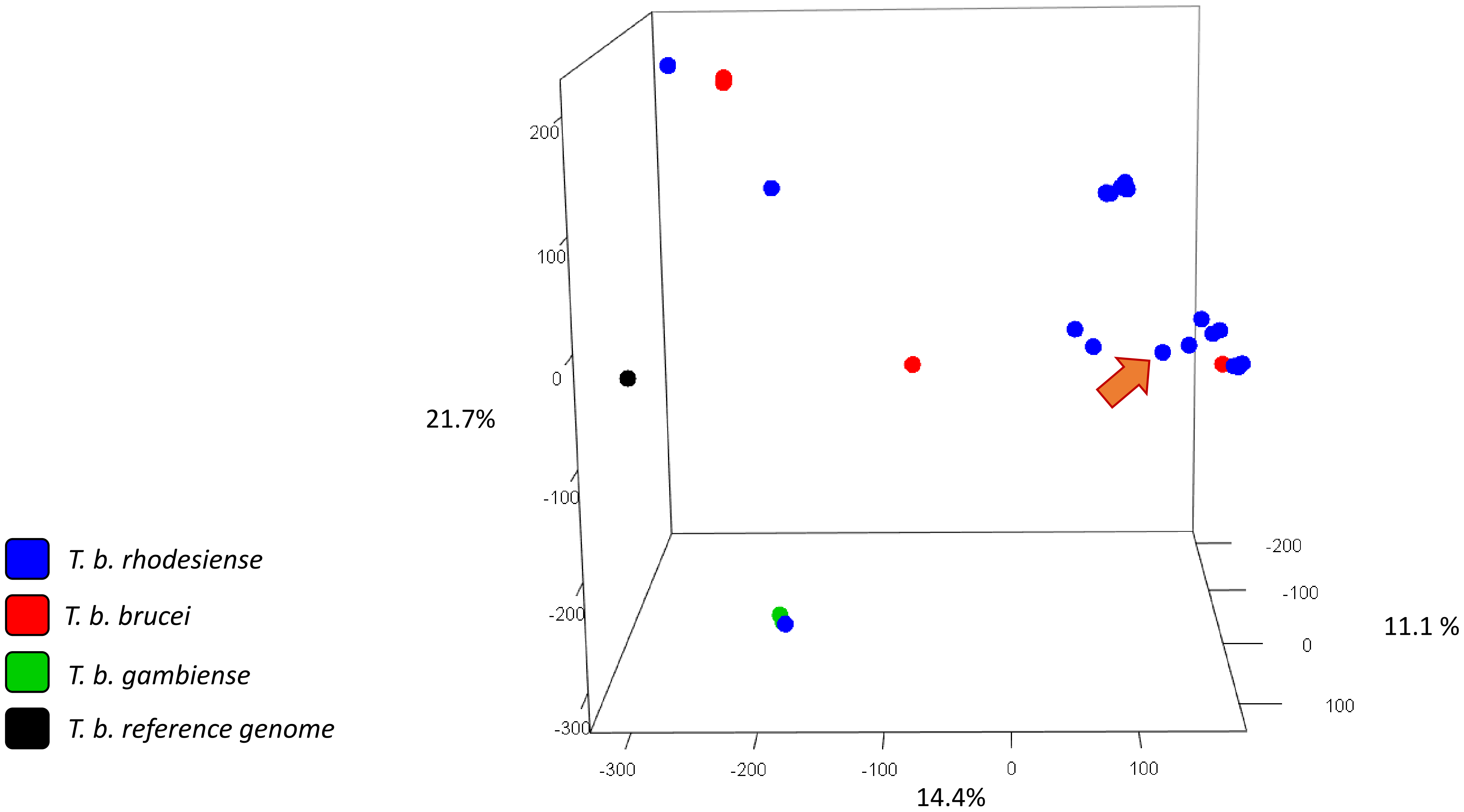

Supplementary Figure 1: PCA of *T. brucei* isolates' genomic distance based on differential SNPs. The genomic distance estimated by three dimension principal component analysis of the 26 *T. brucei* isolates is shown in different angles. This three dimensional representation comprises 47.2% of the variability observed in the evaluated *T. brucei* Isolates. *T. b. rhodesiense* isolates are represented in blue, while *T. b. brucei* are in red, and *T. b. gambiense* in green. The black circle correspond to the *T. b. brucei* 927 reference genome, used as template in the SNP calling. The red arrow points to the D11 isolate, the only potentially trisomic *T. brucei* isolate evaluated.

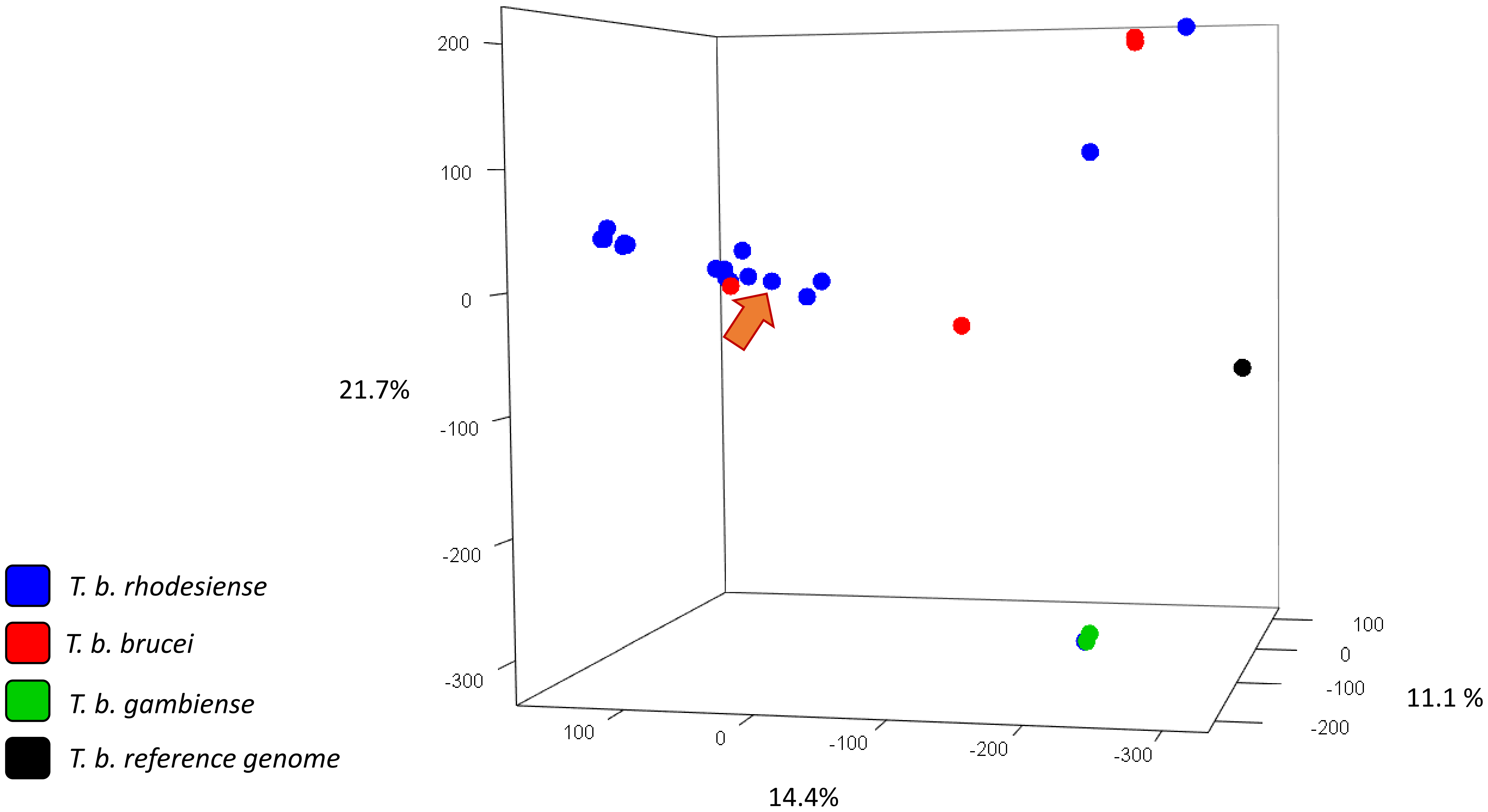

Supplementary Figure 1: PCA of *T. brucei* isolates' genomic distance based on differential SNPs. The genomic distance estimated by three dimension principal component analysis of the 26 *T. brucei* isolates is shown in different angles. This three dimensional representation comprises 47.2% of the variability observed in the evaluated *T. brucei* Isolates. *T. b. rhodesiense* isolates are represented in blue, while *T. b. brucei* are in red, and *T. b. gambiense* in green. The black circle correspond to the *T. b. brucei* 927 reference genome, used as template in the SNP calling. The red arrow points to the D11 isolate, the only potentially trisomic *T. brucei* isolate evaluated.

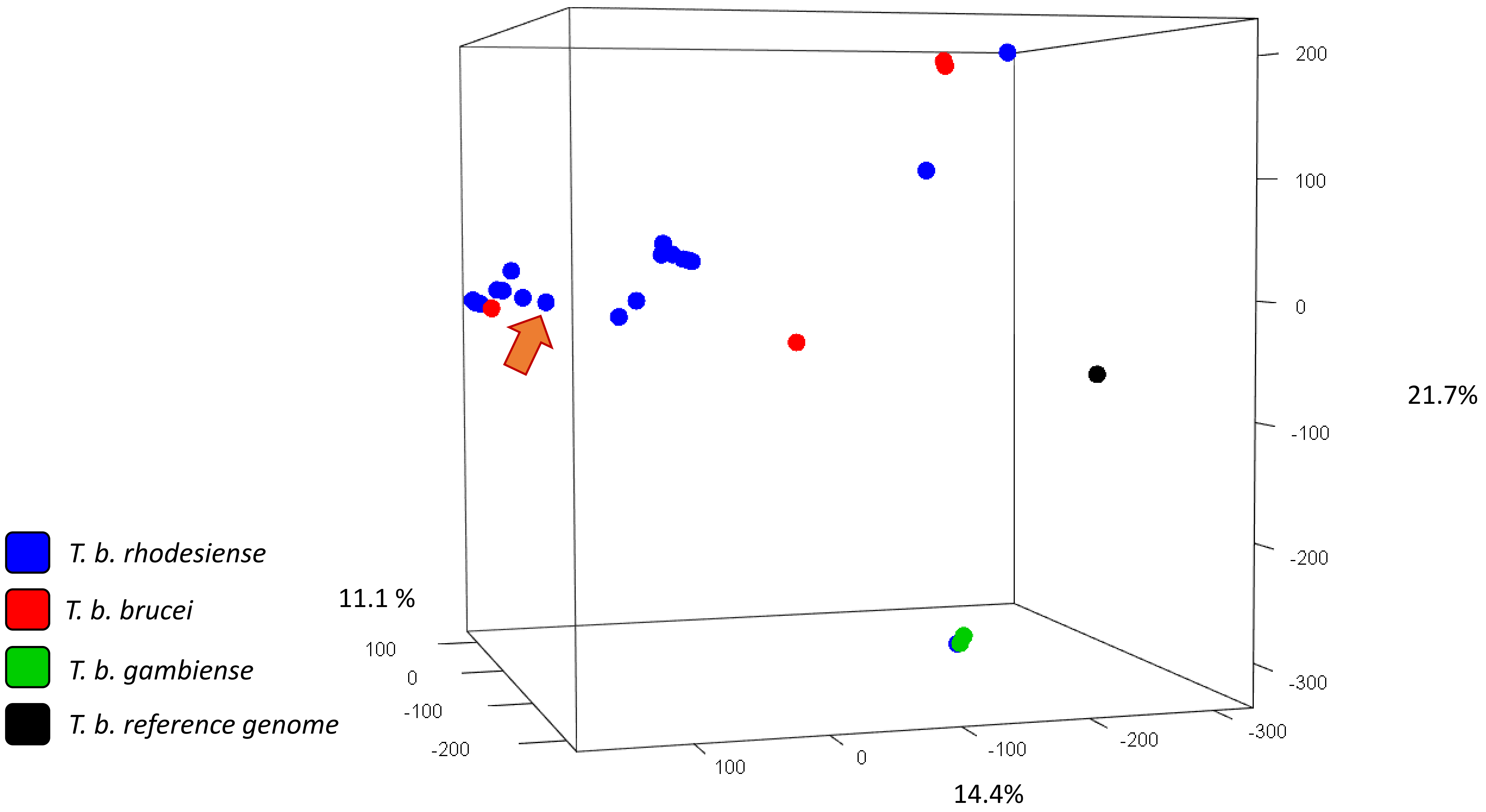

Supplementary Figure 2: Impact of the genome coverage in allele frequency ploidy estimations. To evaluate the impact of the genome coverage in the allele frequency ploidy estimations, we evaluated the allele frequency of the D11 isolate using 25% **(A)**, 50% **(B)**, or 75% **(C)** of its reads, corresponding to the genome coverages of 6.92, 13.84 and 20.76x, respectively. The peaks in 0.33 and 0.66 that are suggestive of partial triploidy are observed with ~20x coverage, similarly to what is observed with 100% of the reads **(D)**. From the 26 *T. brucei* samples evaluated, only D1 **(E)** and ytat **(F)** had coverages lower than 20x, but only D11 had peaks suggestive of partial triploidy in the parasite population.

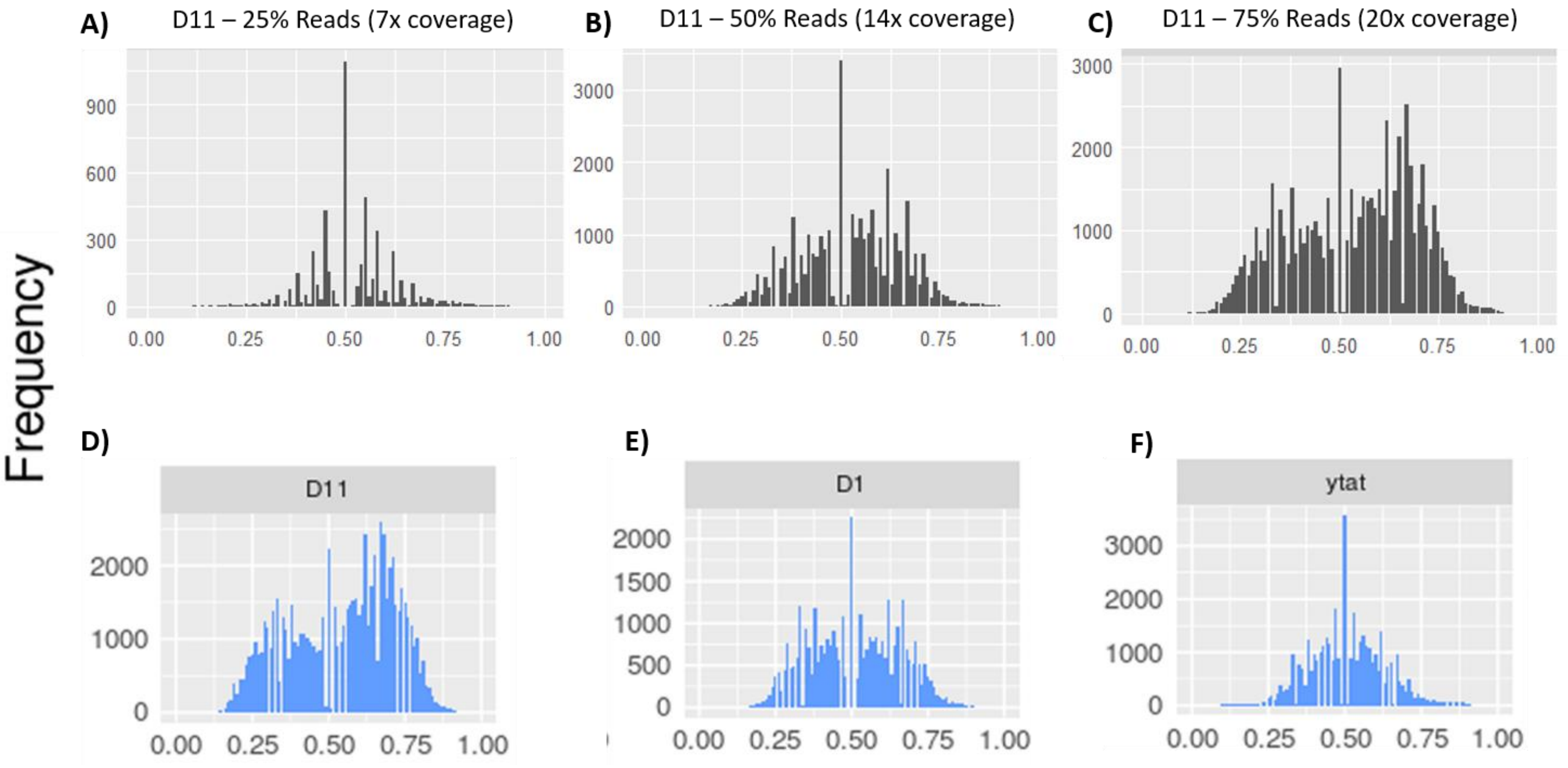

Supplementary Figure 3: Chromosomal somy estimated based on allele frequency. For each chromosome, the proportion of the alleles in each predicted heterozygous site was obtained and rounded to the second place. Base frequencies were rounded in 100 categories, ranging from 0.01 to 1.00, and an approximate distribution of base frequencies for each chromosome was plotted in R. Diploid chromosomes had a peak in 0.50 **(A)**, while triploid chromosomes had peaks in 0.33 and 0.66 **(B)**. Tetraploid chromosomes had combination of peaks of 0.25, 0.75 and 0.50 **(C)**. Each slide correspond to somy predictions of the eleven *T. brucei* chromosomes, based on each of the read libraries described in table 1. The *T. brucei* subspecies and isolate/strain name is described in the top of each slide.

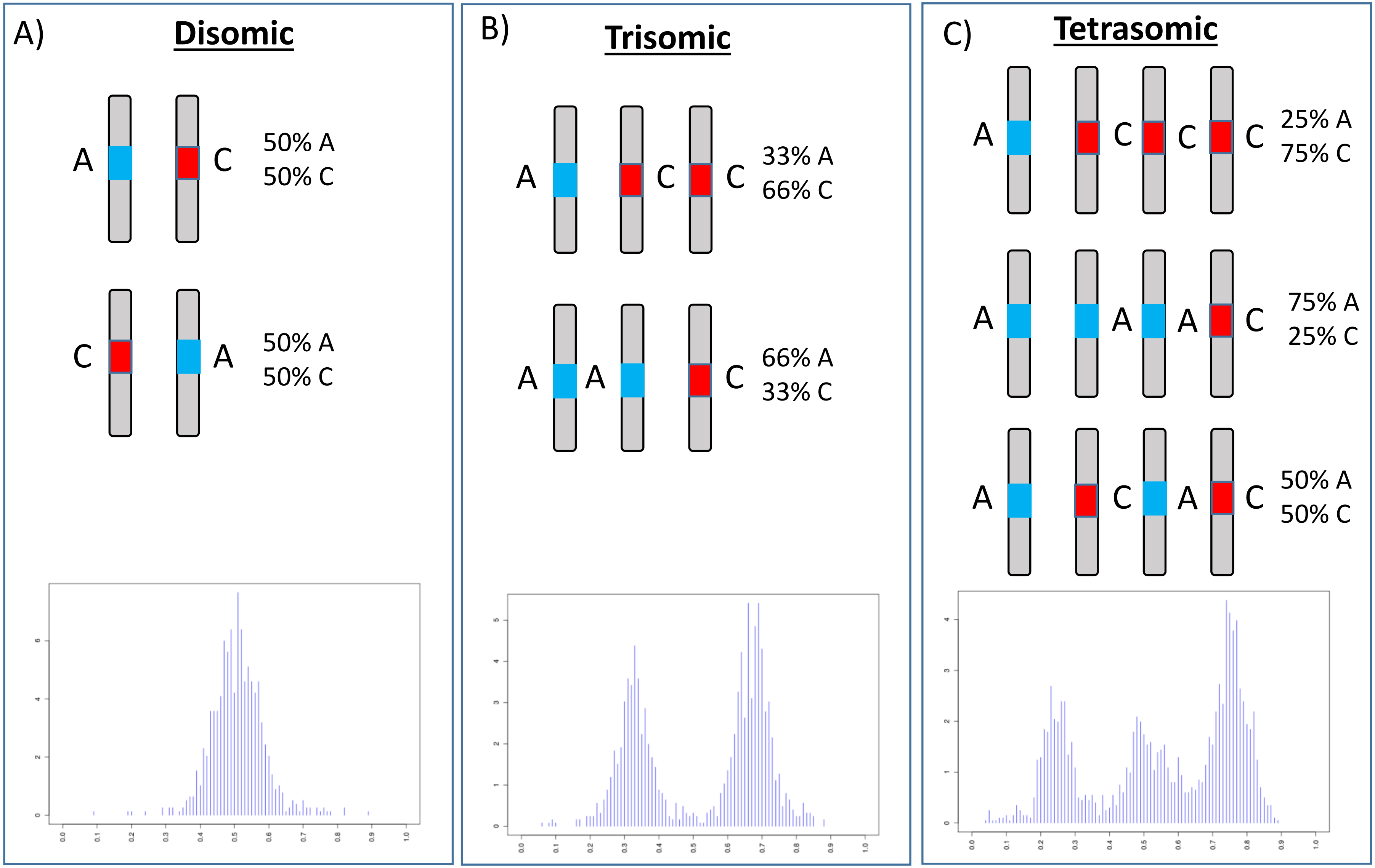

T. b. rhodesiense **- Isolate D3**

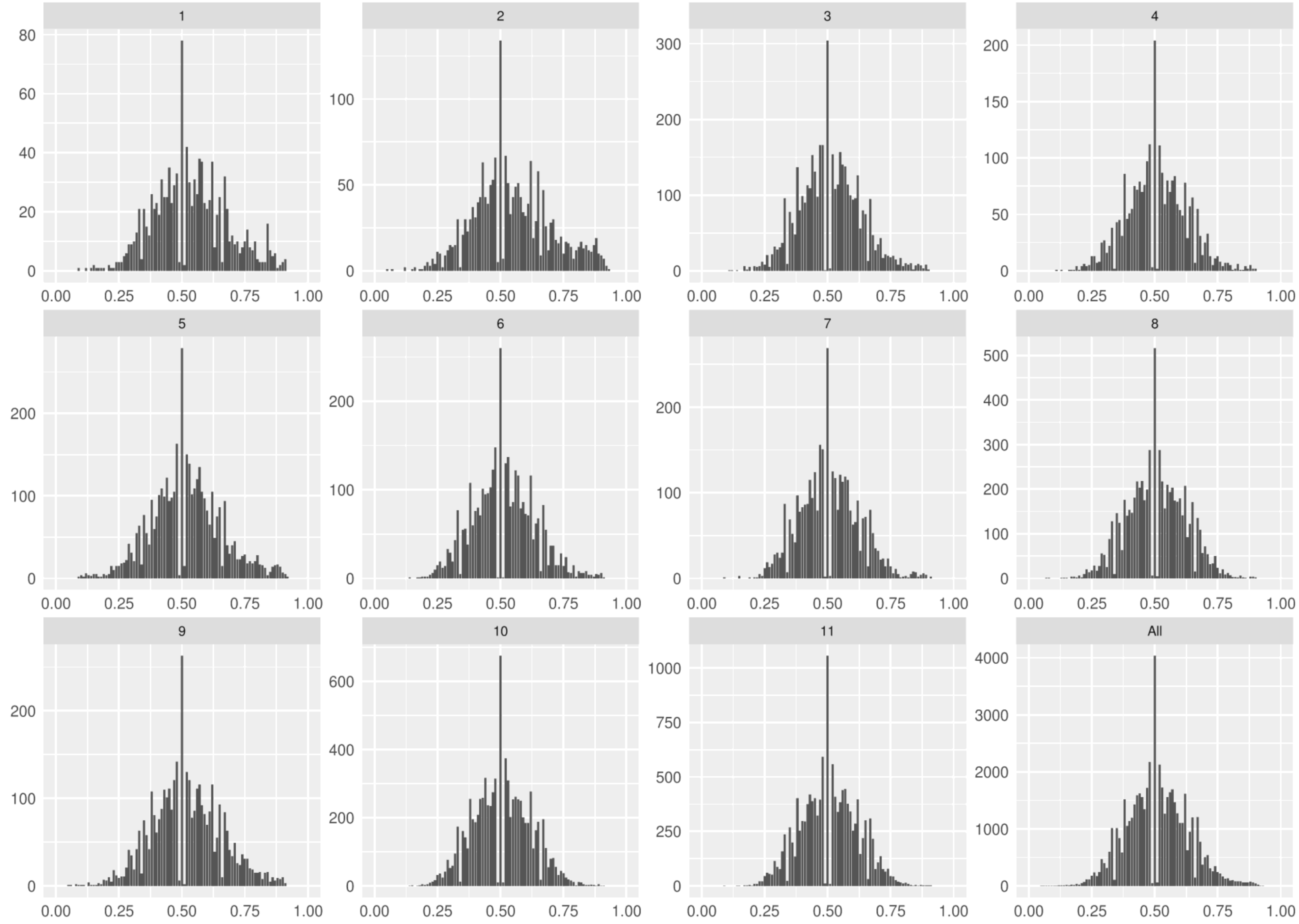

***T. b. rhodesiense* - Isolate D4**

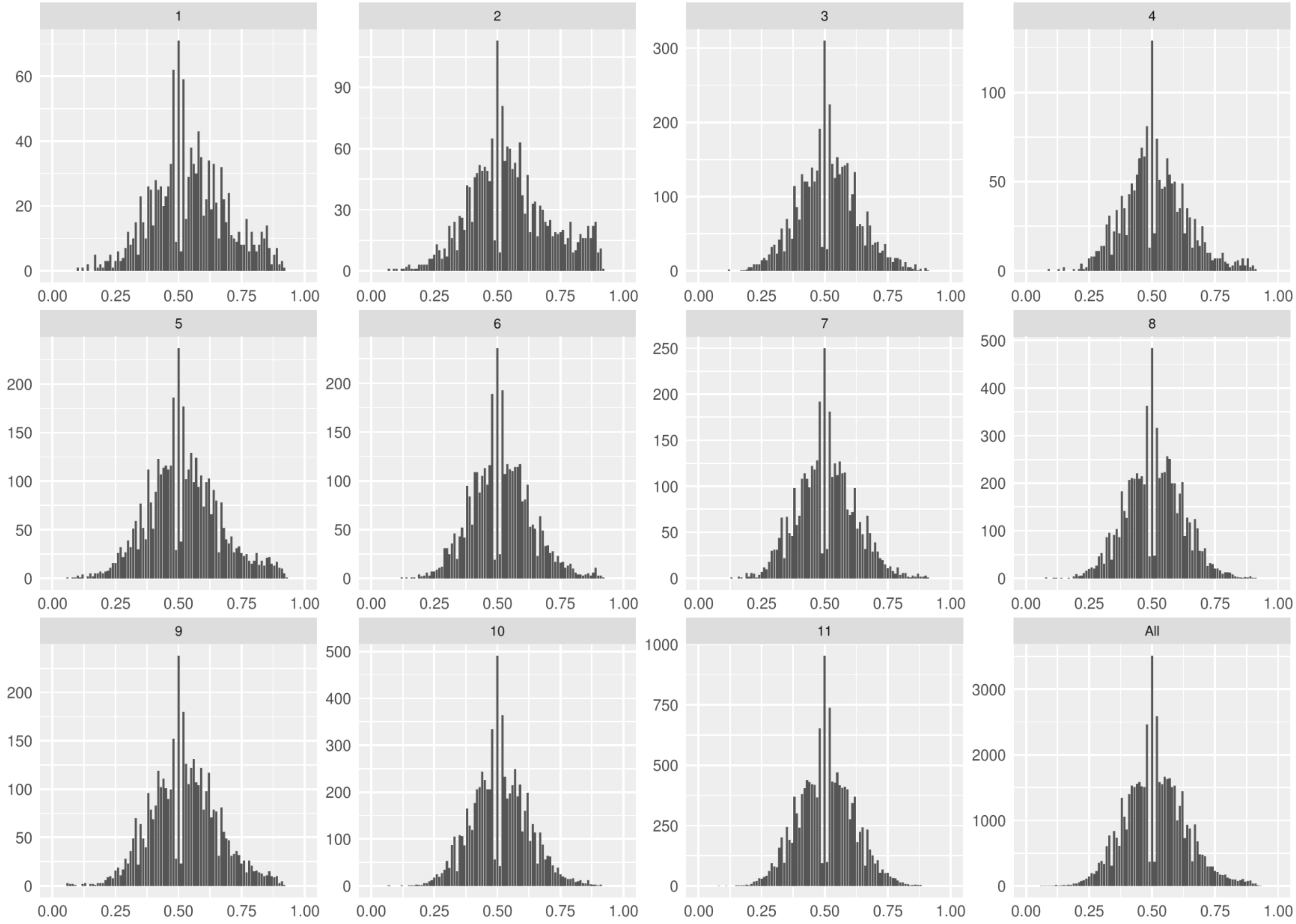

T. b. rhodesiense **- Isolate Apendum**

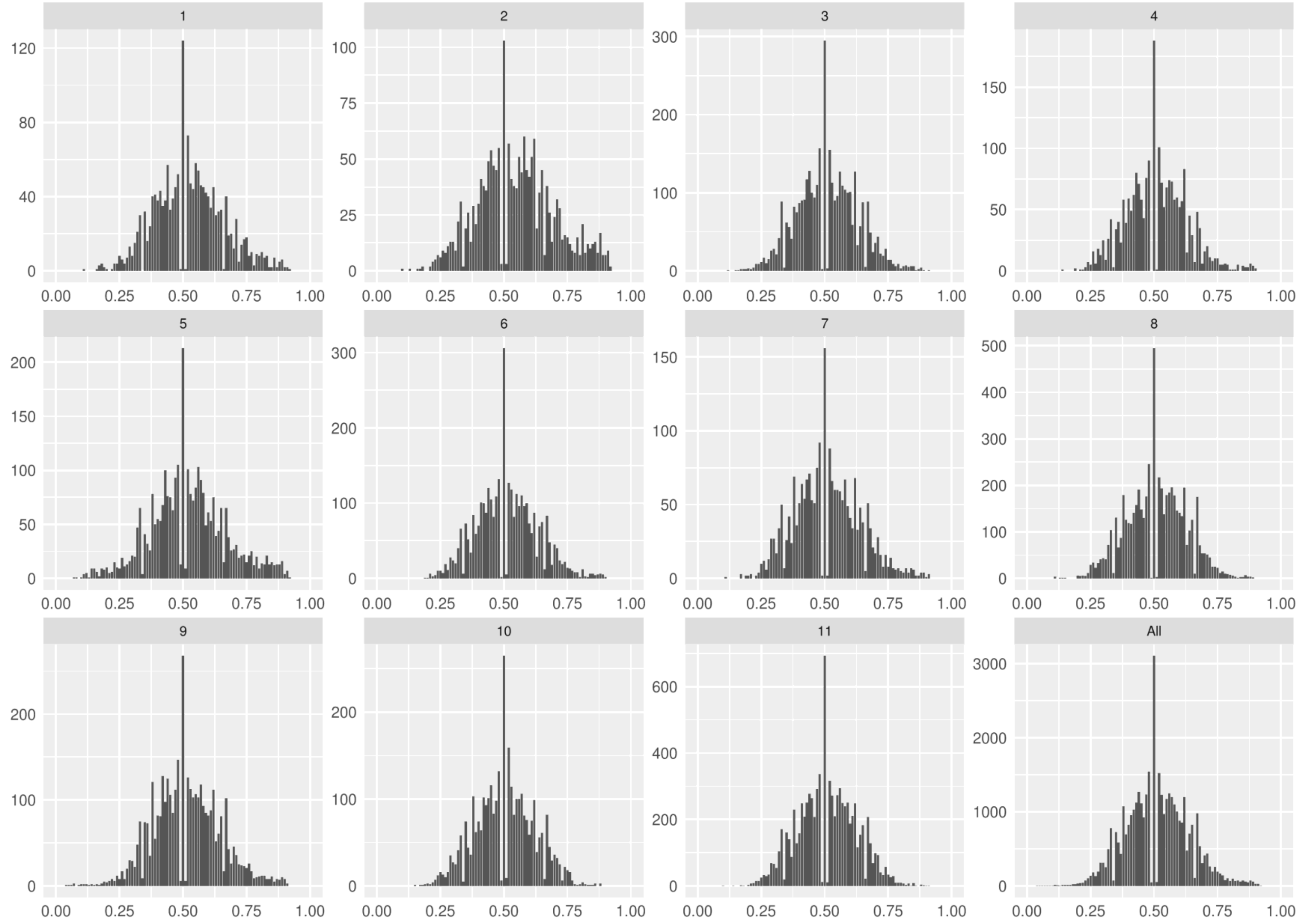

***T. b. rhodesiense* - Isolate D16**

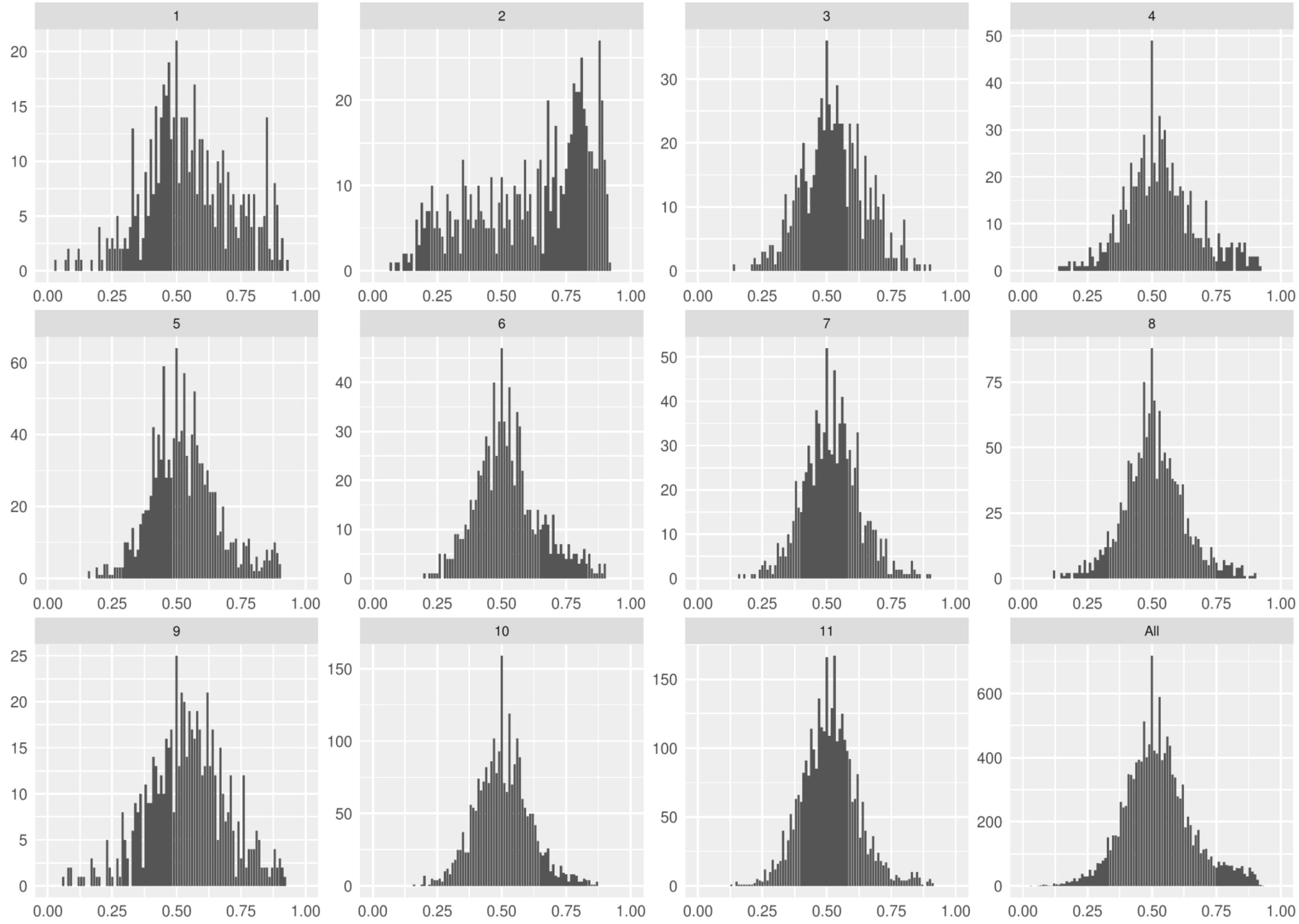

T. b. rhodesiense - Isolate D2

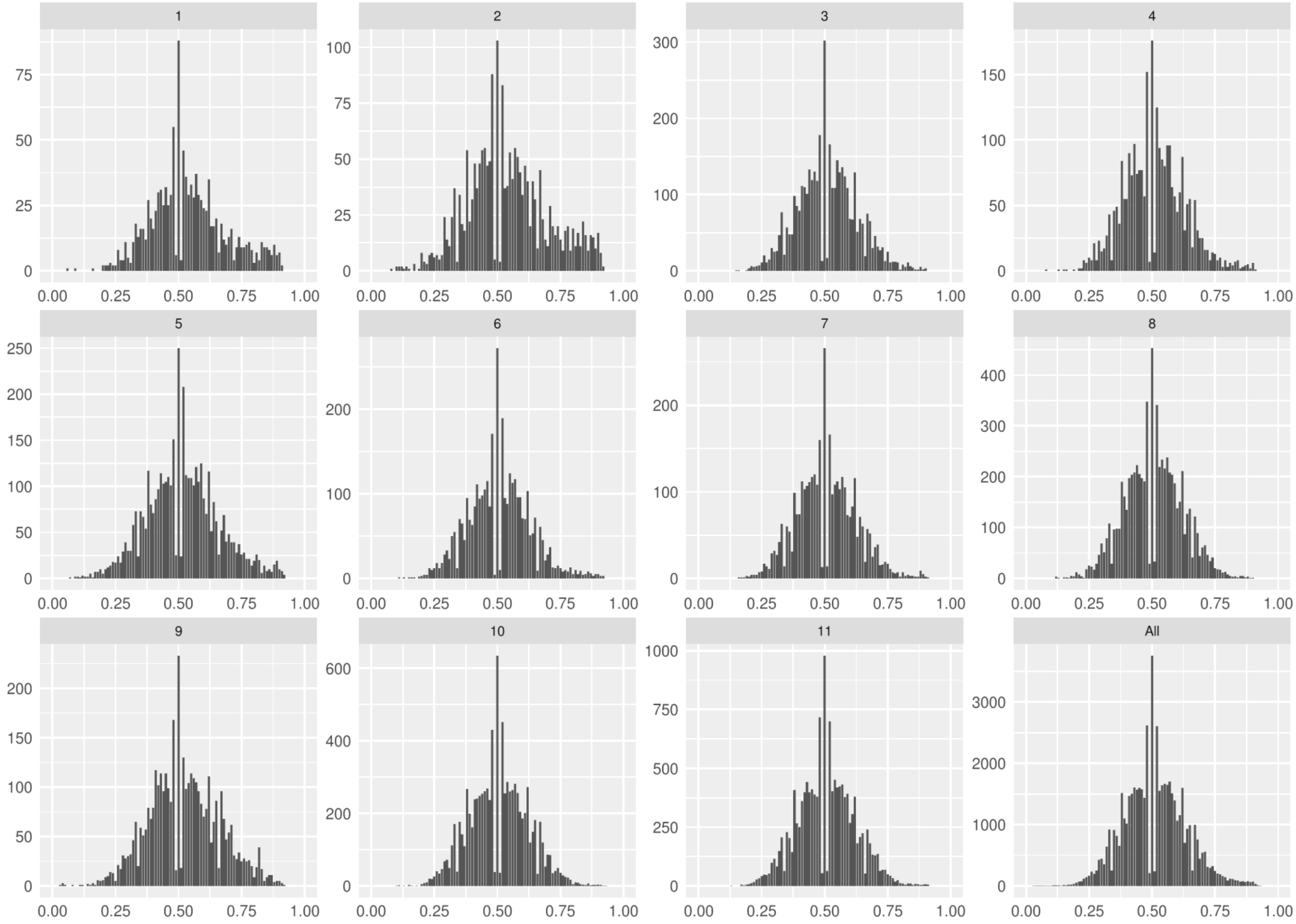

*T. b. rhodesiense* - Isolate D1

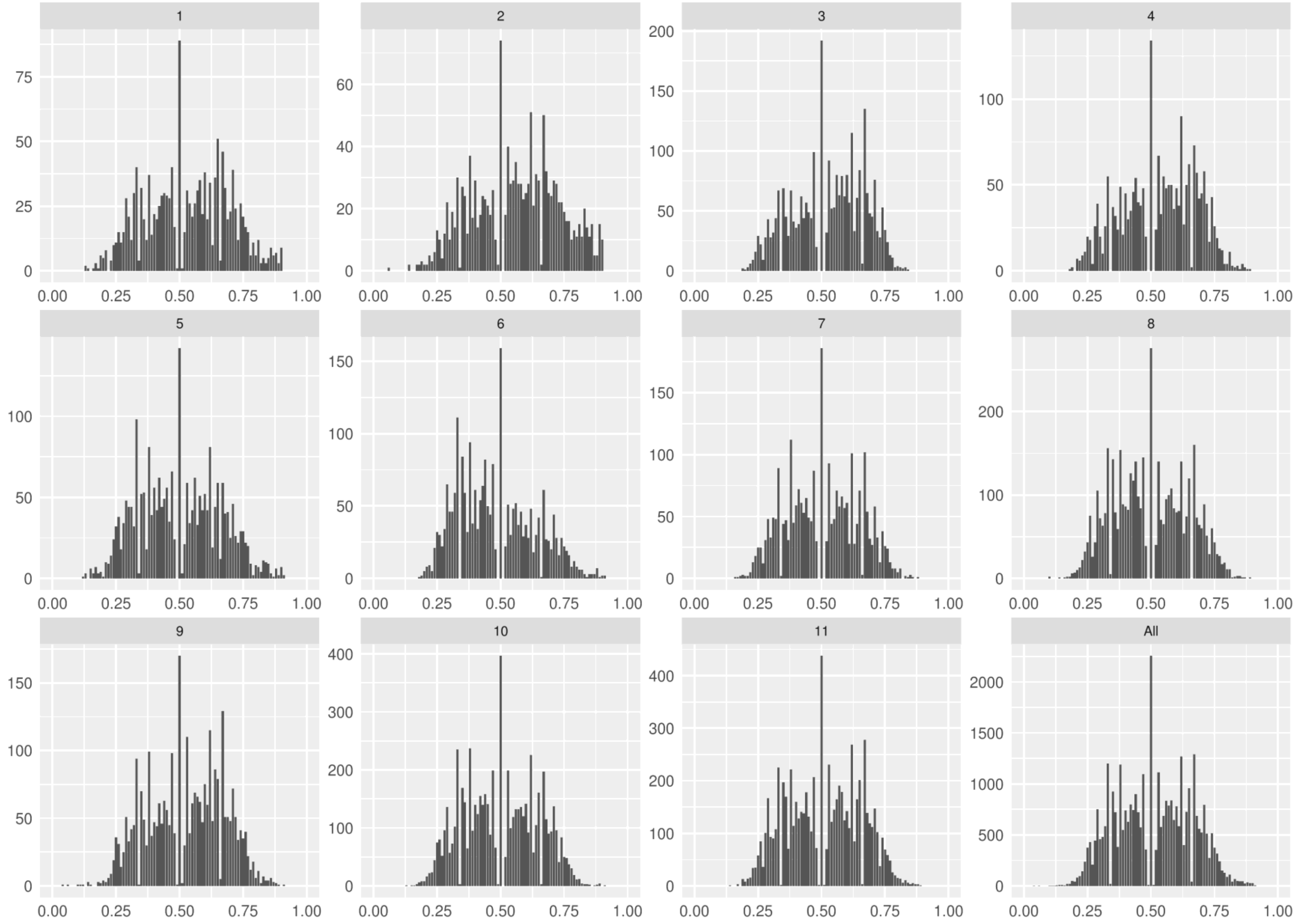

T. b. rhodesiense - Isolate D11

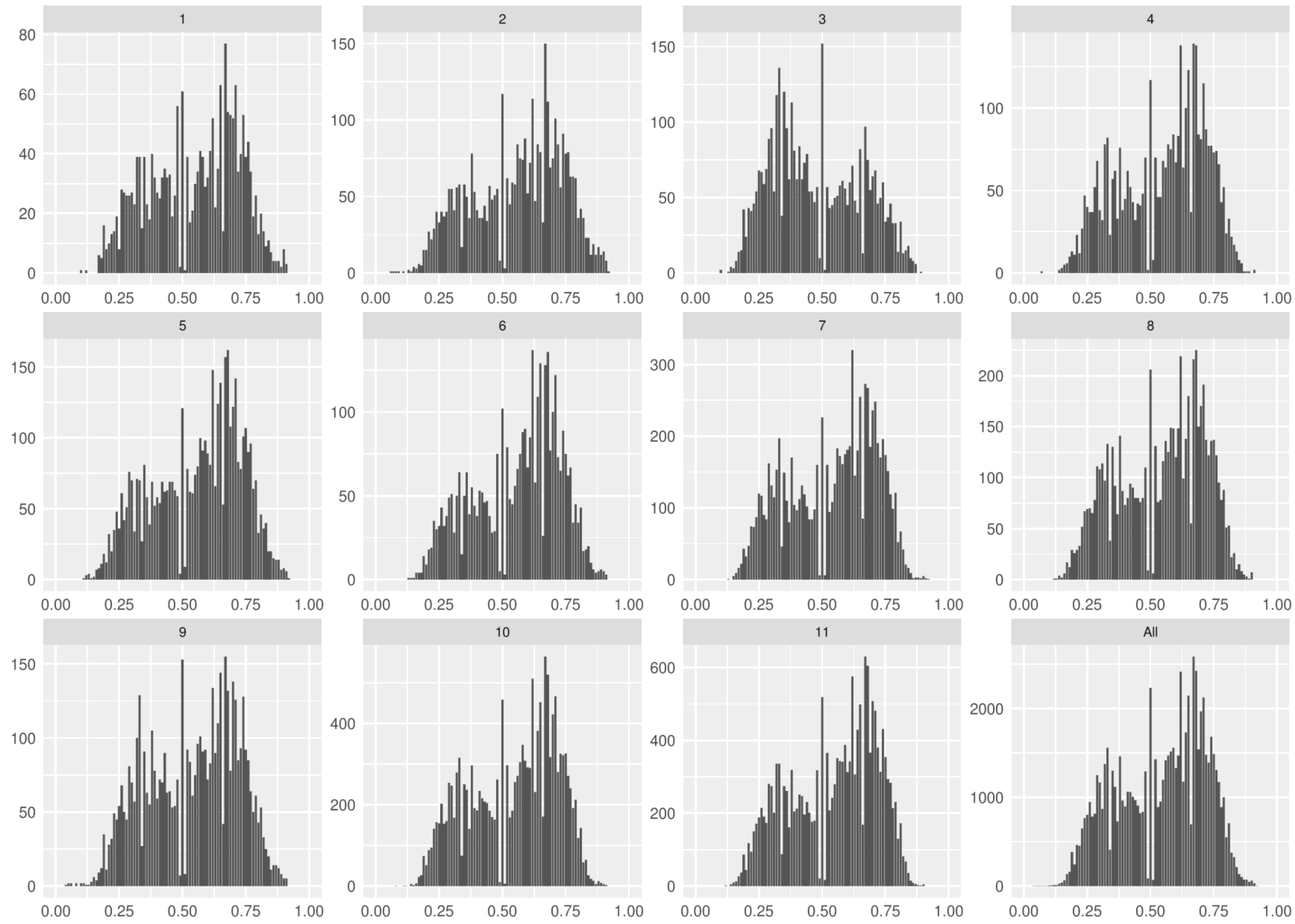

T. b. rhodesiense - Isolate STIB900

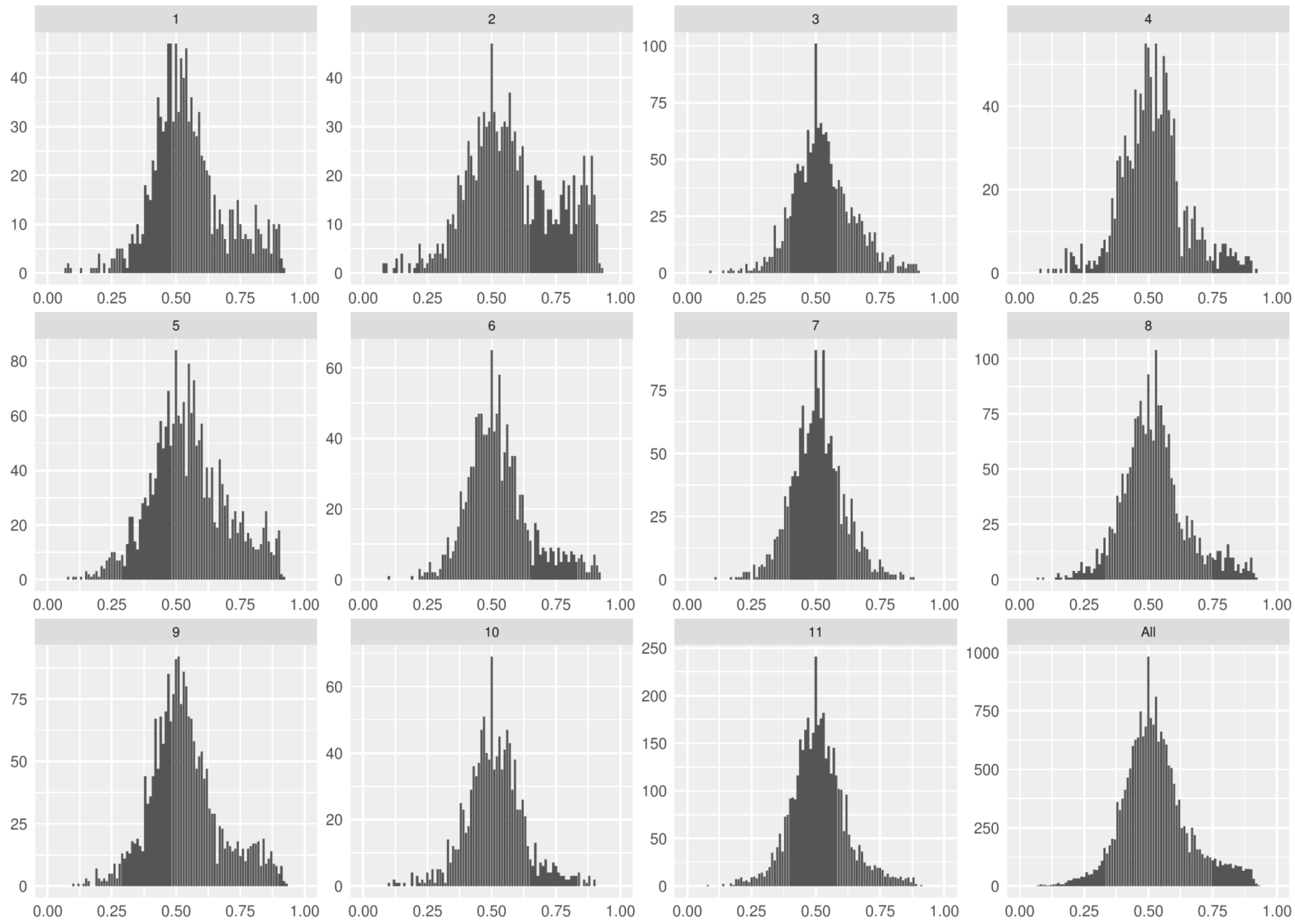

T. b. rhodesiense - Isolate STIB704C

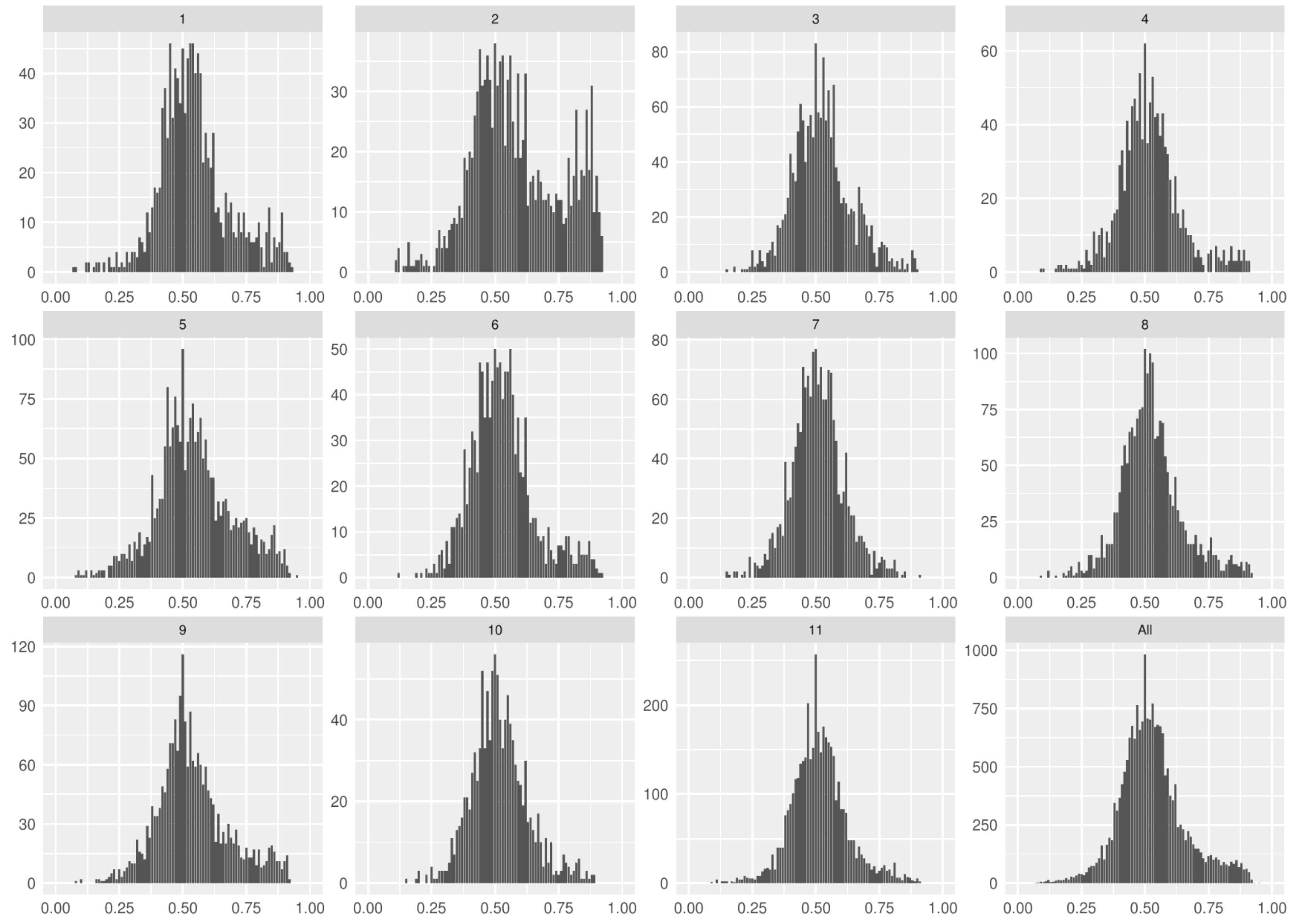

T. b. rhodesiense - Isolate ytat

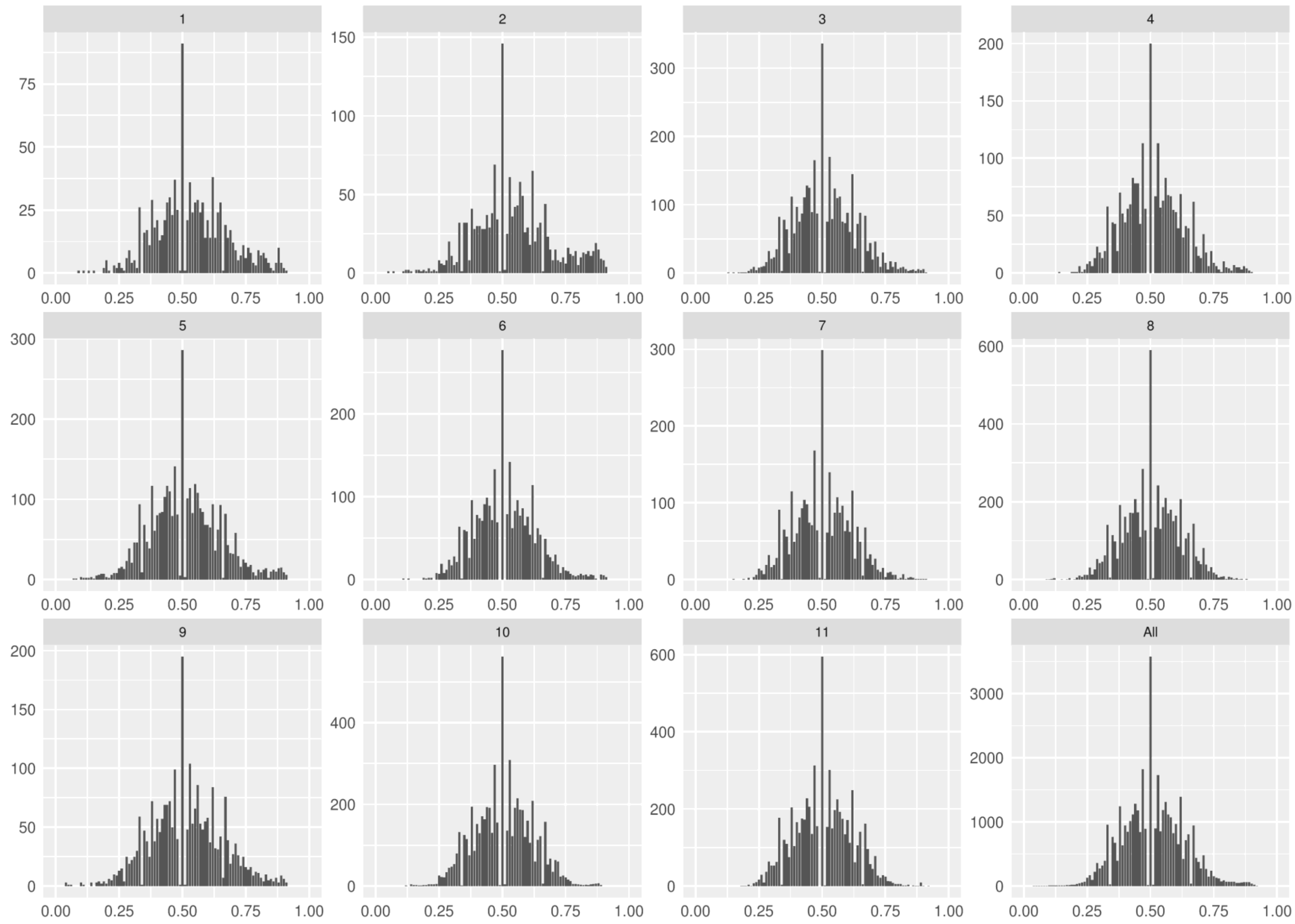

T. b. rhodesiense **- Isolate LWO30A**

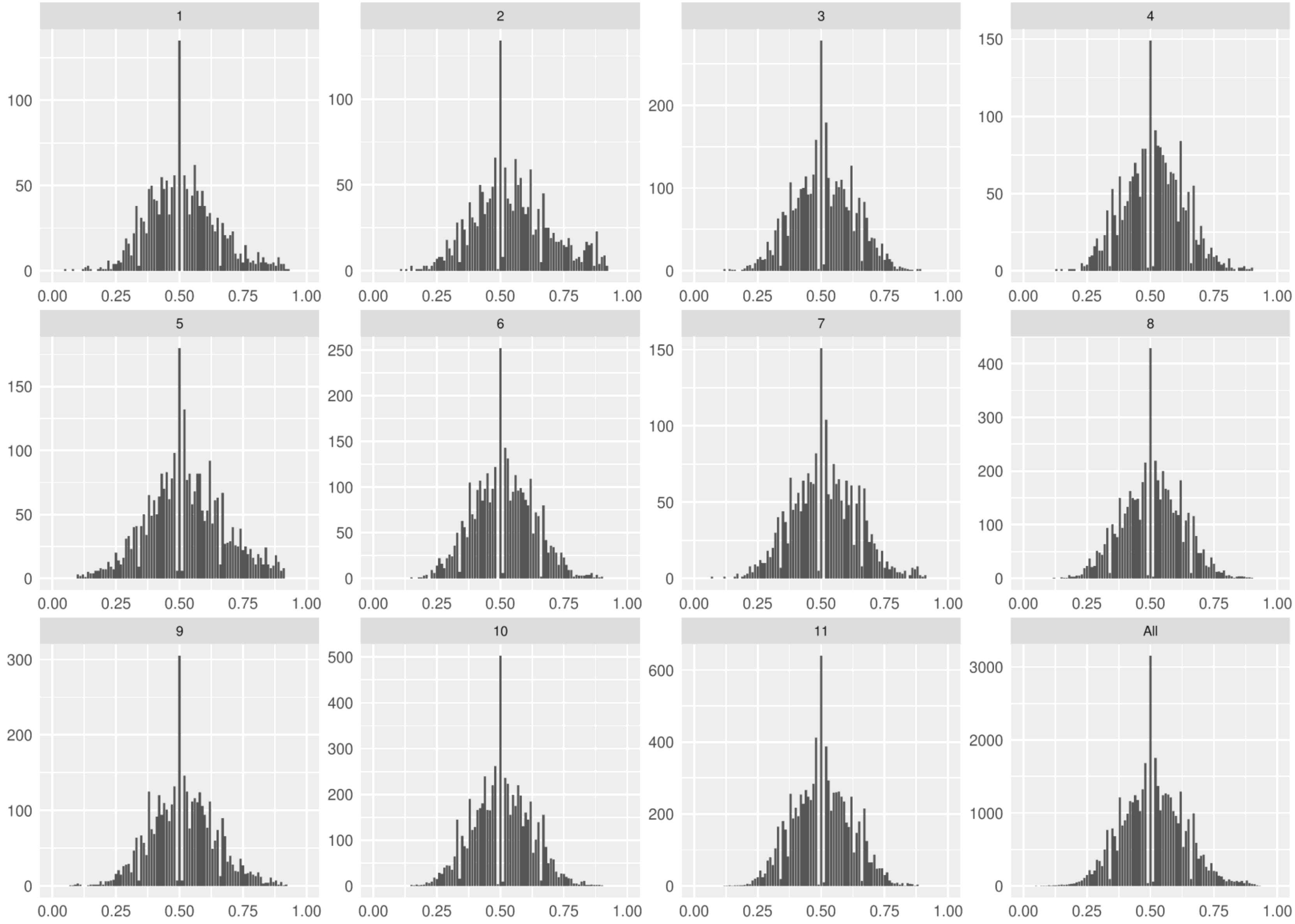

T. b. rhodesiense - Isolate Okware

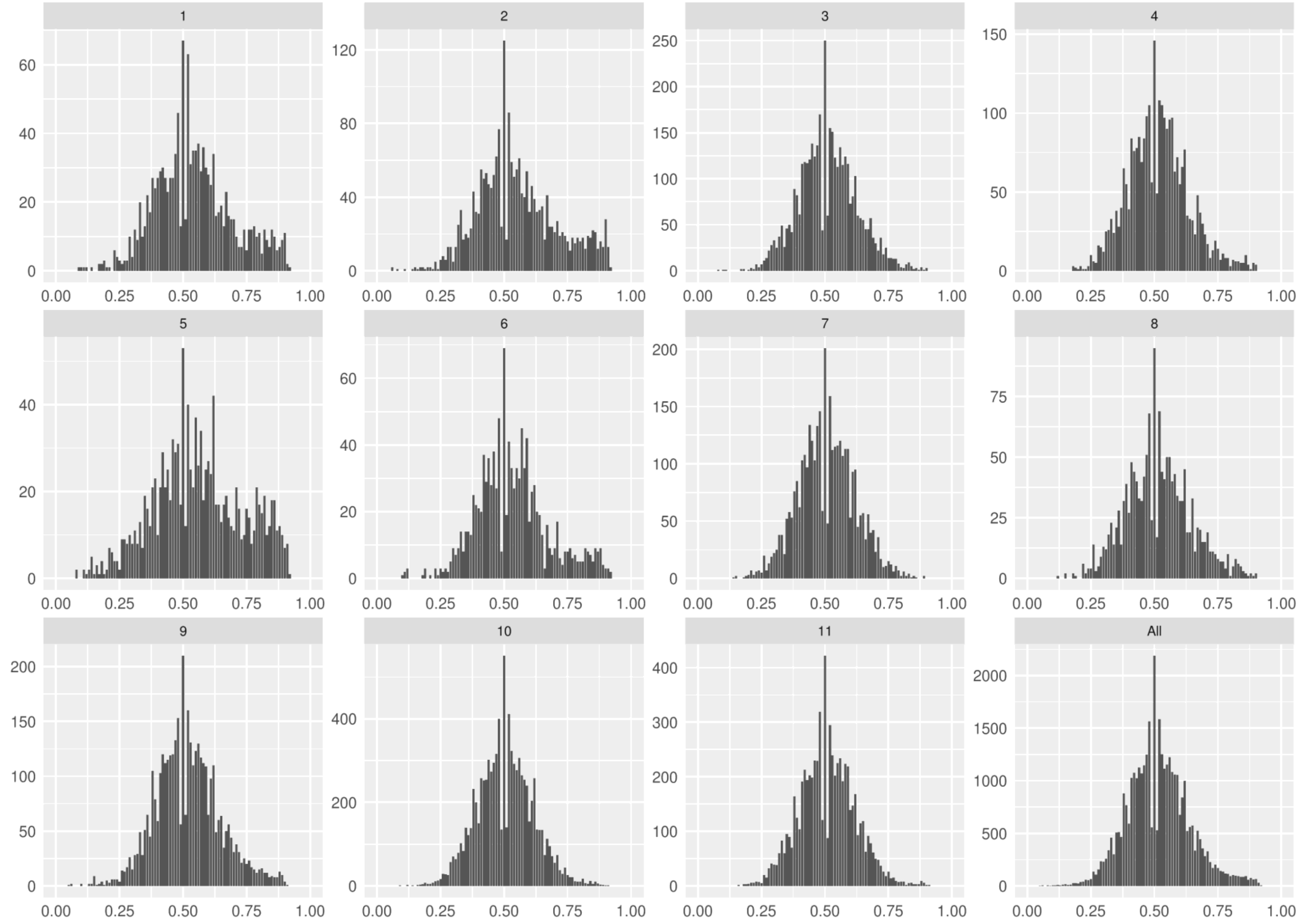

T. b. rhodesiense **- Isolate Dog157**

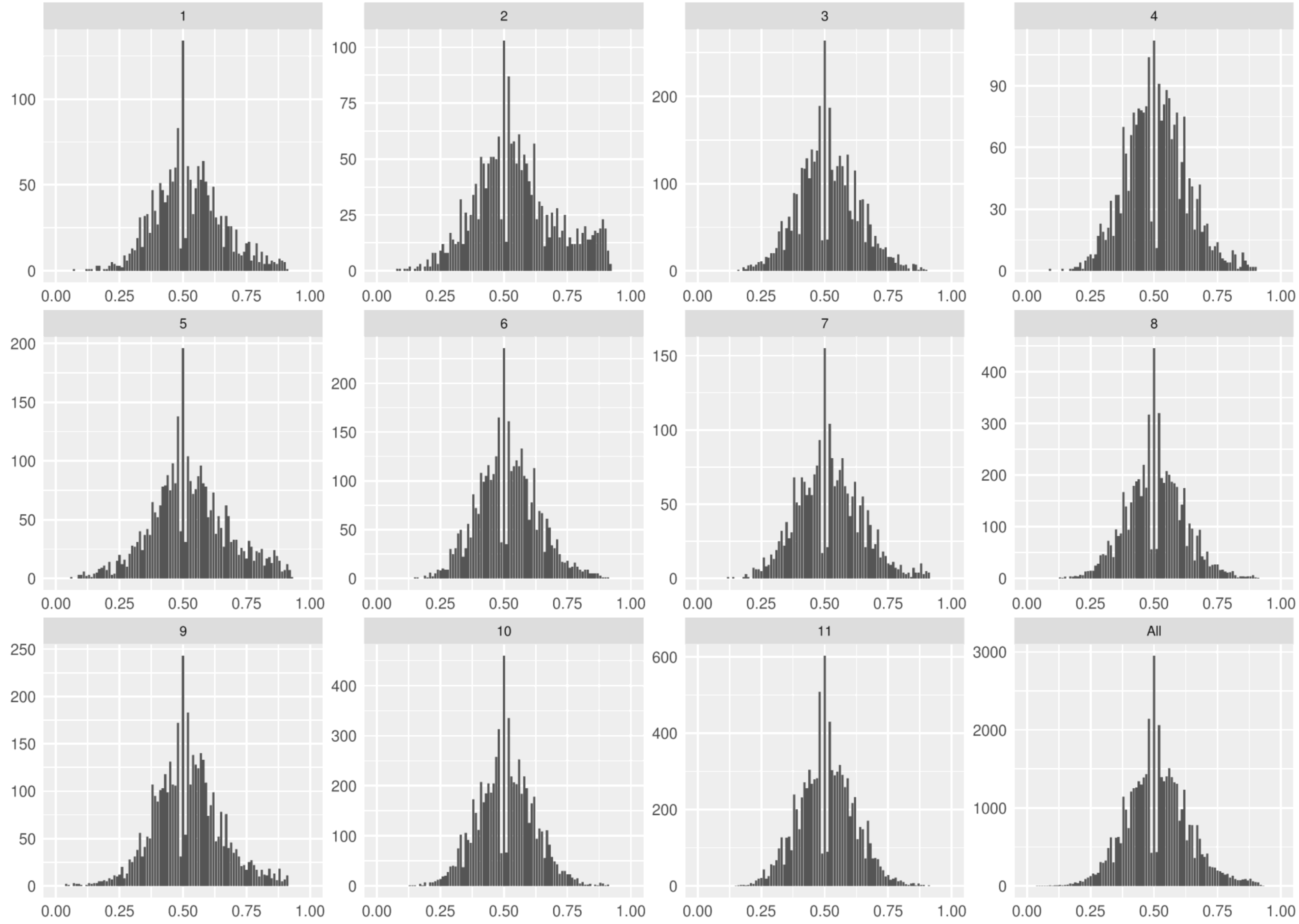

T. b. rhodesiense **- Isolate Keko**

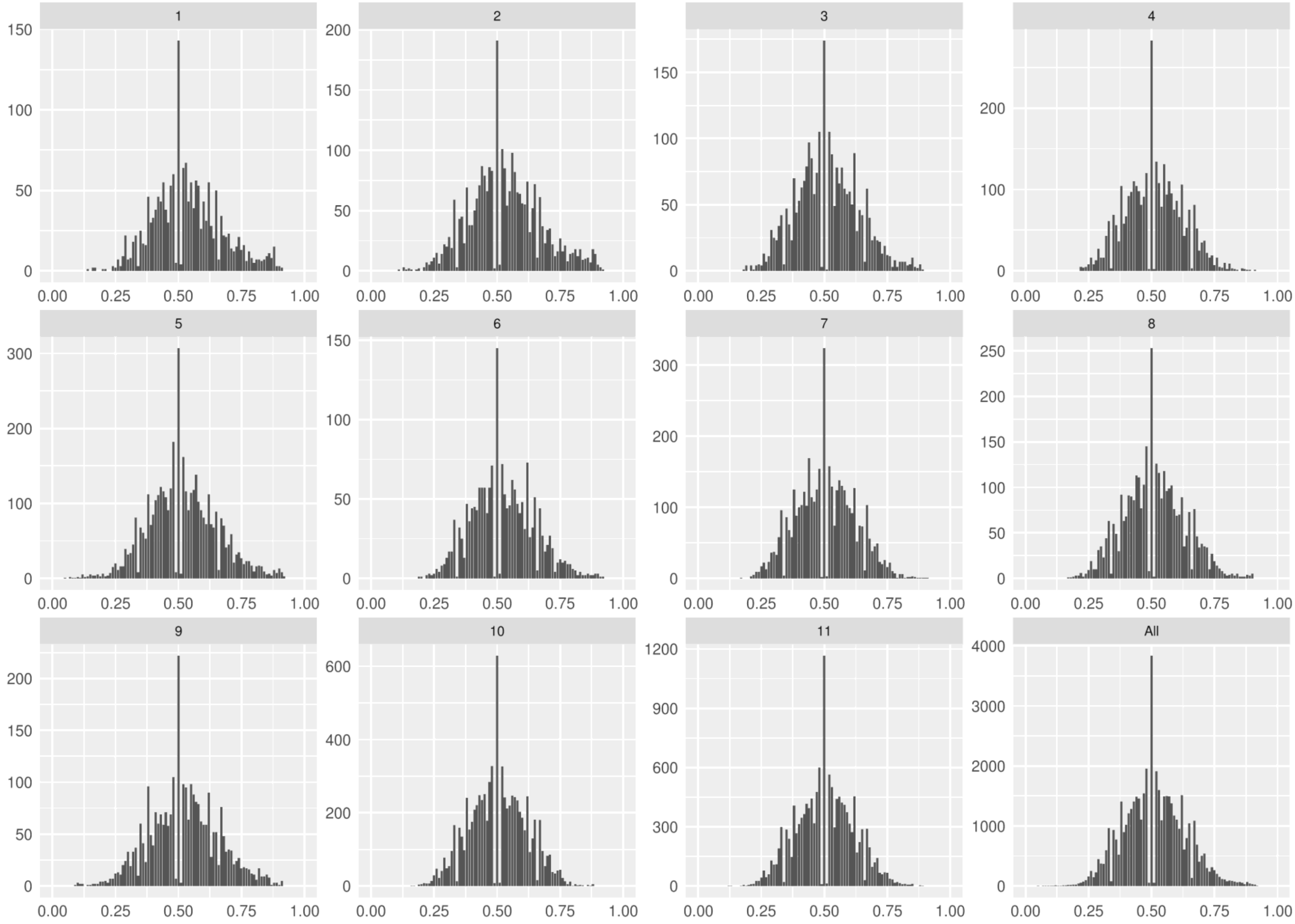

T. b. rhodesiense **- Isolate D5**

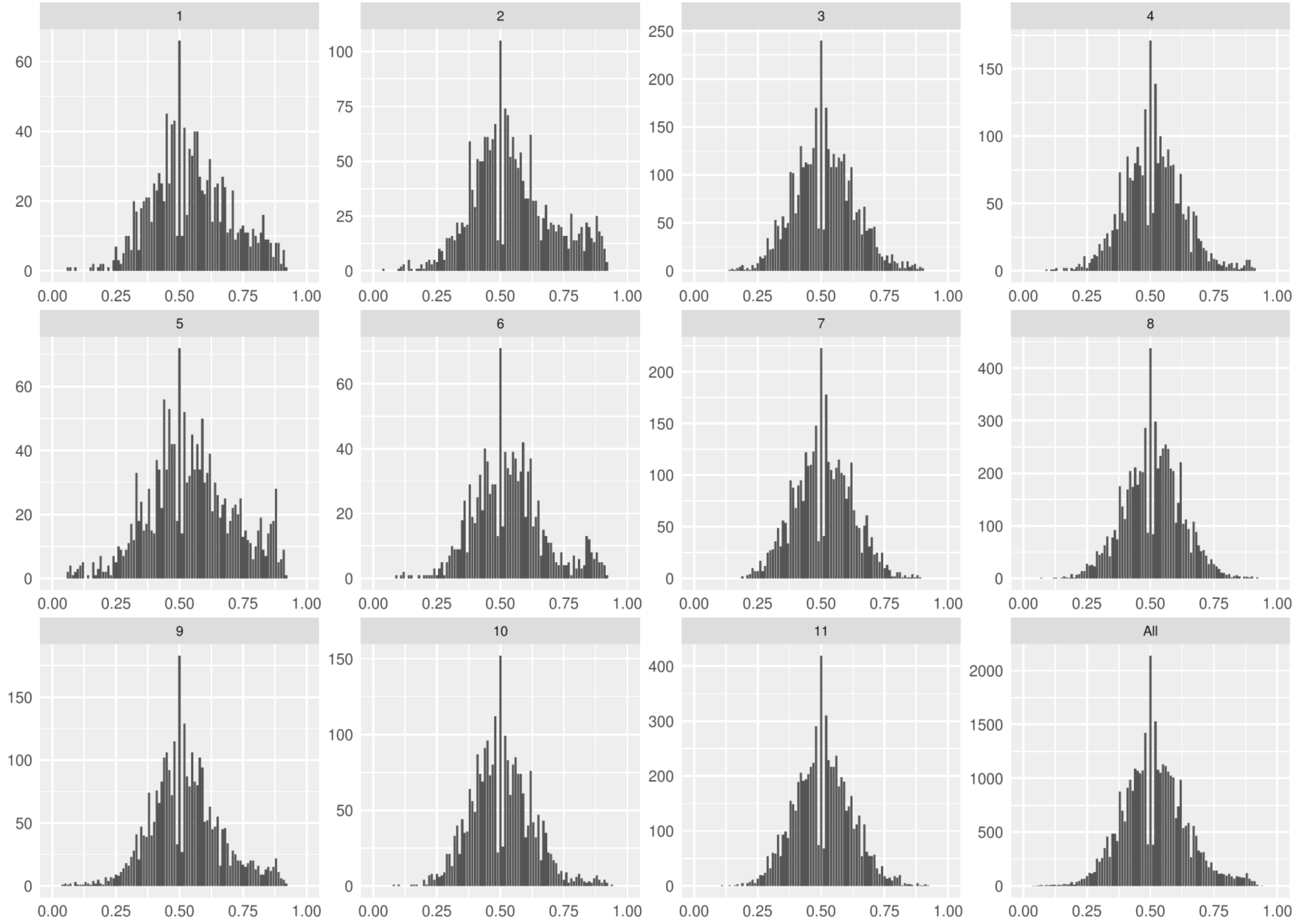

T. b. rhodesiense - Isolate D7

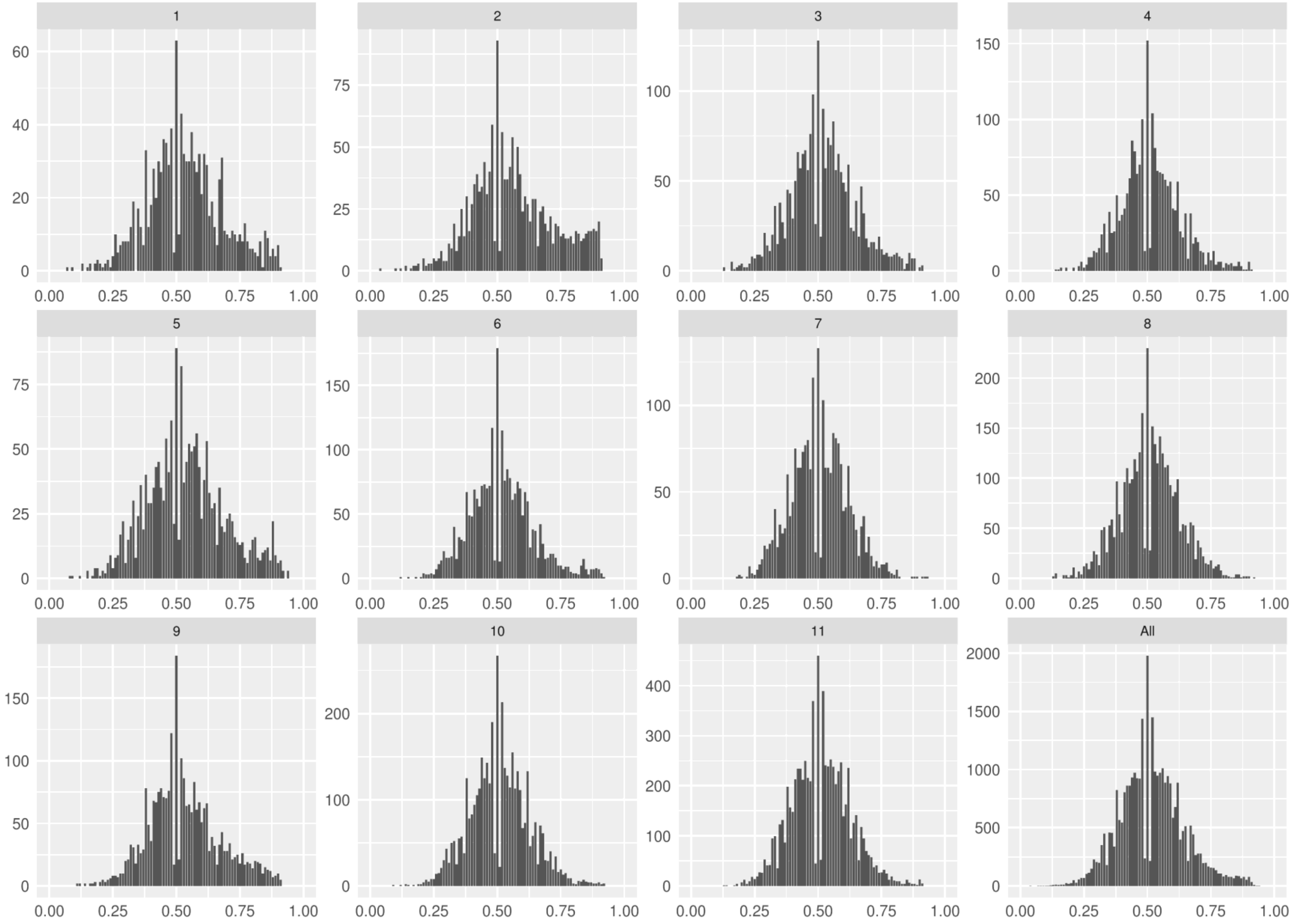

T. b. rhodesiense **- Isolate LWO150A**

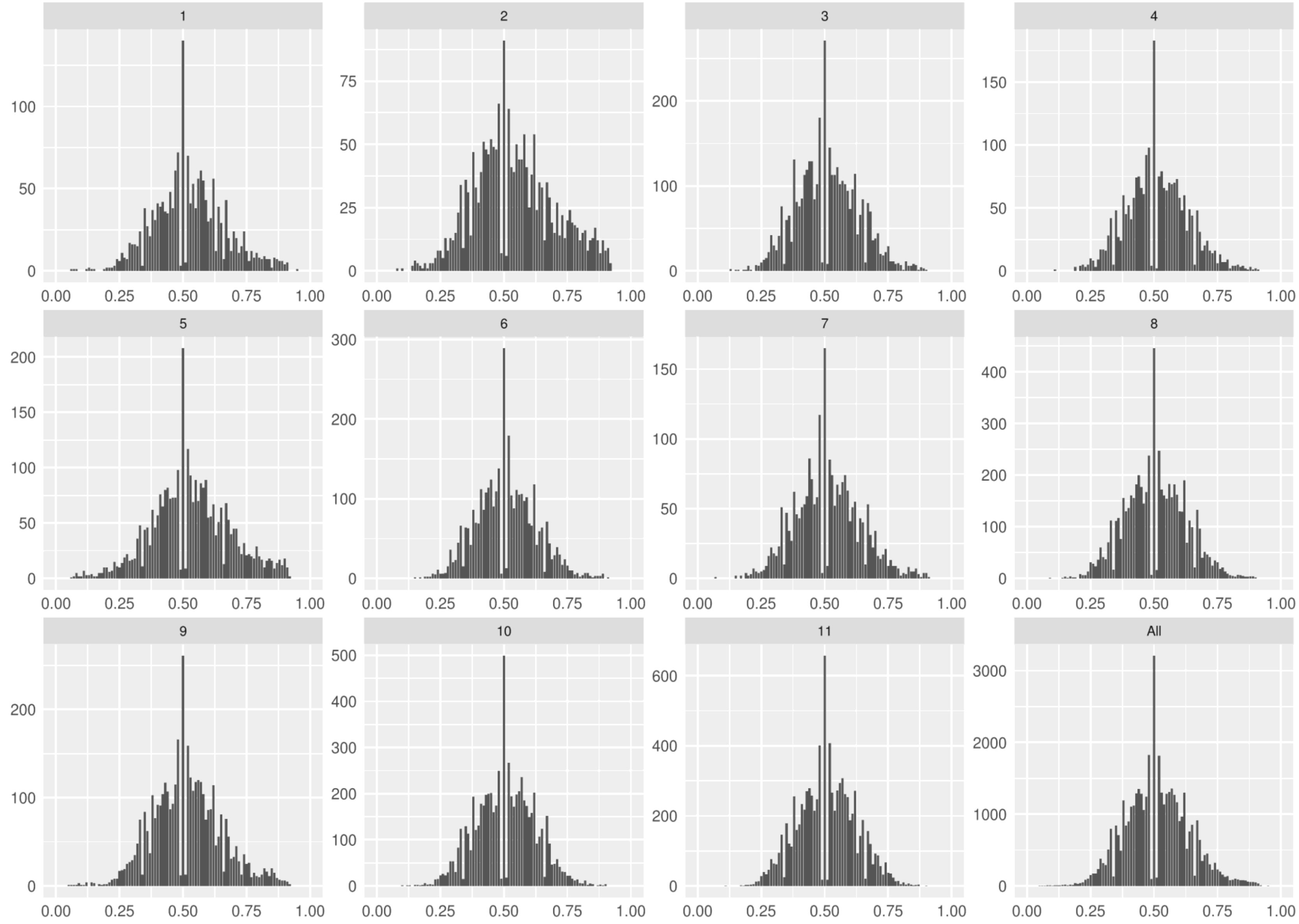

T. b. rhodesiense **- Isolate LWO24A**

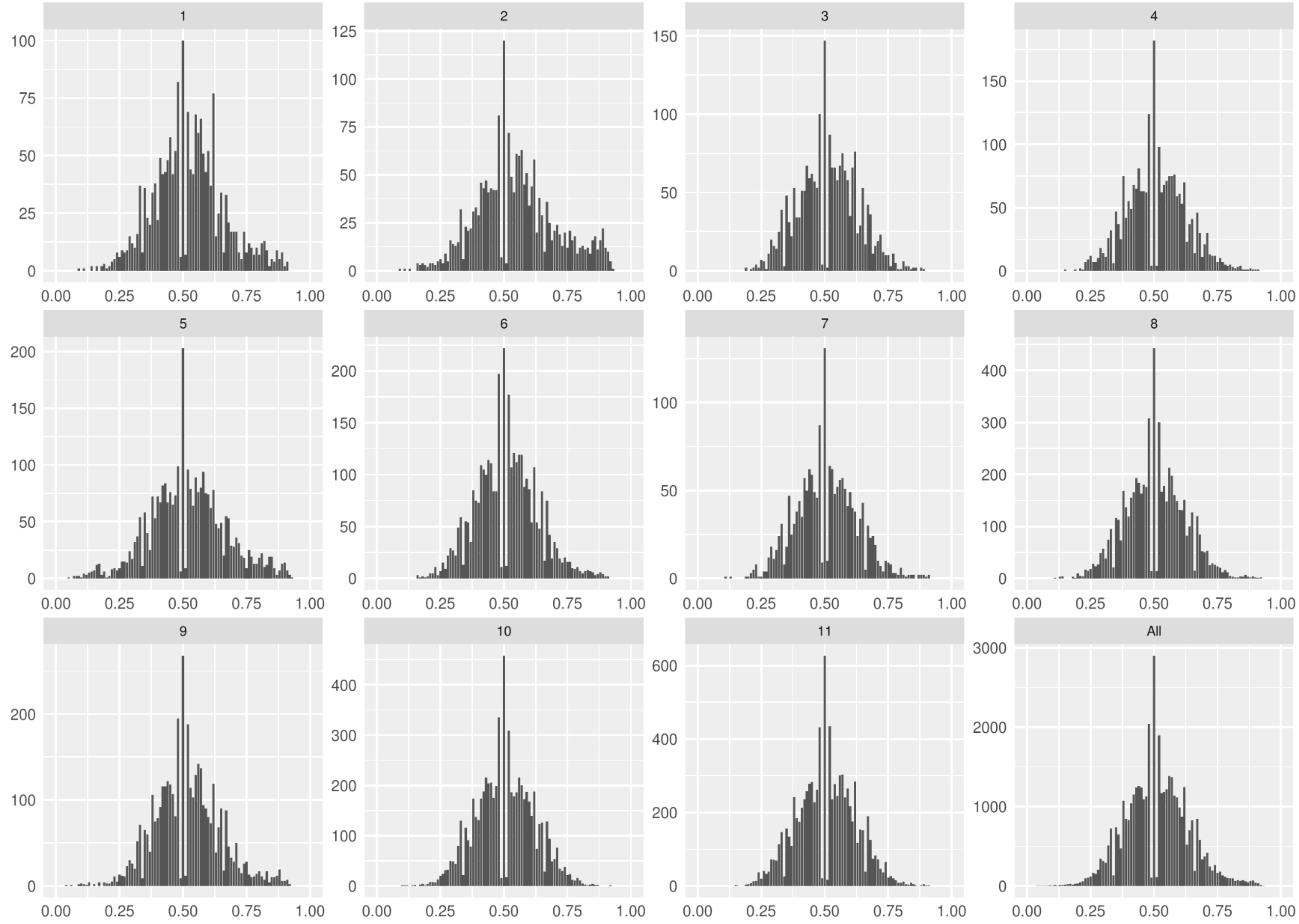

T. b. rhodesiense **- Isolate LWO07A**

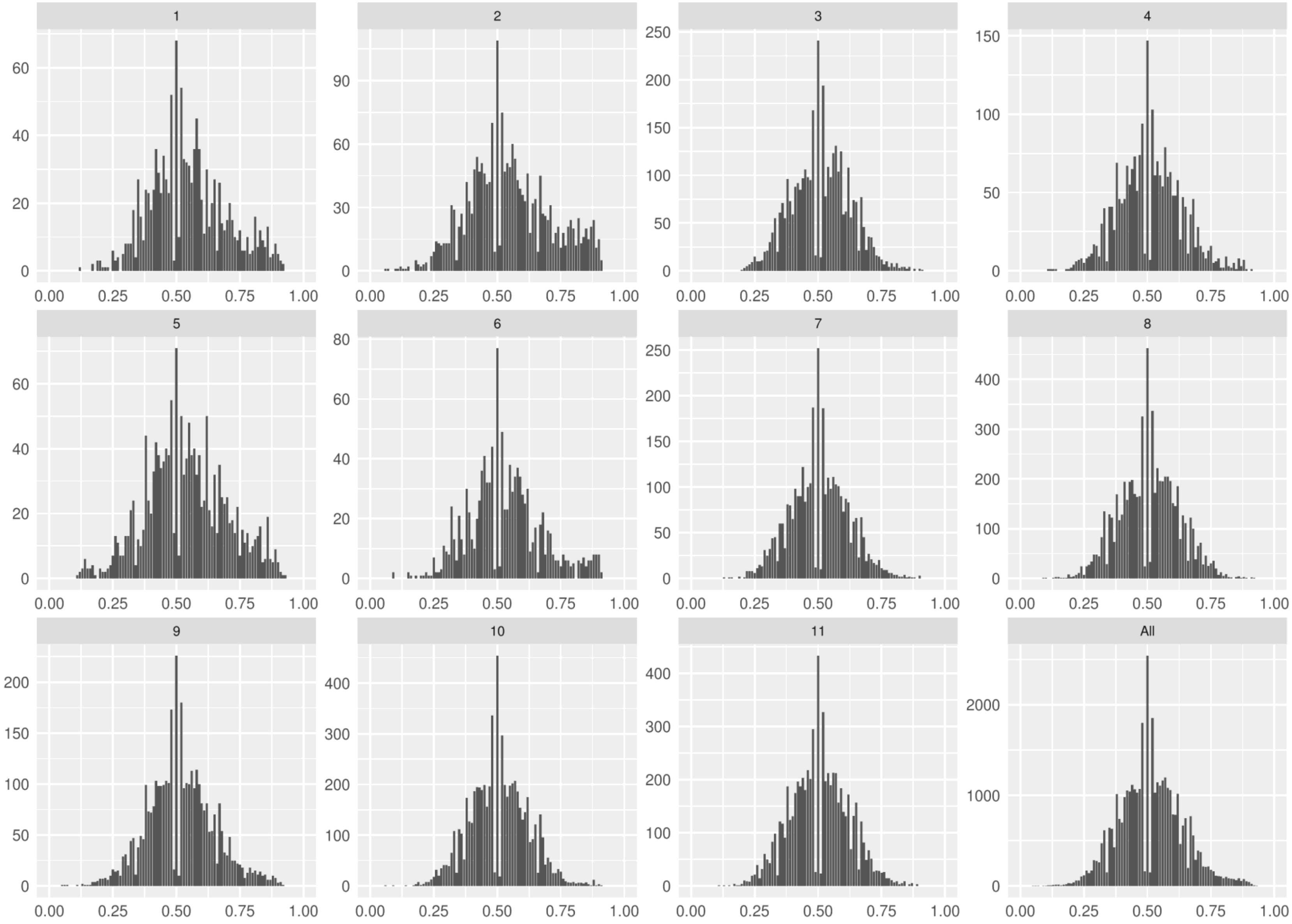

T. b. rhodesiense - Isolate LWO011A

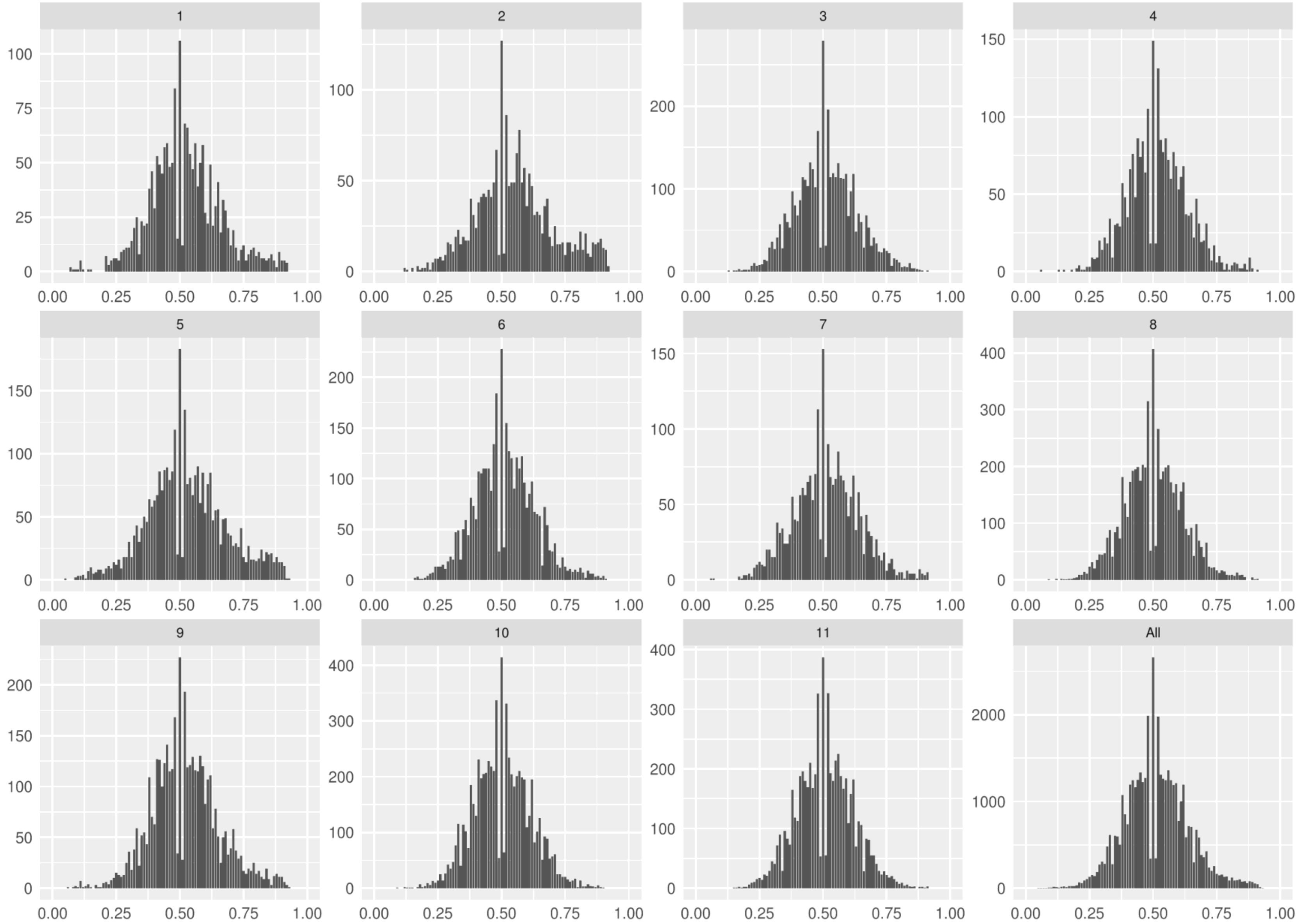

T. b. brucei **- Isolate STIB920**

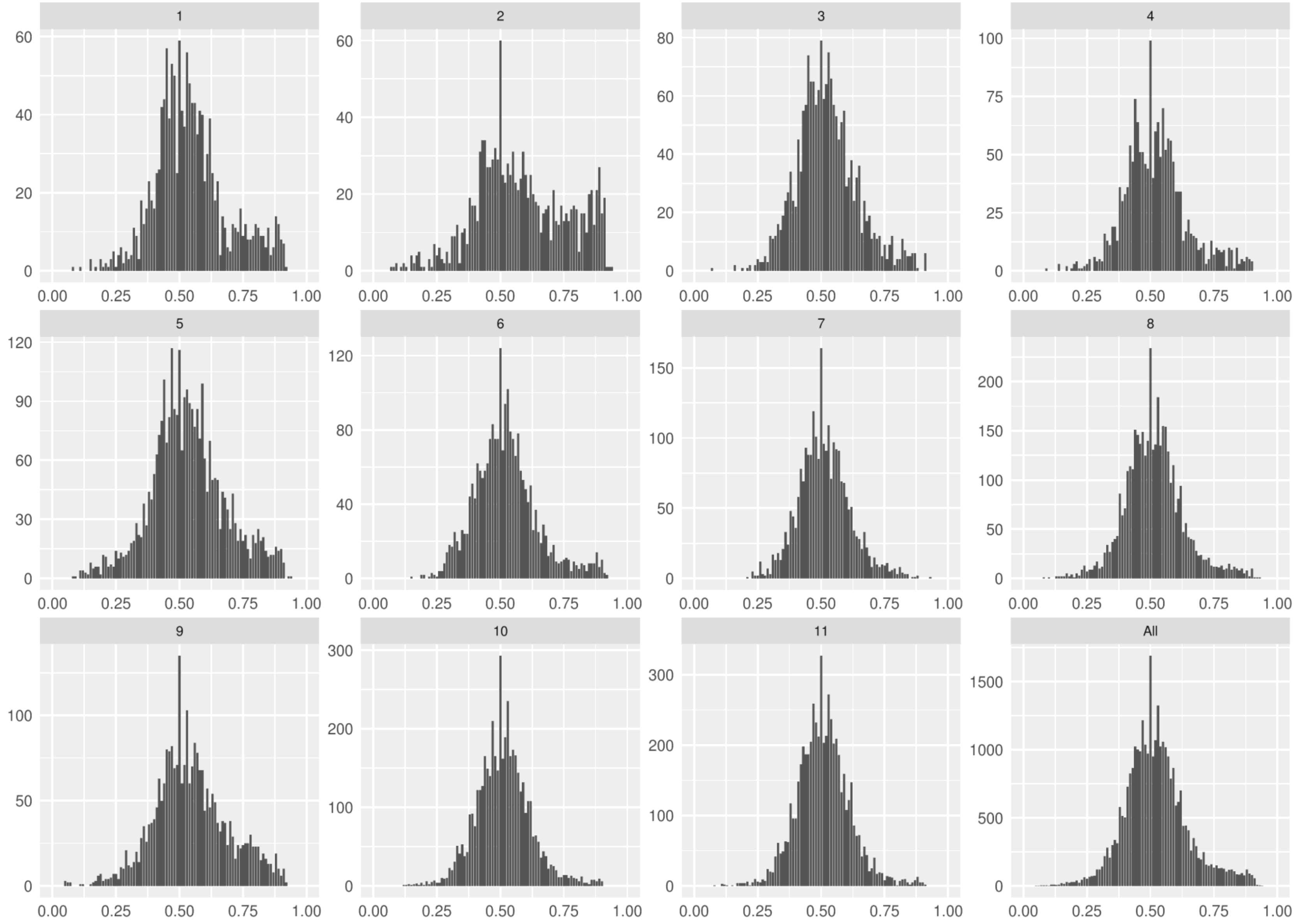

T. b. brucei **- Isolate STIB348TBABB**

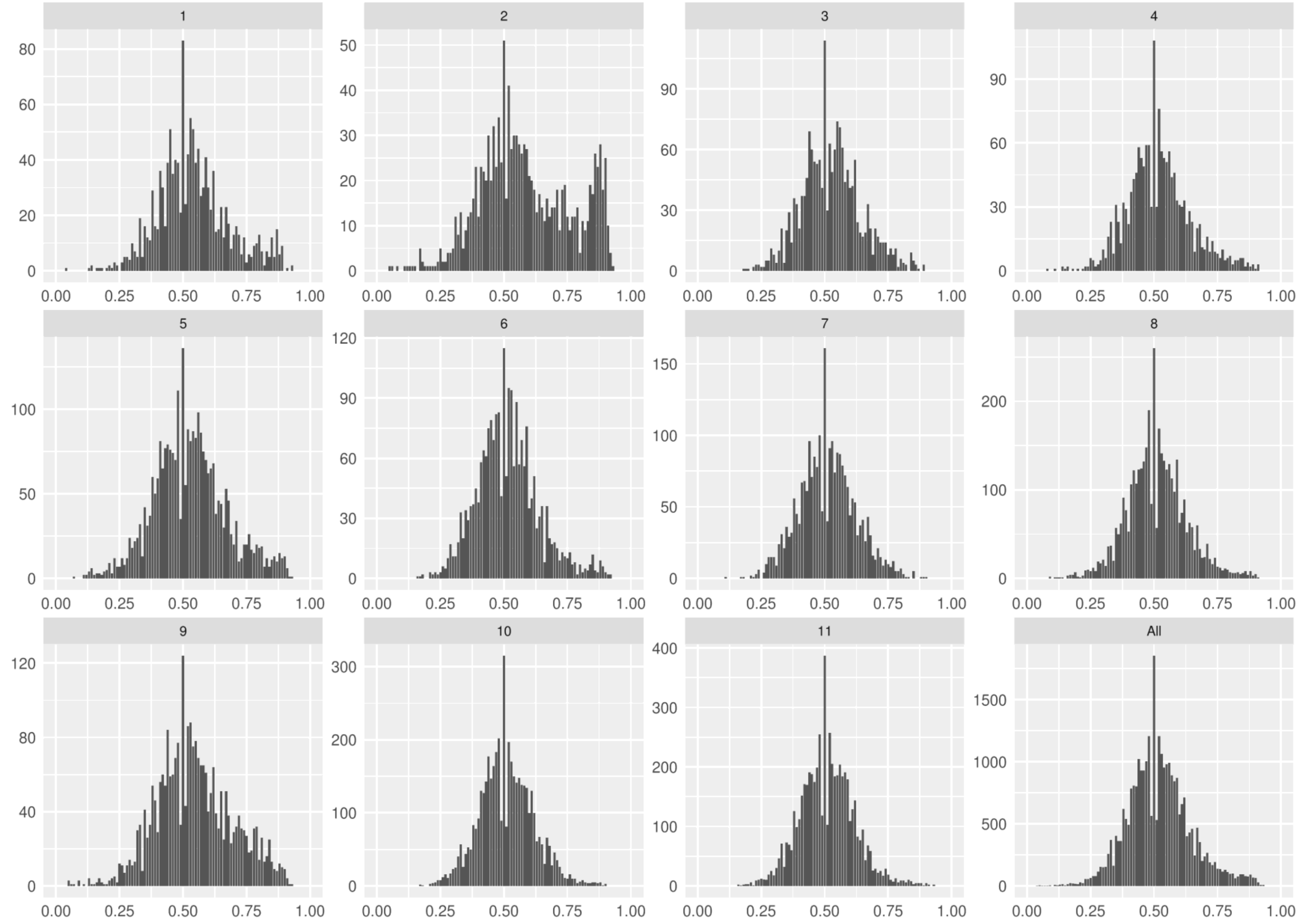

T. b. brucei **– Isolate Cow248**

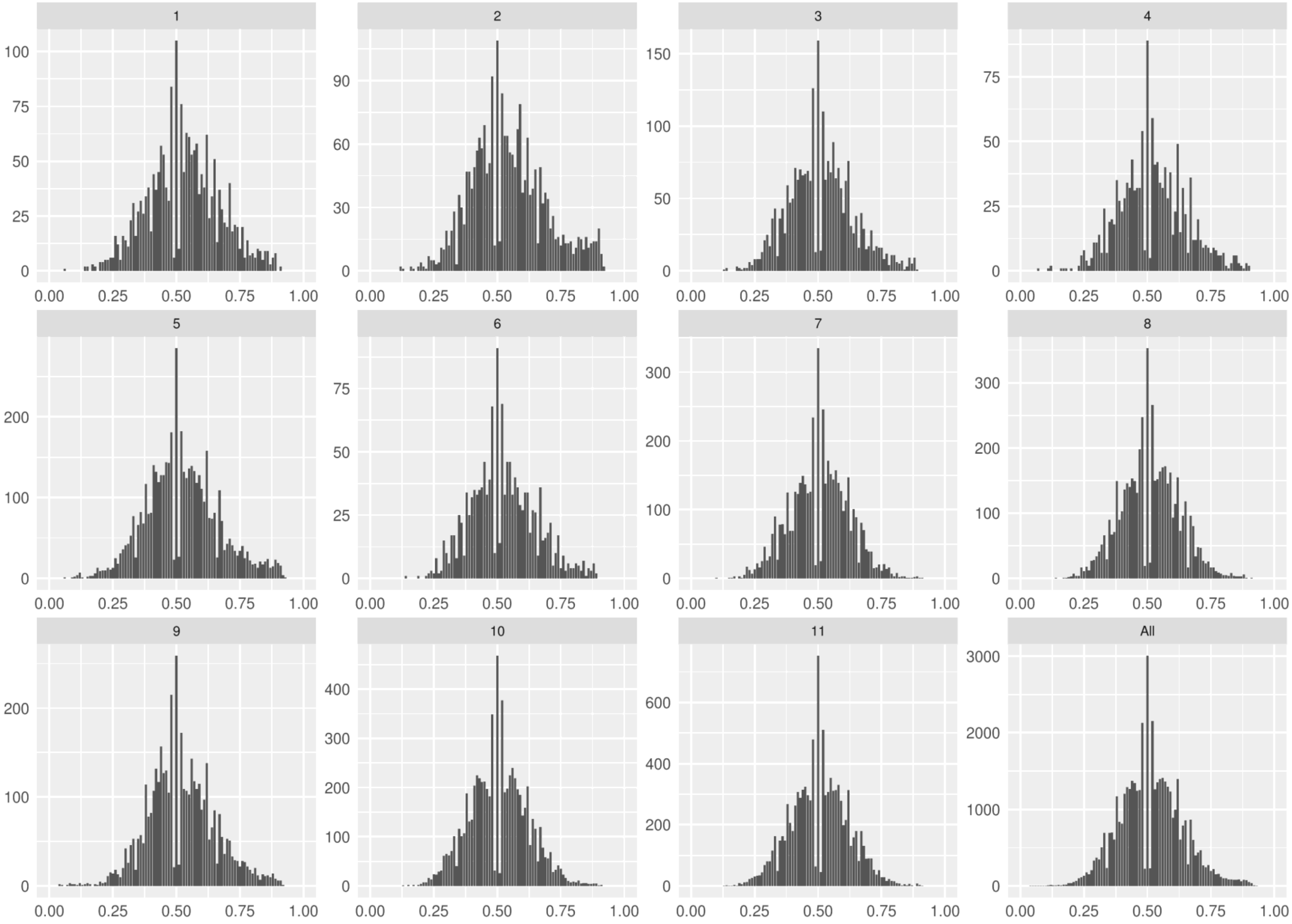

***T. b. brucei* – Isolate 503**

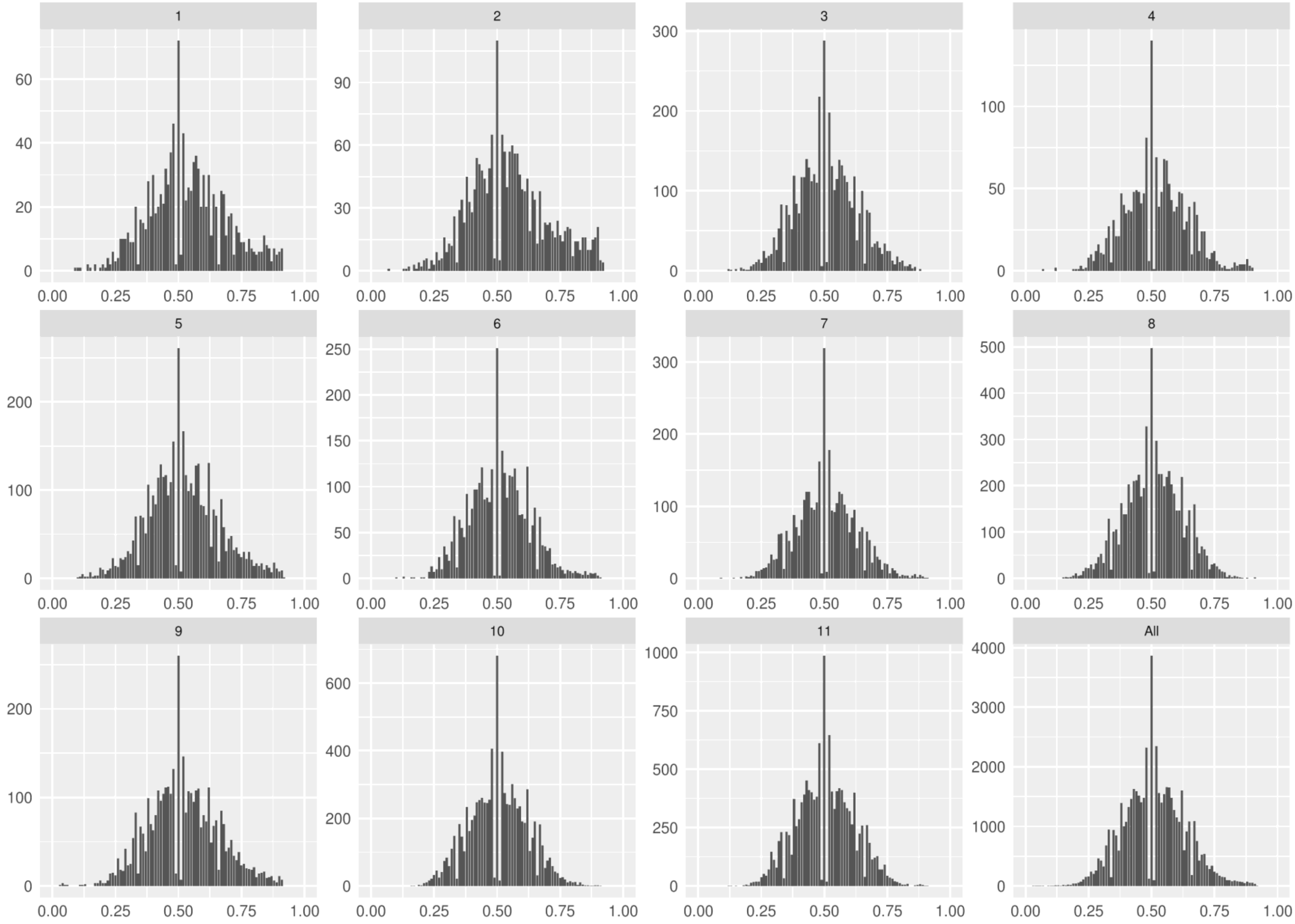

***T. b. gambiense* – Isolate I5**

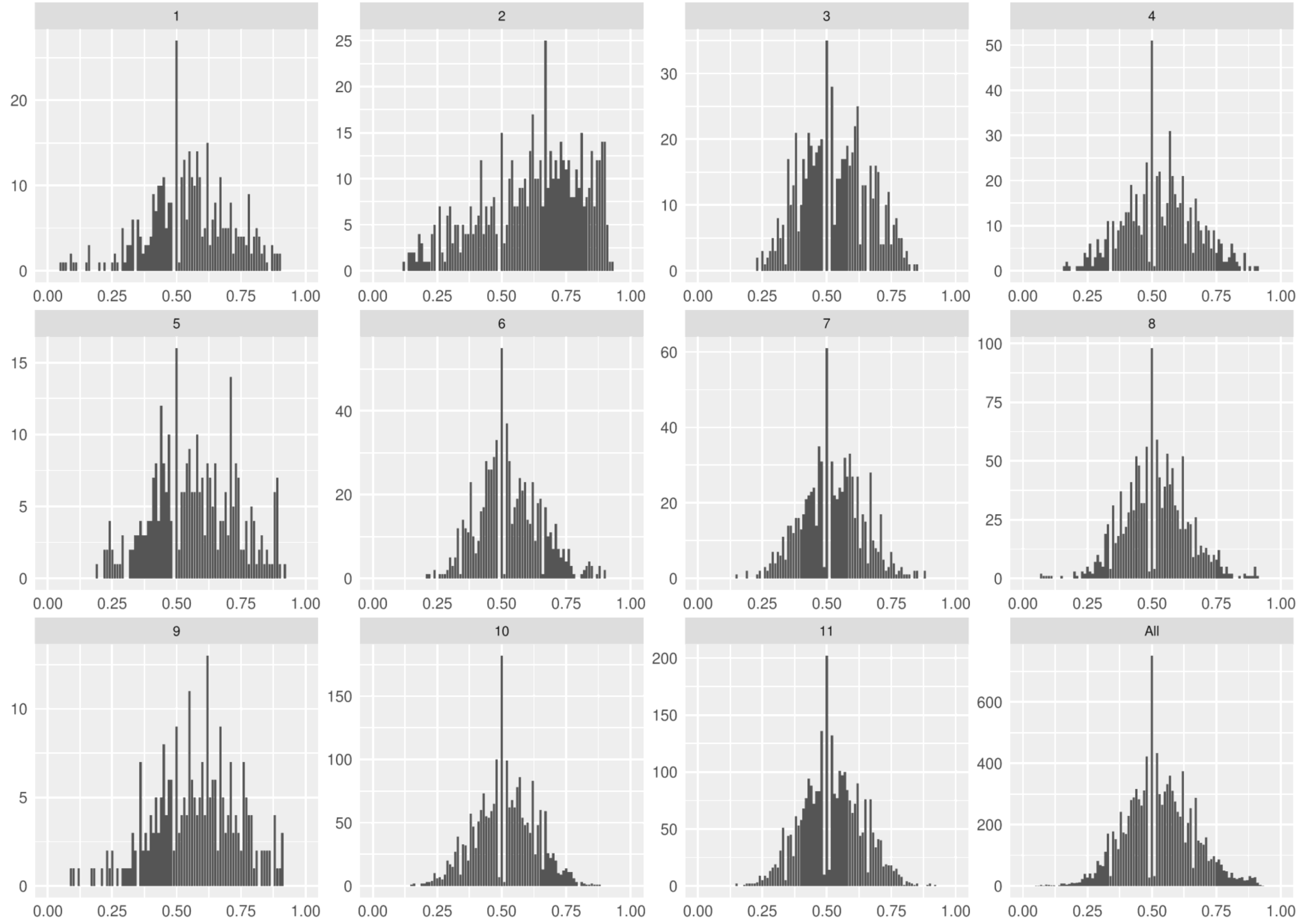

T. b. gambiense – Isolate I7

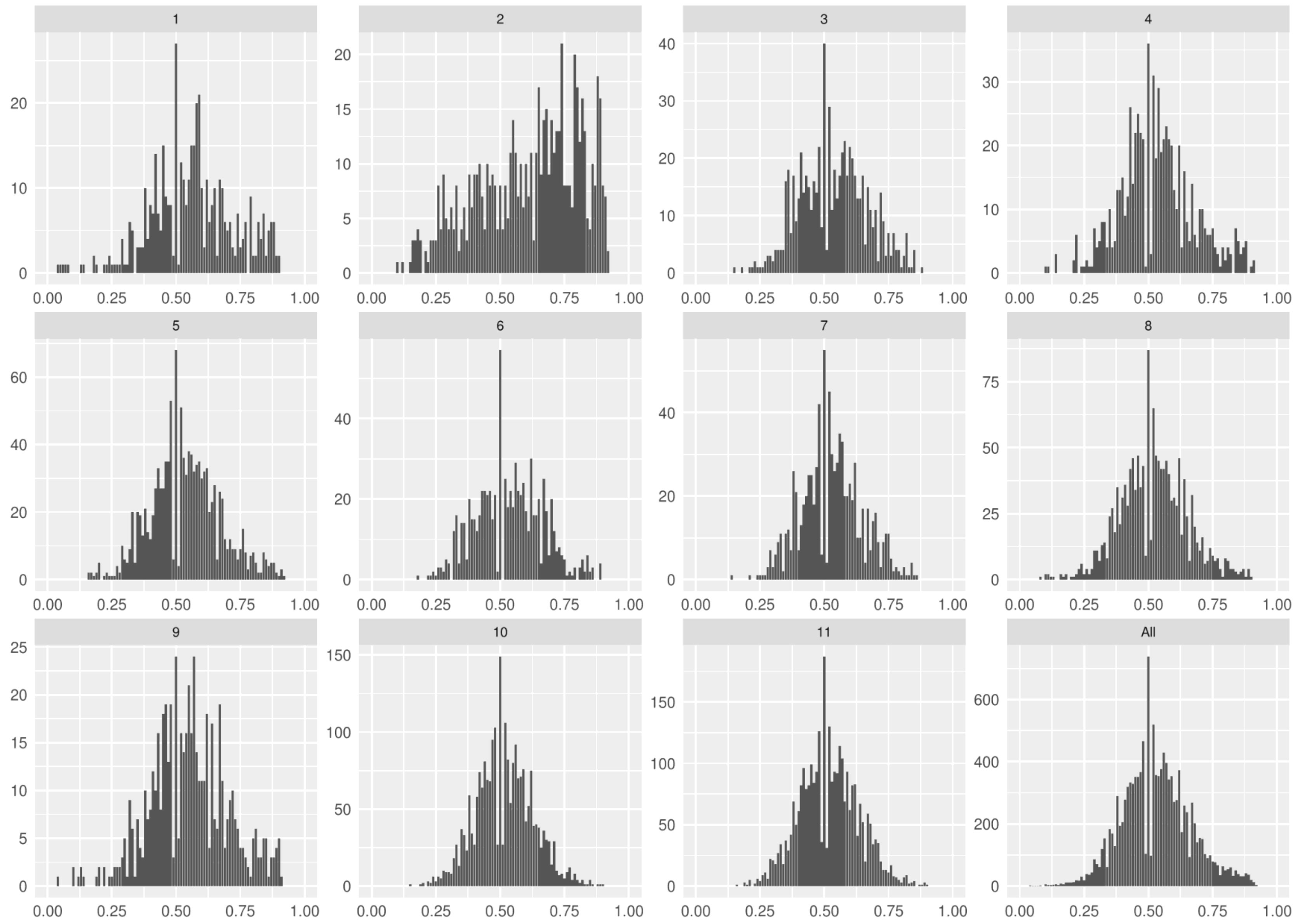

Supplementary Figure 4: RDC variation along *T. brucei* chromosomes. The blue line corresponds to the normalized RDC of each position, estimated by the ratio between the RDC and the genome coverage. Below, the protein-coding genes are depicted as rectangles drawn as proportional to their length, and their coding strand is indicated by their position above (top strand) or below (bottom strand) the central line. Cyan boxes represent VSGs and ESAGs. Black rectangles represent all other genes. Gaps are represented by gene-less regions with no read coverage. The possible regions of segmental duplication are highlighted by red boxes.

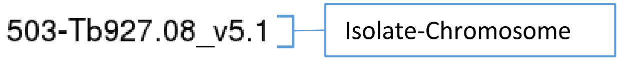

Example:

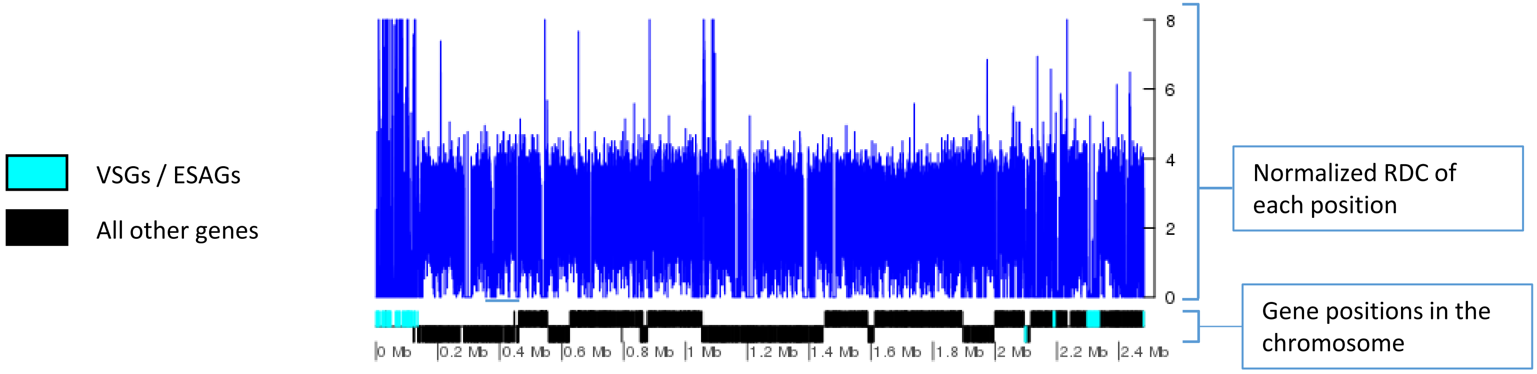

Isolate
503

503-Tb927.01_v5.1

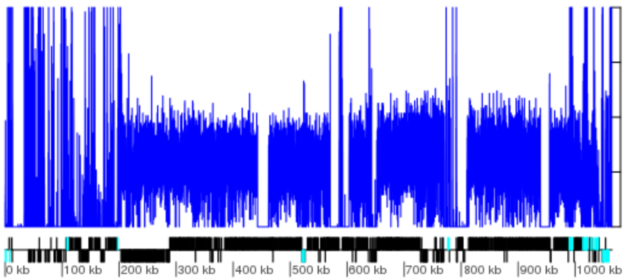

503-Tb927.02_v5.1

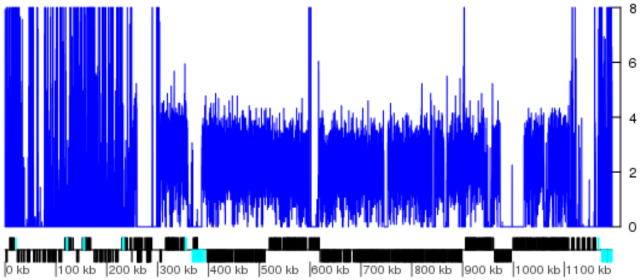

503-Tb927.03_v5.1

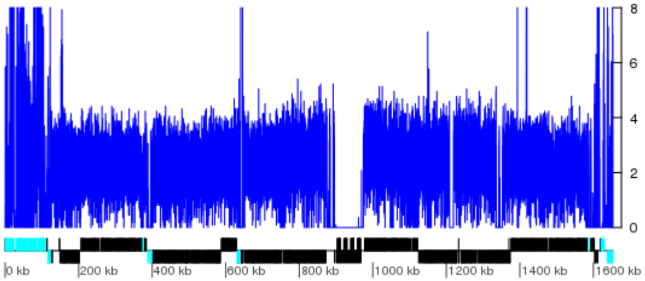

503-Tb927.04_v5.1

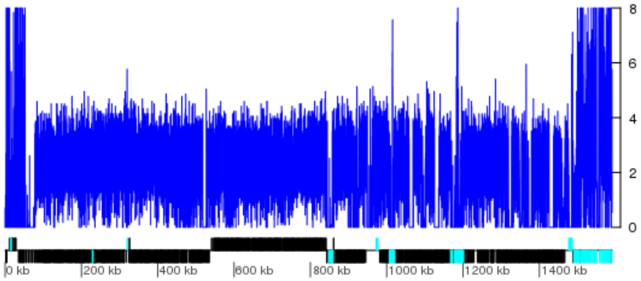

503-Tb927.05_v5.1

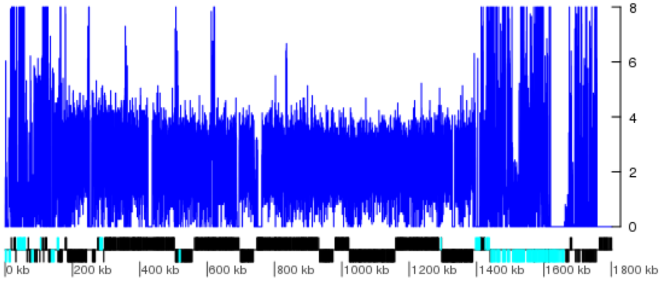

503-Tb927.06_v5.1

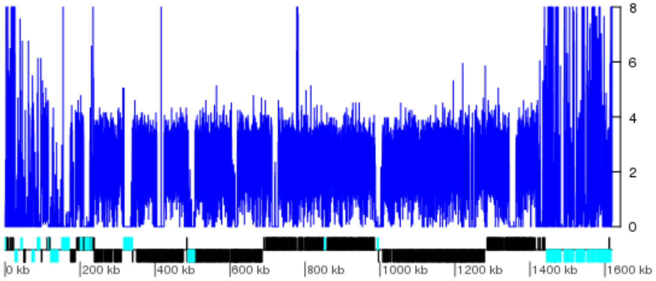

503-Tb927.07_v5.1

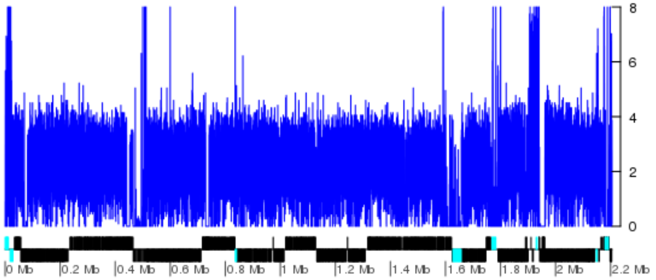

503-Tb927.08_v5.1

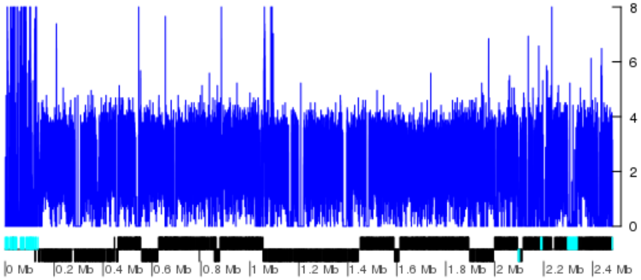

503-Tb927.09_v5.1

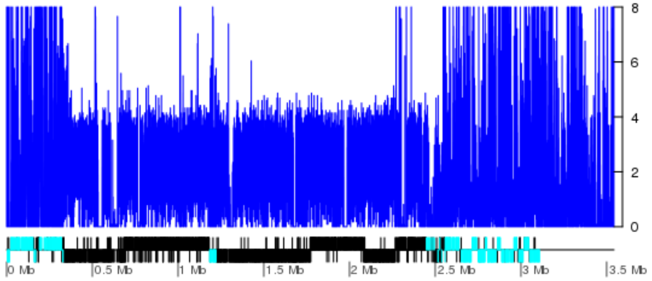

503-Tb927.10_v5.1

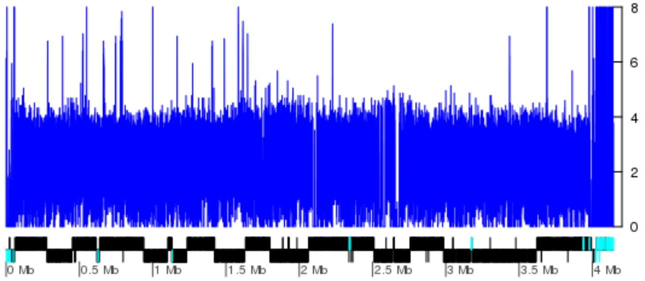

503-Tb927.11_v5.1

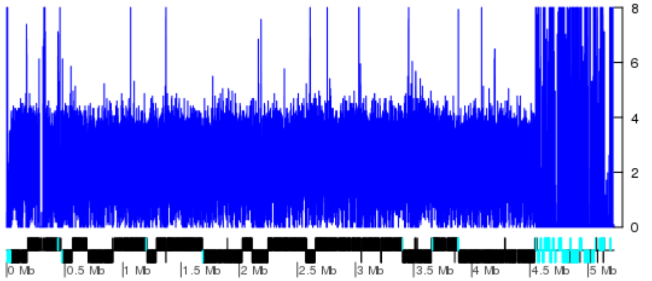

Isolate
Angwen

Angwen-Tb927.01_v5.1

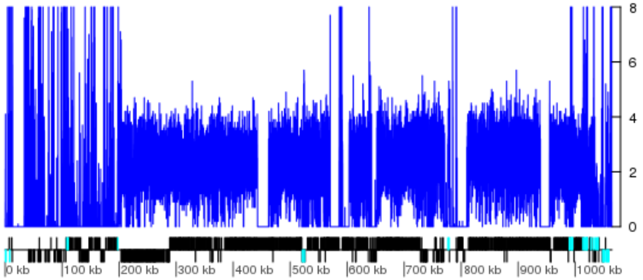

Angwen-Tb927.02_v5.1

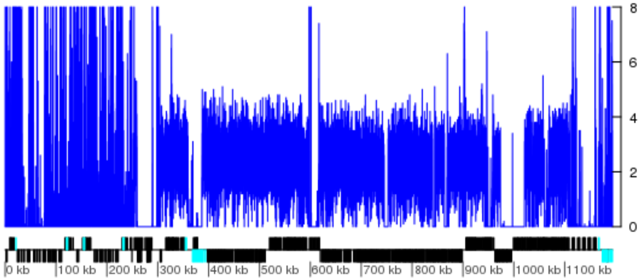

Angwen-Tb927.03_v5.1

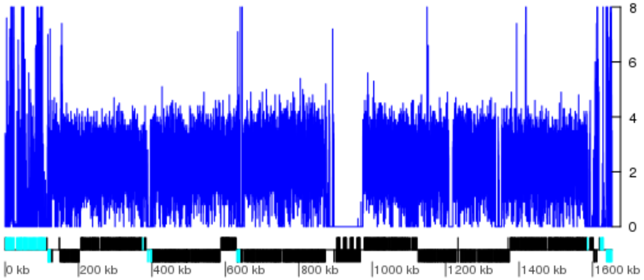

Angwen-Tb927.04_v5.1

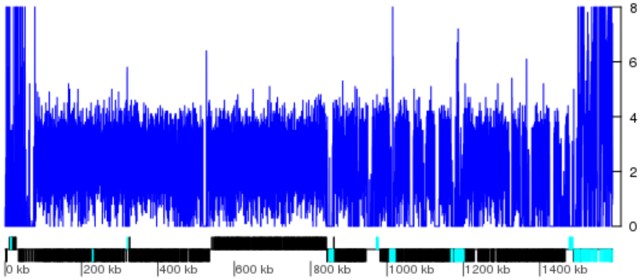

Angwen-Tb927.05_v5.1

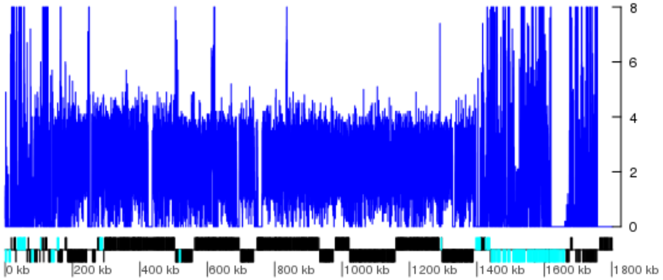

Angwen-Tb927.06_v5.1

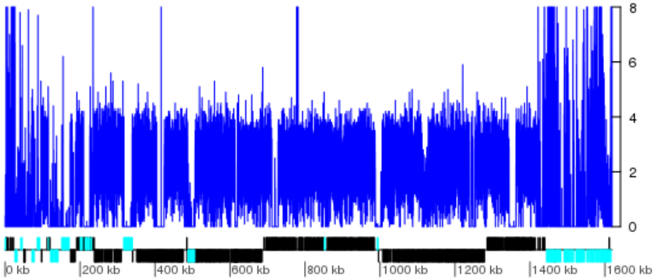

Angwen-Tb927.07_v5.1

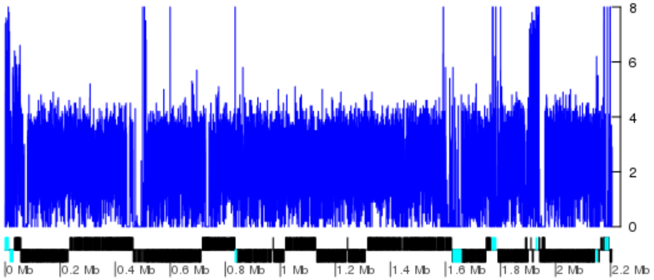

Angwen-Tb927.08_v5.1

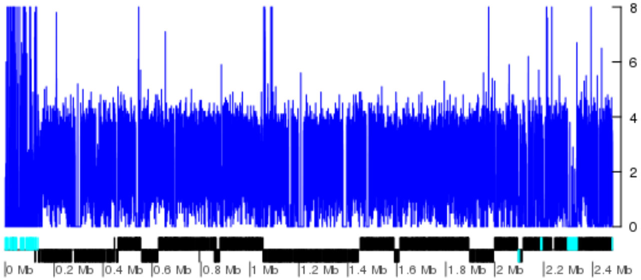

Angwen-Tb927.09_v5.1

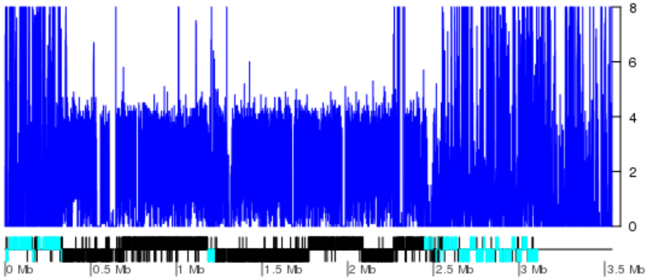

Angwen-Tb927.10_v5.1

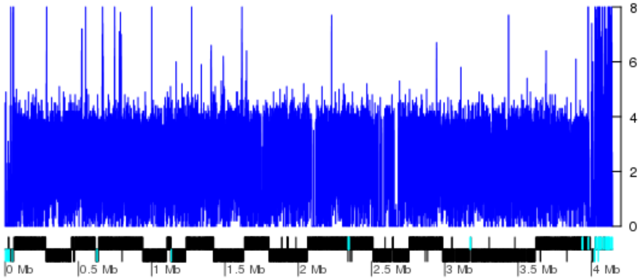

Angwen-Tb927.11_v5.1

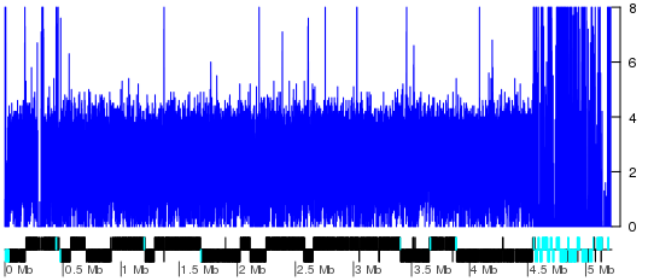

Isolate cow428

cow428-Tb927.01_v5.1

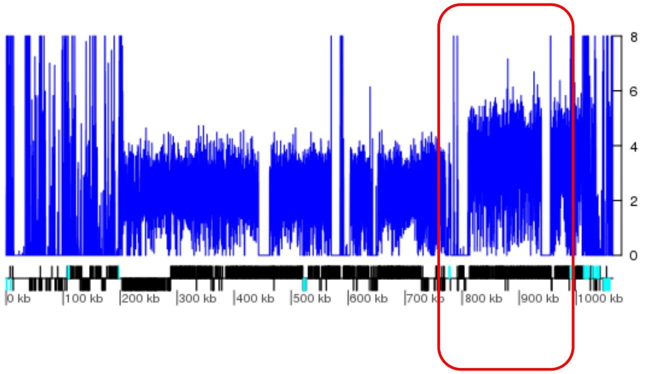

cow428-Tb927.02_v5.1

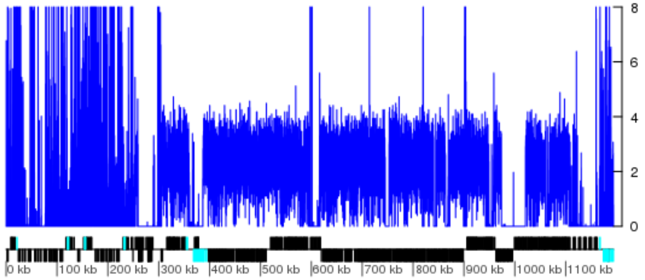

cow428-Tb927.03_v5.1

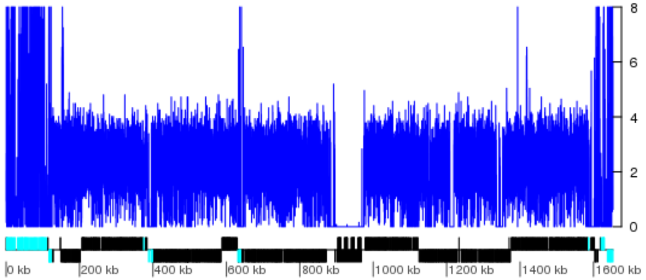

cow428-Tb927.04_v5.1

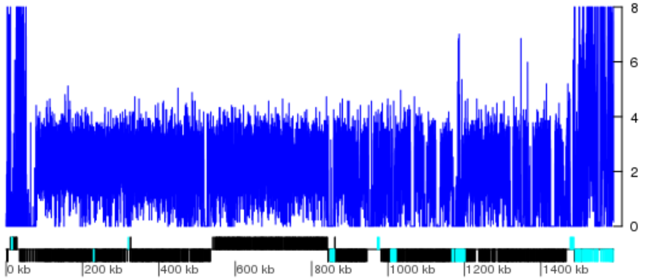

cow428-Tb927.05_v5.1

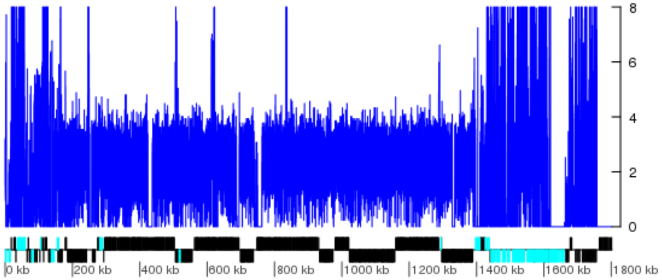

cow428-Tb927.06_v5.1

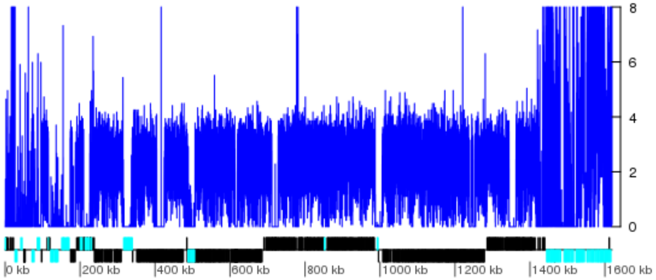

cow428-Tb927.07_v5.1

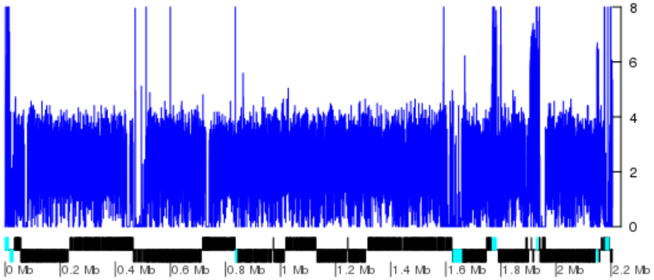

cow428-Tb927.08_v5.1

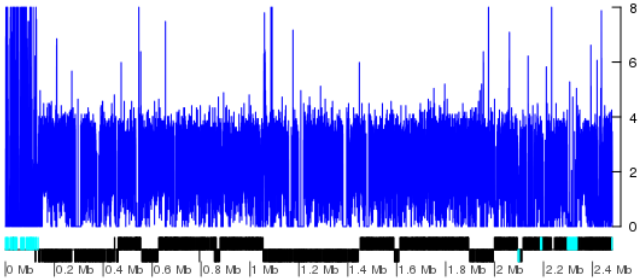

cow428-Tb927.09_v5.1

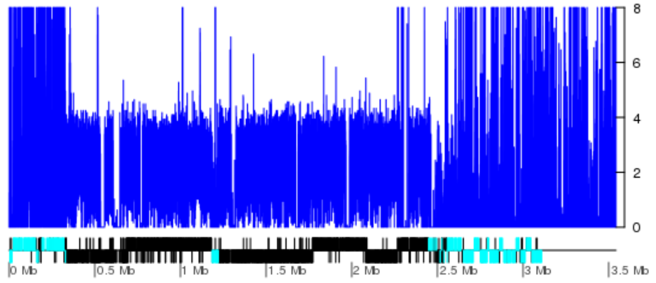

cow428-Tb927.10_v5.1

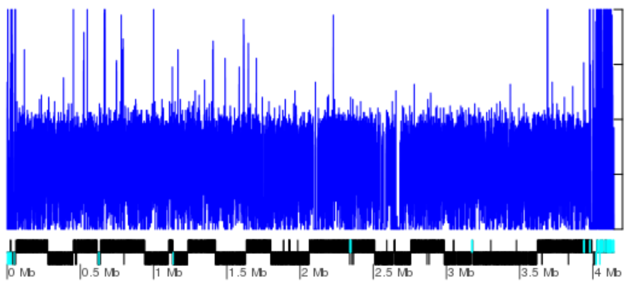

cow428-Tb927.11_v5.1

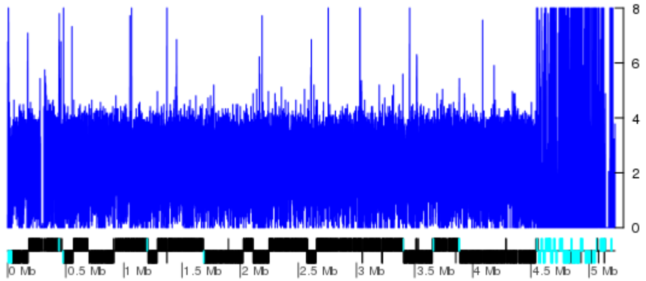

Isolate
D1

D1-Tb927.01_v5.1

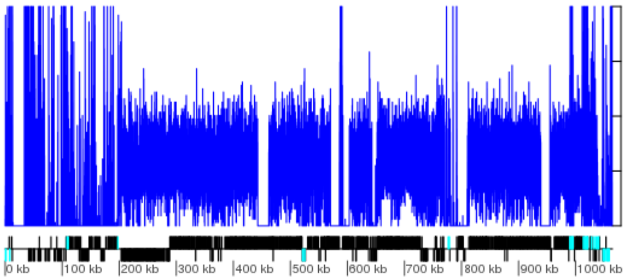

D1-Tb927.02_v5.1

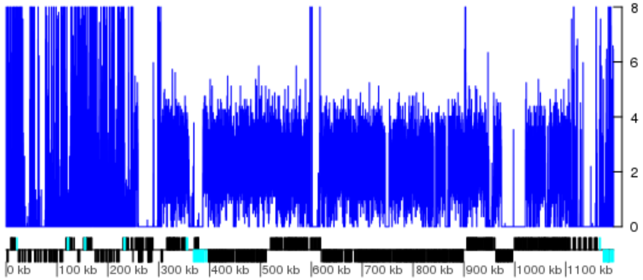

D1-Tb927.03_v5.1

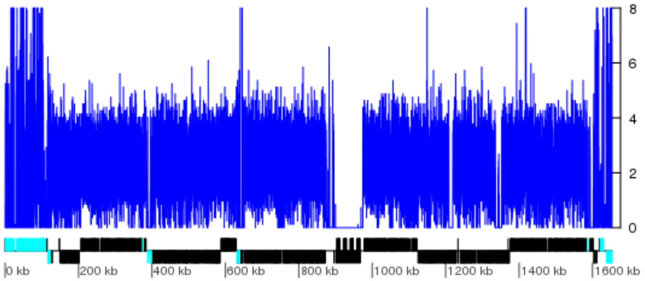

D1-Tb927.04_v5.1

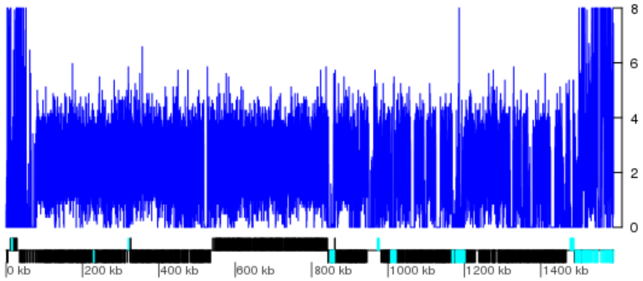

D1-Tb927.05_v5.1

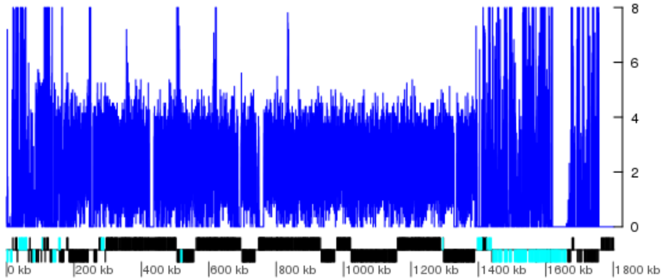

D1-Tb927.06_v5.1

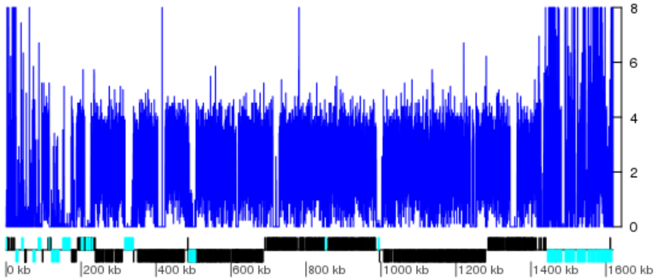

D1-Tb927.07_v5.1

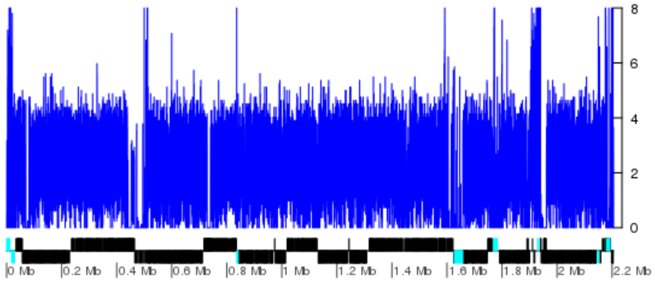

D1-Tb927.08_v5.1

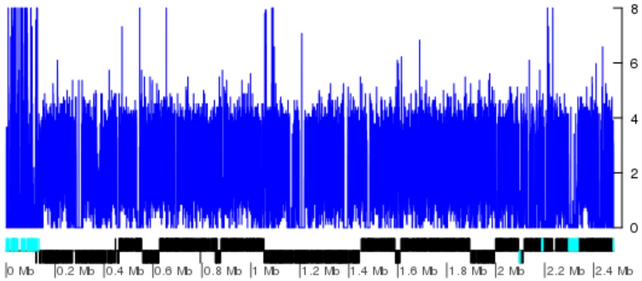

D1-Tb927.09_v5.1

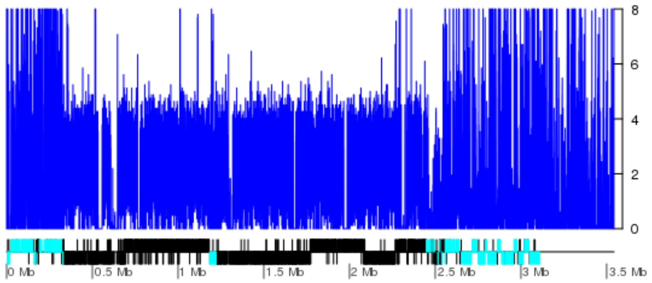

D1-Tb927.10_v5.1

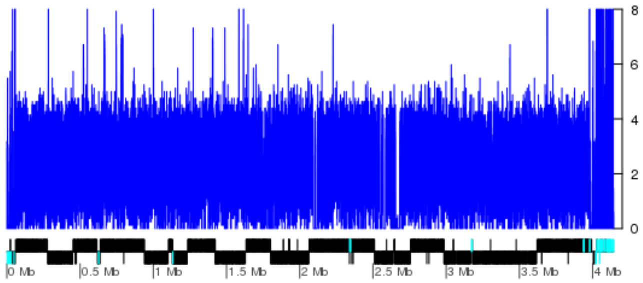

D1-Tb927.11_v5.1

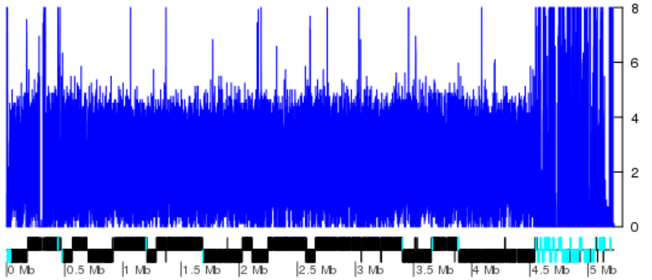

Isolate
D2

D2-Tb927.01_v5.1

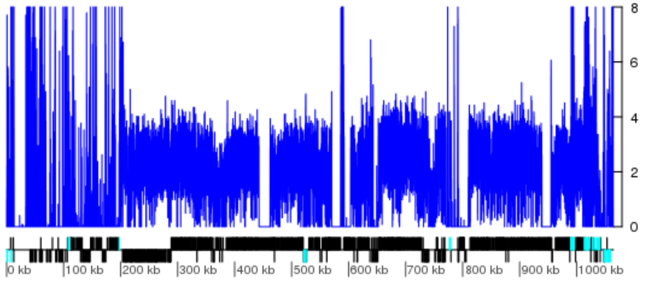

D2-Tb927.02_v5.1

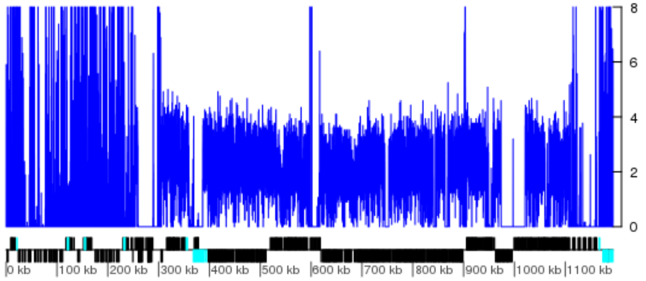

D2-Tb927.03_v5.1

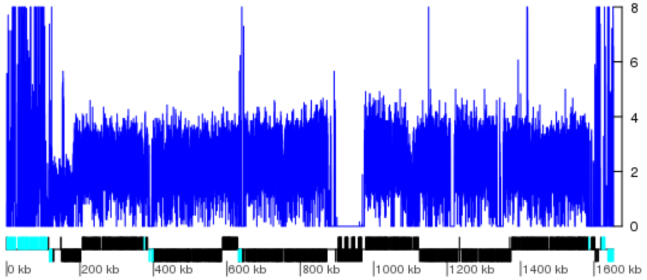

D2-Tb927.04_v5.1

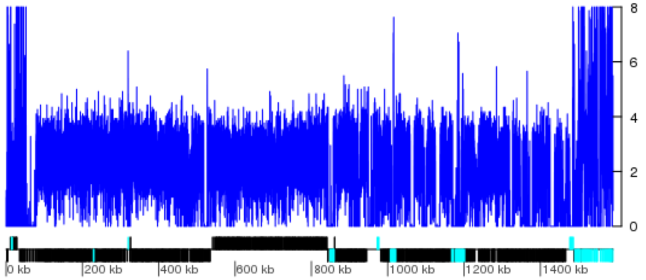

D2-Tb927.05_v5.1

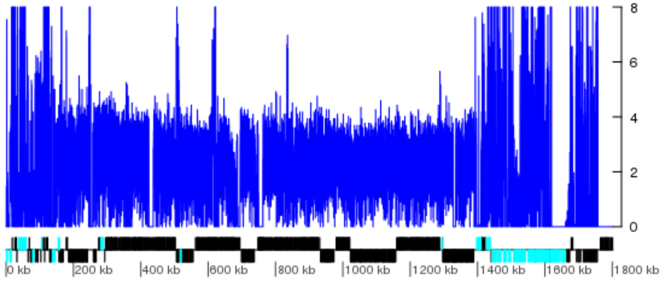

D2-Tb927.06_v5.1

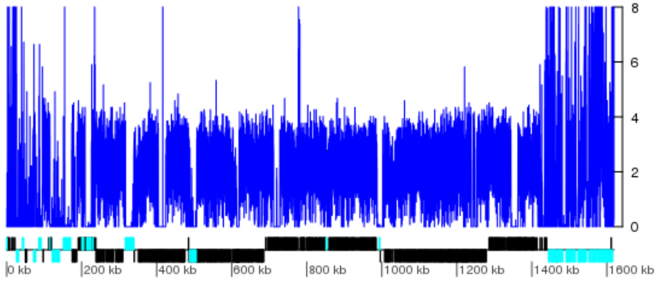

D2-Tb927.07_v5.1

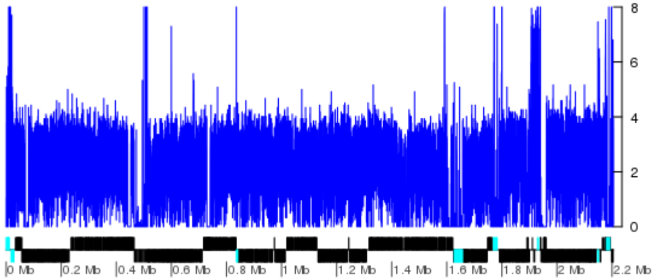

D2-Tb927.08_v5.1

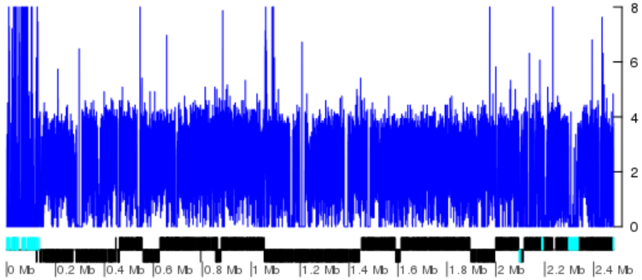

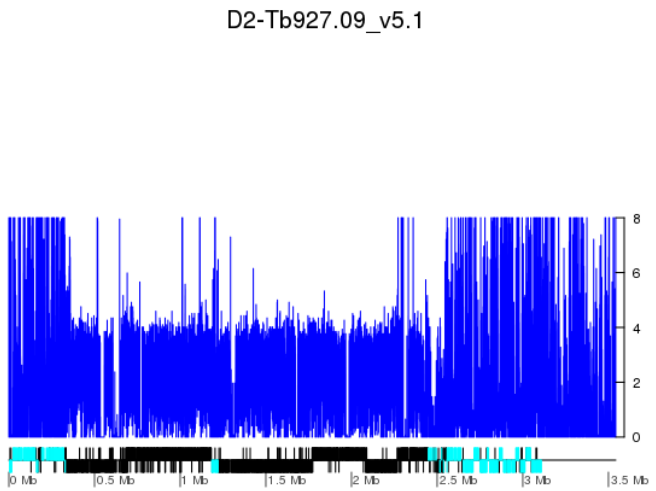
D2-Tb927.09_v5.1
8
6
4
2
0
0 Mb
0.5 Mb
1 Mb
1.5 Mb
2 Mb
2.5 Mb
3 Mb
3.5 Mb

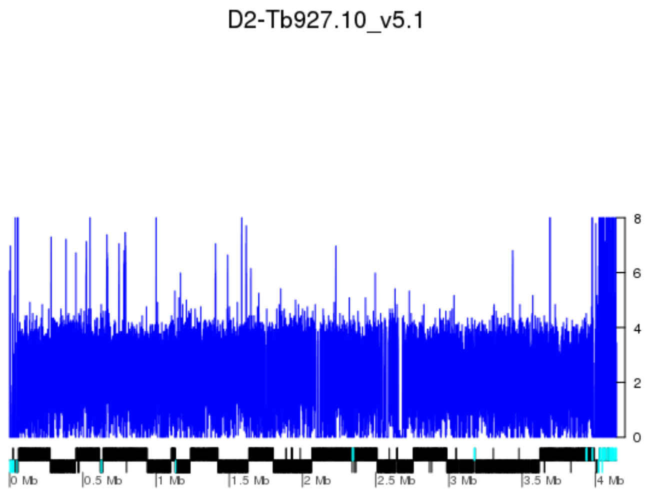
D2-Tb927.10_v5.1
8
6
4
2
0
0 Mb
0.5 Mb
1 Mb
1.5 Mb
2 Mb
2.5 Mb
3 Mb
3.5 Mb
4 Mb

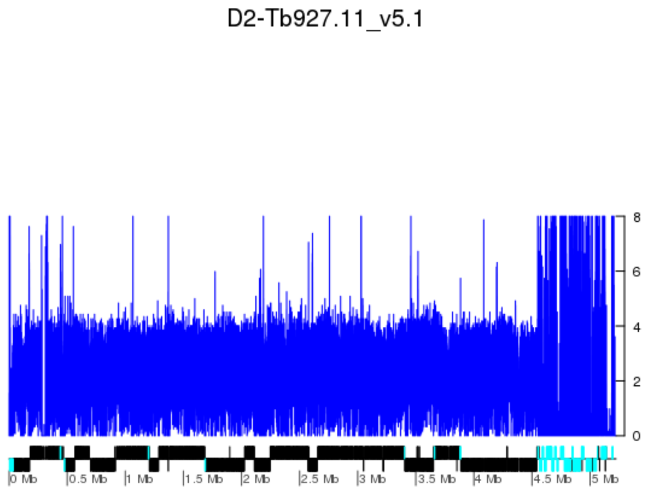
D2-Tb927.11_v5.1
8
6
4
2
0
0 Mb
0.5 Mb
1 Mb
1.5 Mb
2 Mb
2.5 Mb
3 Mb
3.5 Mb
4 Mb
4.5 Mb
5 Mb

Isolate
D3

D3-Tb927.01_v5.1

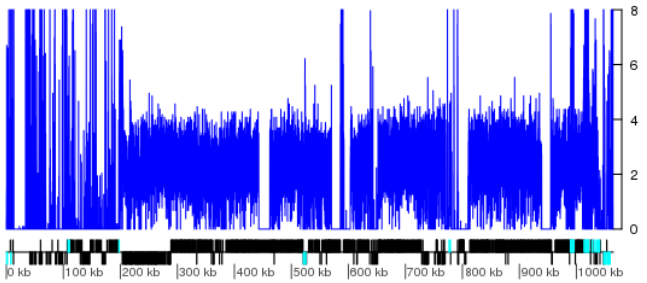

D3-Tb927.02_v5.1

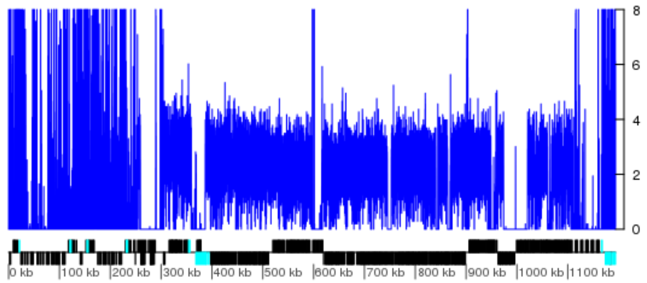

D3-Tb927.03_v5.1

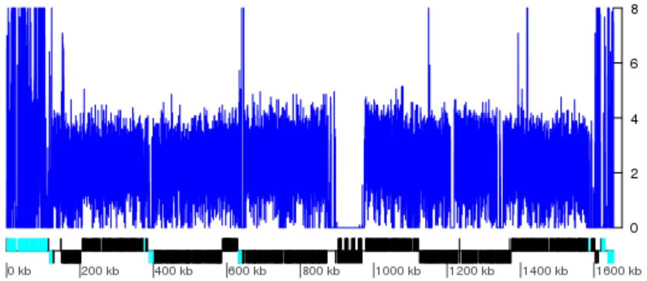

D3-Tb927.04_v5.1

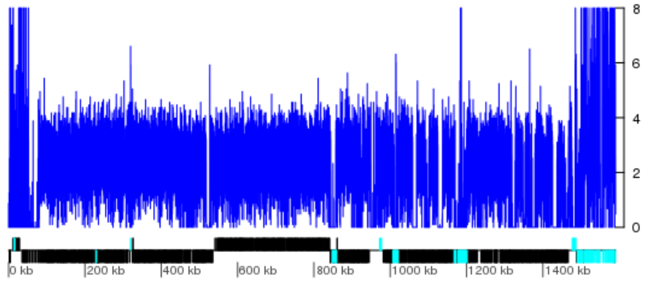

D3-Tb927.05_v5.1

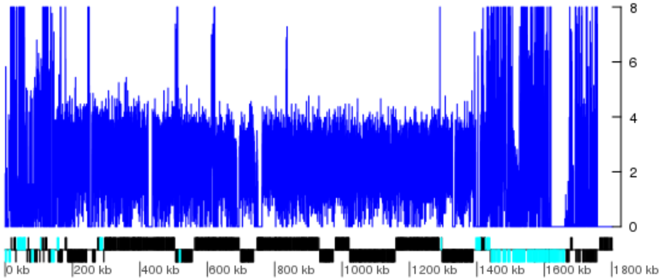

D3-Tb927.06_v5.1

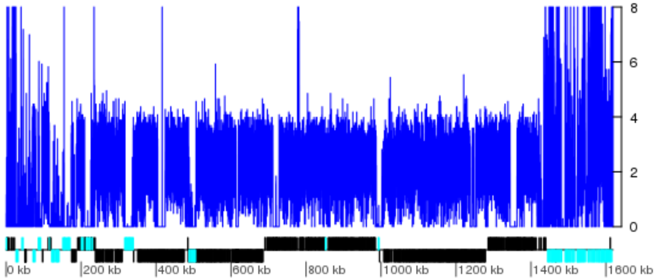

D3-Tb927.07_v5.1

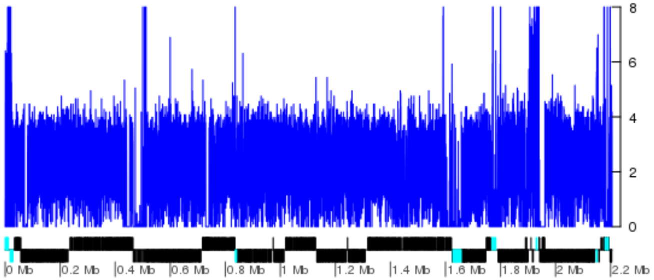

D3-Tb927.08_v5.1

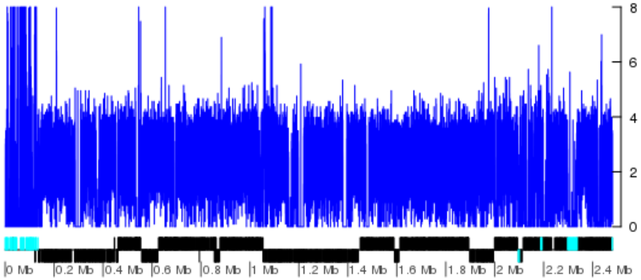

D3-Tb927.09_v5.1

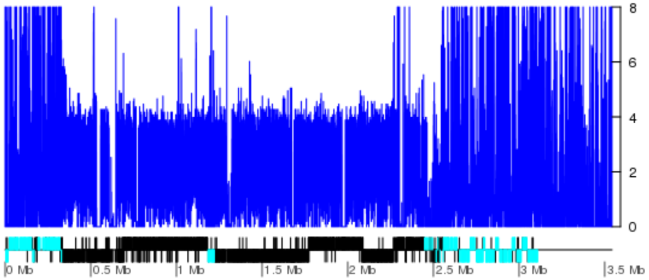

D3-Tb927.10_v5.1

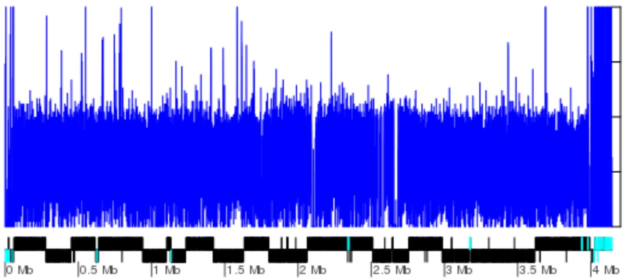

D3-Tb927.11_v5.1

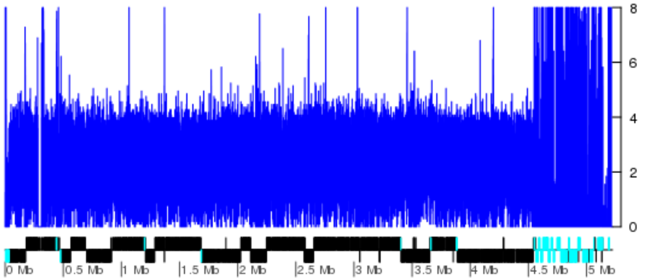

Isolate
D4

D4-Tb927.01_v5.1

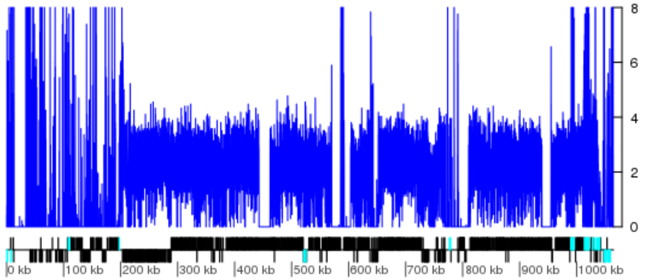

D4-Tb927.02_v5.1

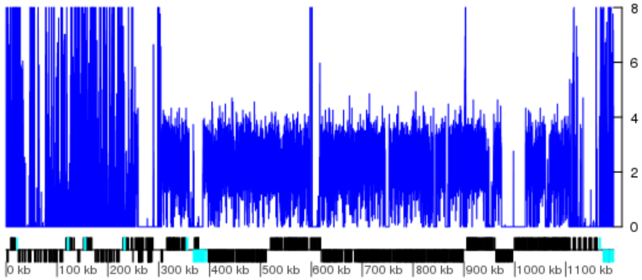

D4-Tb927.03_v5.1

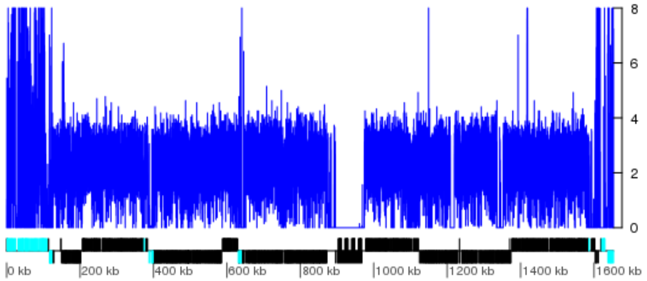

D4-Tb927.04_v5.1

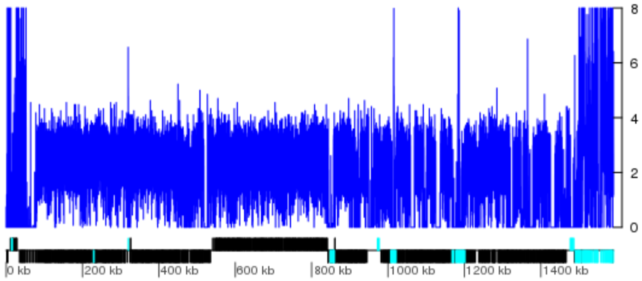

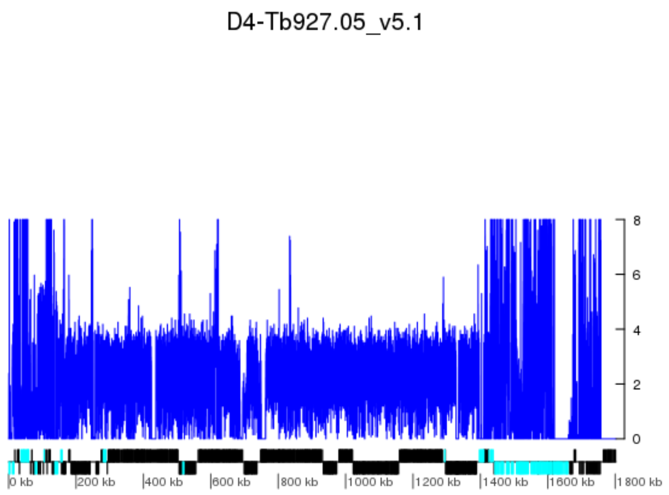
D4-Tb927.05_v5.1
8
6
4
2
0
0 kb
200 kb
400 kb
600 kb
800 kb
1000 kb
1200 kb
1400 kb
1600 kb
1800 kb

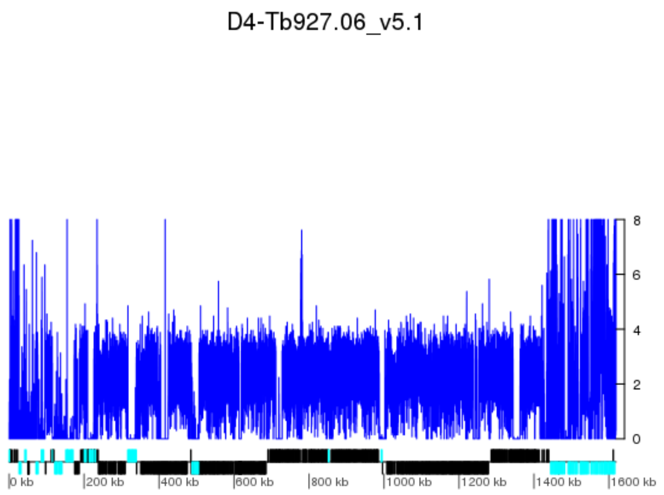
D4-Tb927.06_v5.1
8
6
4
2
0
0 kb
200 kb
400 kb
600 kb
800 kb
1000 kb
1200 kb
1400 kb
1600 kb

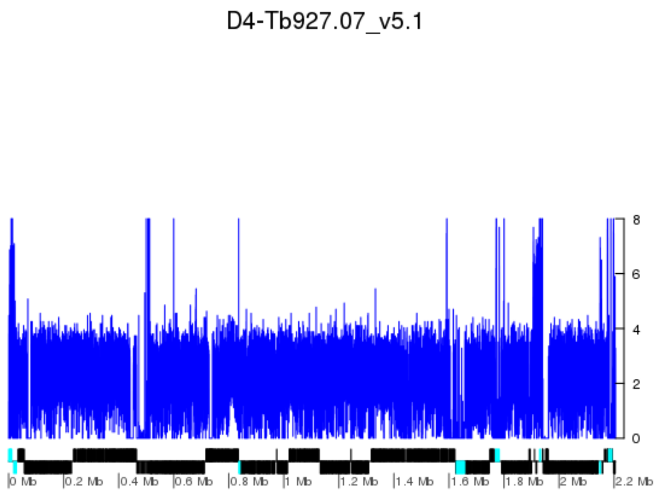
D4-Tb927.07_v5.1
8
6
4
2
0
0 Mb
0.2 Mb
0.4 Mb
0.6 Mb
0.8 Mb
1 Mb
1.2 Mb
1.4 Mb
1.6 Mb
1.8 Mb
2 Mb
2.2 Mb

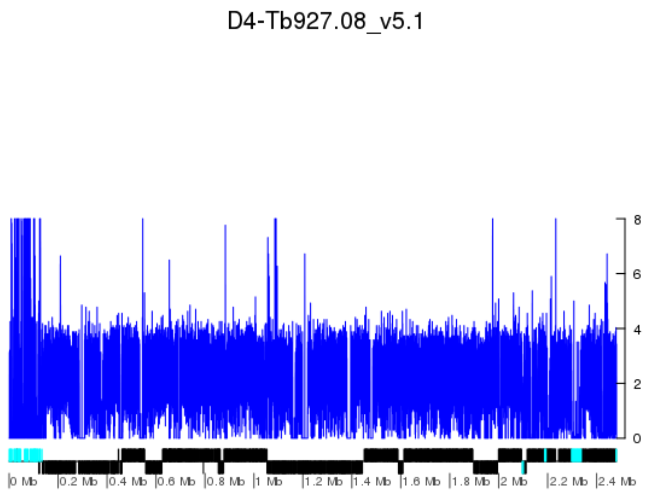
D4-Tb927.08_v5.1
8
6
4
2
0
0 Mb
0.2 Mb
0.4 Mb
0.6 Mb
0.8 Mb
1 Mb
1.2 Mb
1.4 Mb
1.6 Mb
1.8 Mb
2 Mb
2.2 Mb
2.4 Mb

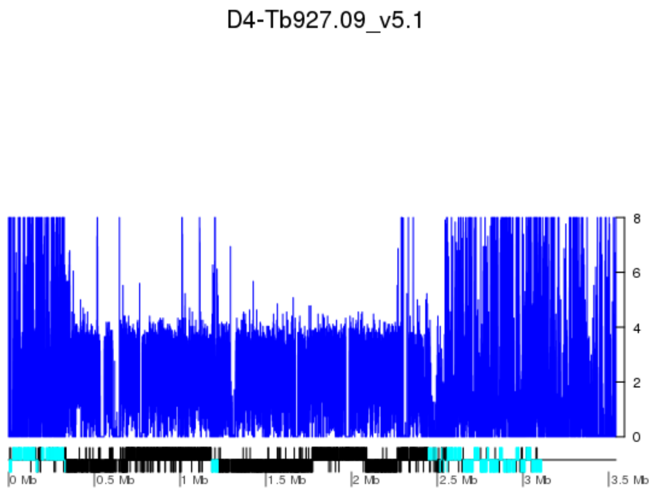
D4-Tb927.09_v5.1
8
6
4
2
0
0 Mb
0.5 Mb
1 Mb
1.5 Mb
2 Mb
2.5 Mb
3 Mb
3.5 Mb

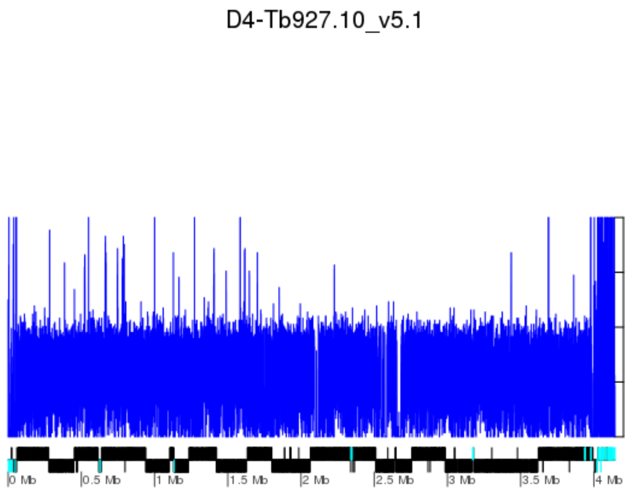
D4-Tb927.10_v5.1
8
6
4
2
0
0 Mb
0.5 Mb
1 Mb
1.5 Mb
2 Mb
2.5 Mb
3 Mb
3.5 Mb
4 Mb

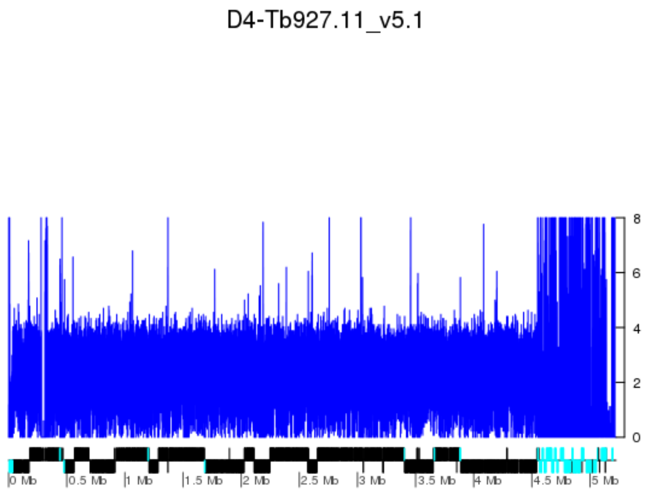
D4-Tb927.11_v5.1
8
6
4
2
0
0 Mb
0.5 Mb
1 Mb
1.5 Mb
2 Mb
2.5 Mb
3 Mb
3.5 Mb
4 Mb
4.5 Mb
5 Mb

Isolate
D5

D5-Tb927.01_v5.1

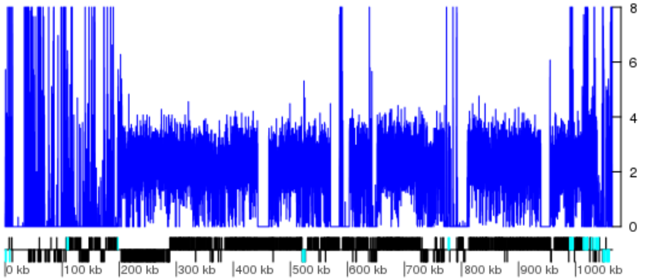

D5-Tb927.02_v5.1

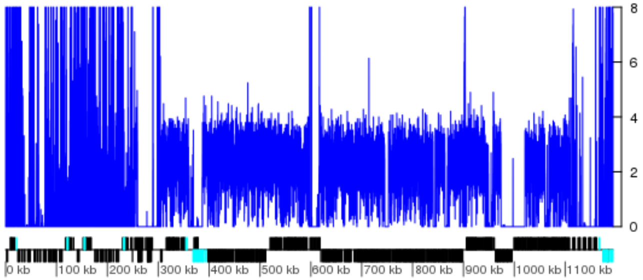

D5-Tb927.03_v5.1

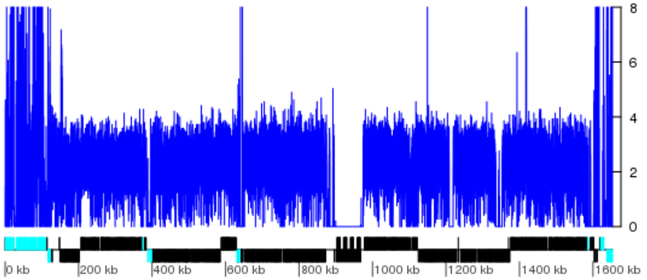

D5-Tb927.04_v5.1

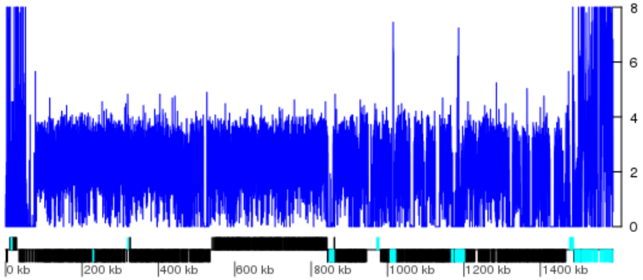

D5-Tb927.05_v5.1

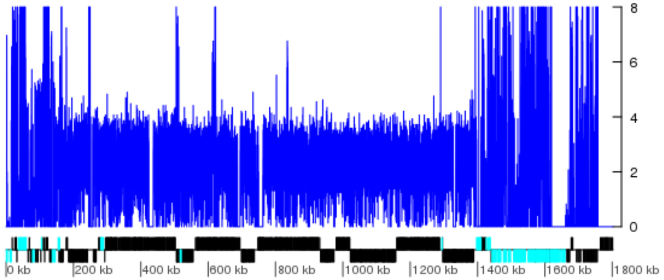

D5-Tb927.06_v5.1

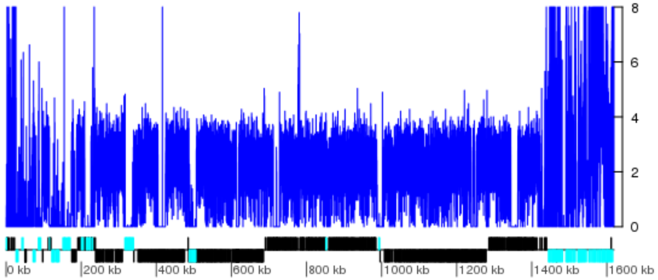

D5-Tb927.07_v5.1

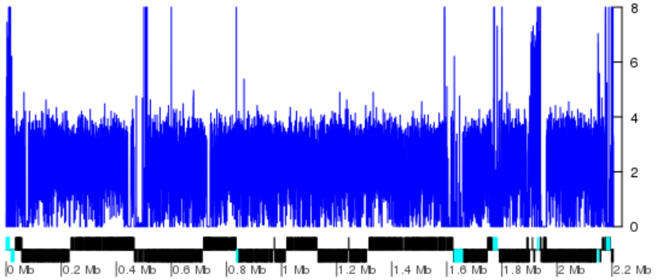

D5-Tb927.08_v5.1

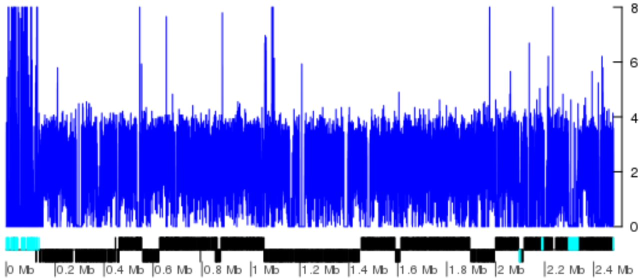

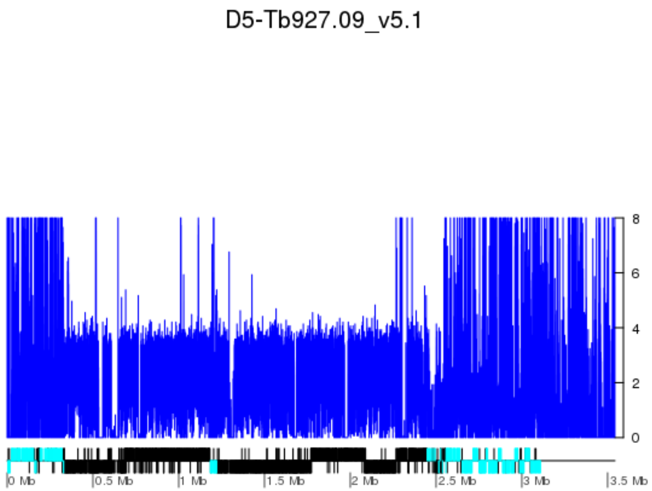
D5-Tb927.09_v5.1
0
2
4
6
8
0 Mb
0.5 Mb
1 Mb
1.5 Mb
2 Mb
2.5 Mb
3 Mb
3.5 Mb

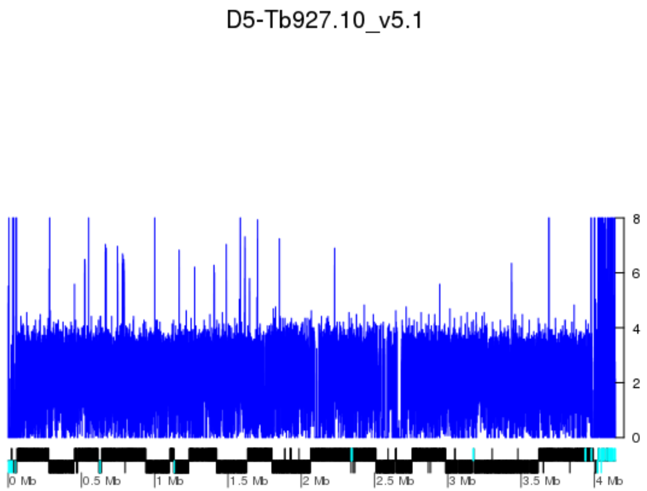
D5-Tb927.10_v5.1
0
2
4
6
8
0 Mb
0.5 Mb
1 Mb
1.5 Mb
2 Mb
2.5 Mb
3 Mb
3.5 Mb
4 Mb

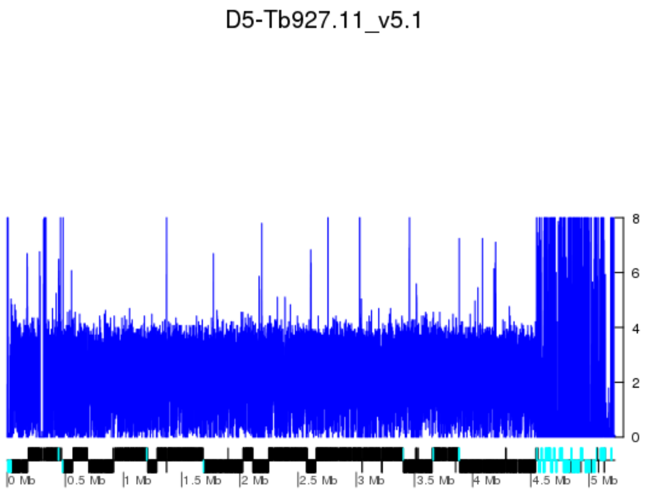
D5-Tb927.11_v5.1
0
2
4
6
8
0 Mb
0.5 Mb
1 Mb
1.5 Mb
2 Mb
2.5 Mb
3 Mb
3.5 Mb
4 Mb
4.5 Mb
5 Mb

Isolate
D7

D7-Tb927.01_v5.1

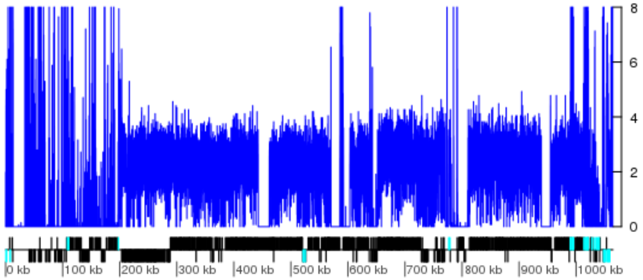

D7-Tb927.02_v5.1

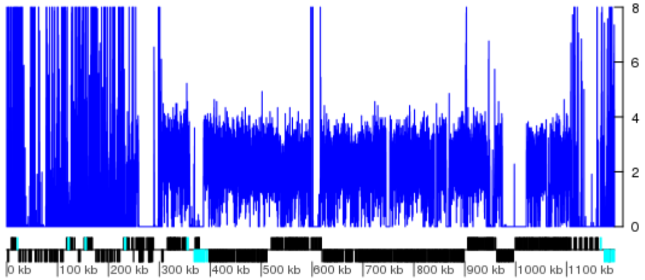

D7-Tb927.03_v5.1

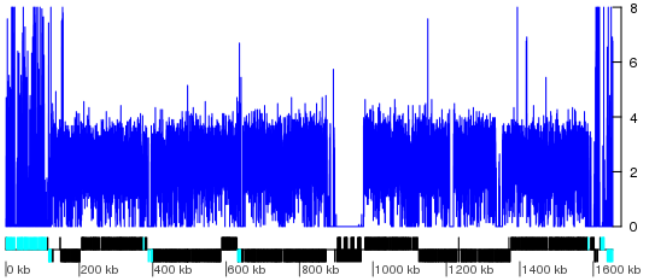

D7-Tb927.04_v5.1

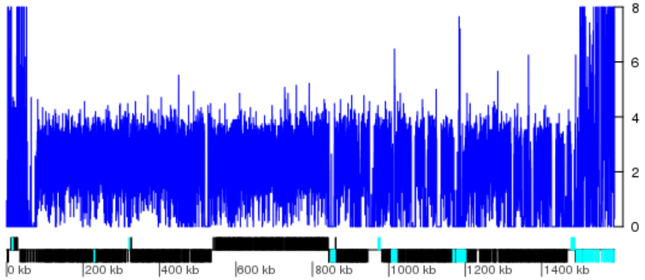

D7-Tb927.05_v5.1

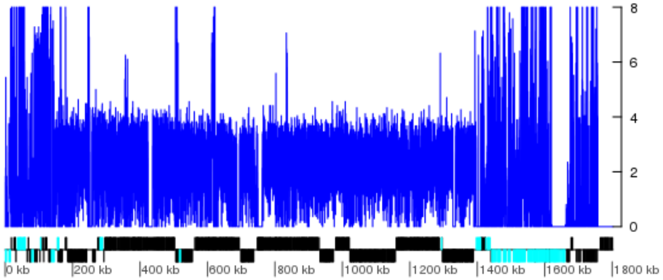

D7-Tb927.06_v5.1

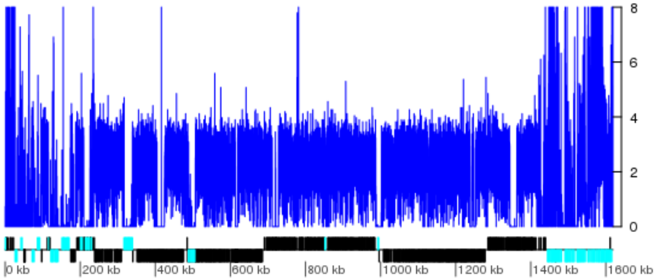

D7-Tb927.07_v5.1

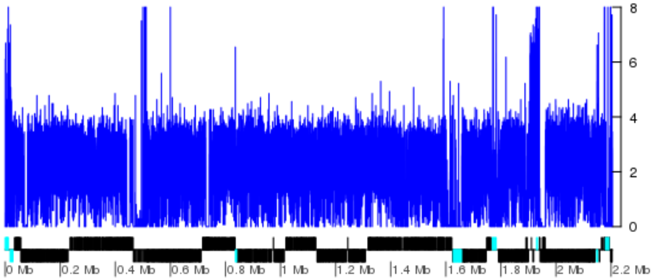

D7-Tb927.08_v5.1

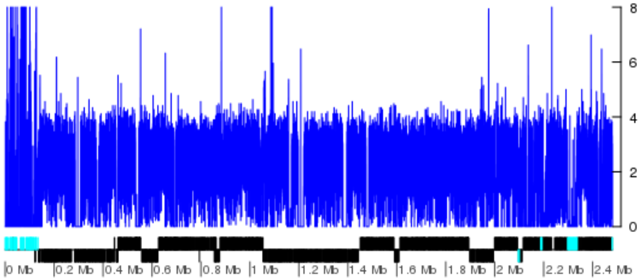

D7-Tb927.09_v5.1

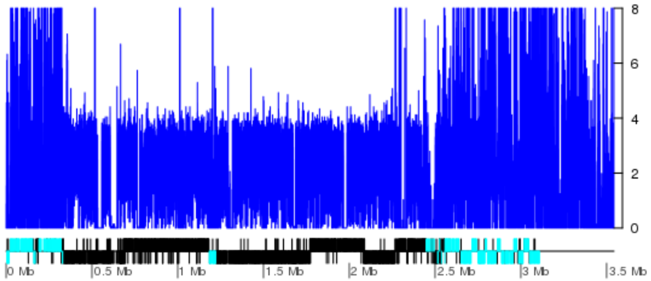

D7-Tb927.10_v5.1

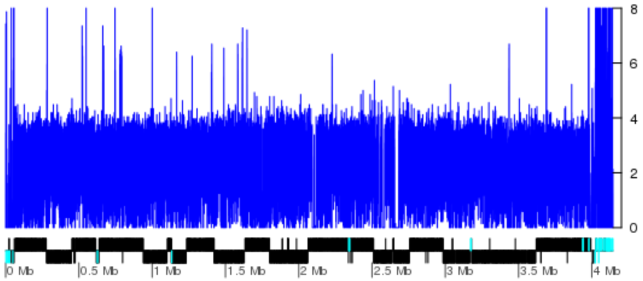

D7-Tb927.11_v5.1

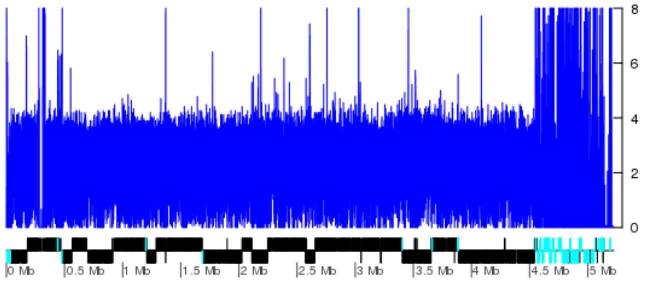

Isolate

D11

D11-Tb927.01_v5.1

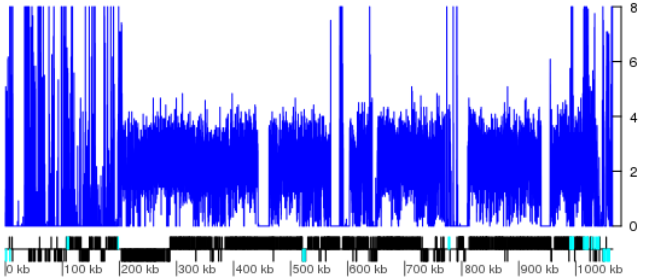

D11-Tb927.02_v5.1

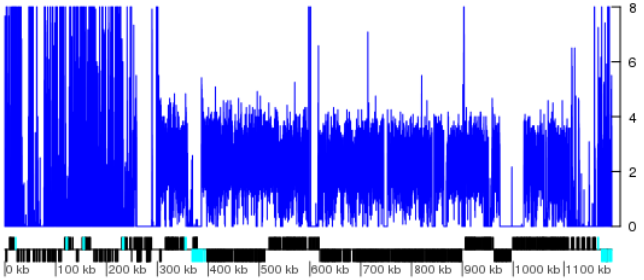

D11-Tb927.03_v5.1

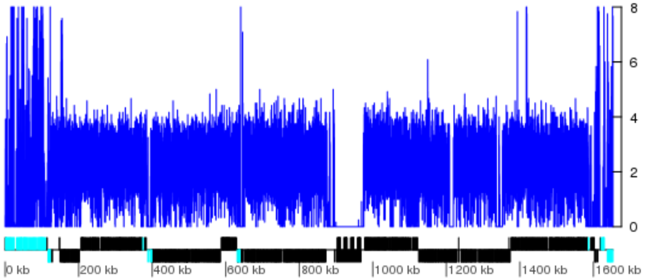

D11-Tb927.04_v5.1

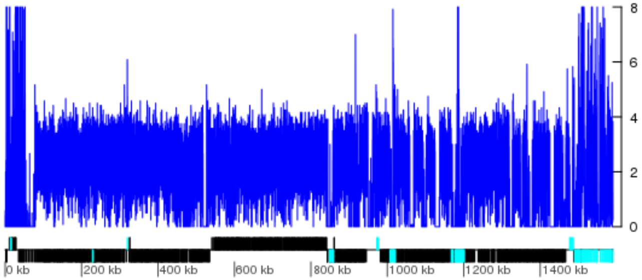

D11-Tb927.05_v5.1

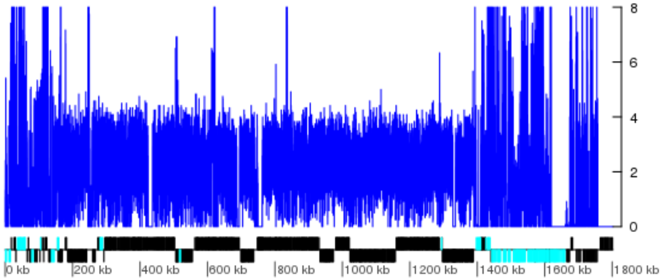

D11-Tb927.06_v5.1

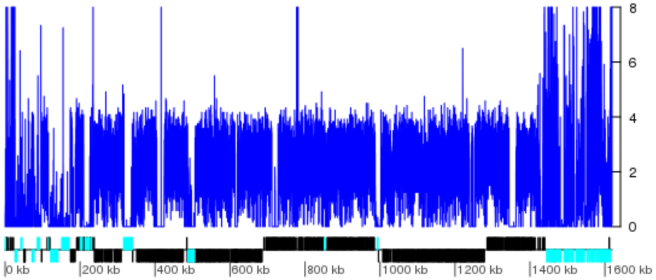

D11-Tb927.07_v5.1

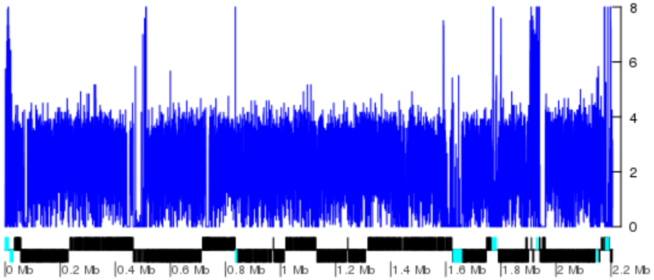

D11-Tb927.08_v5.1

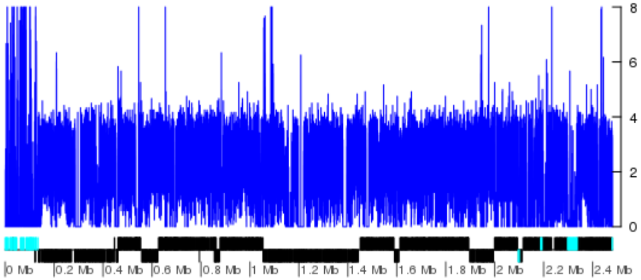

D11-Tb927.09_v5.1

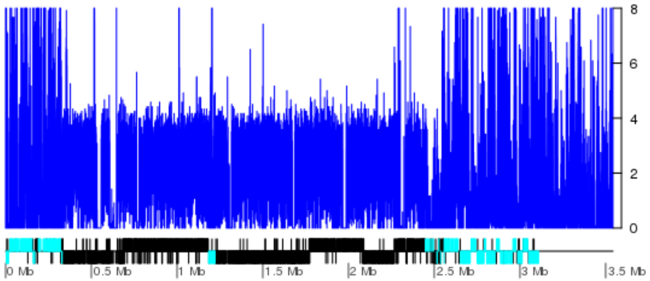

D11-Tb927.10_v5.1

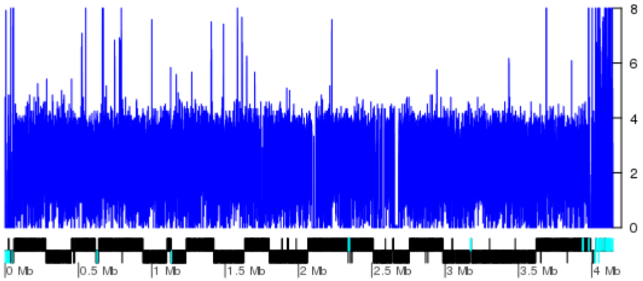

D11-Tb927.11_v5.1

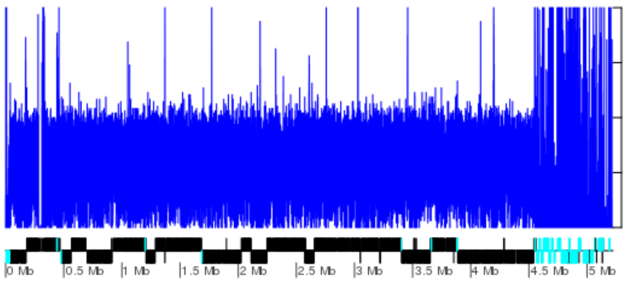

Isolate
D16

D16-Tb927.01_v5.1

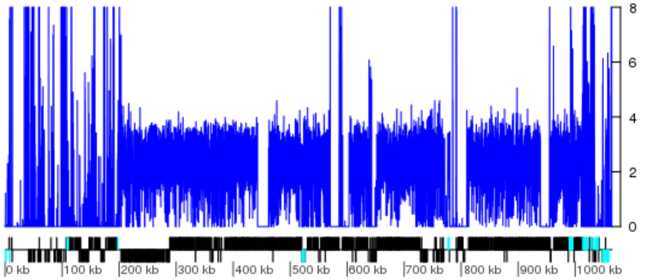

D16-Tb927.02_v5.1

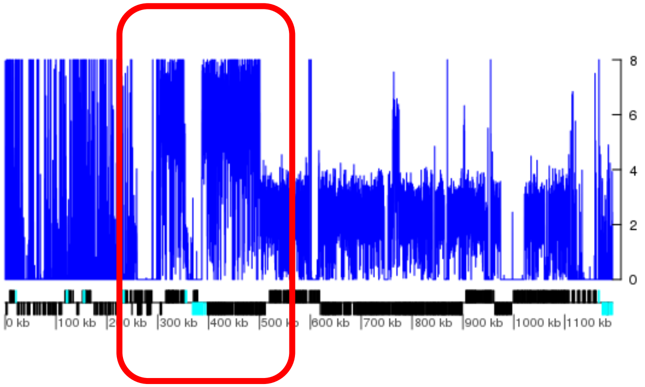

D16-Tb927.03_v5.1

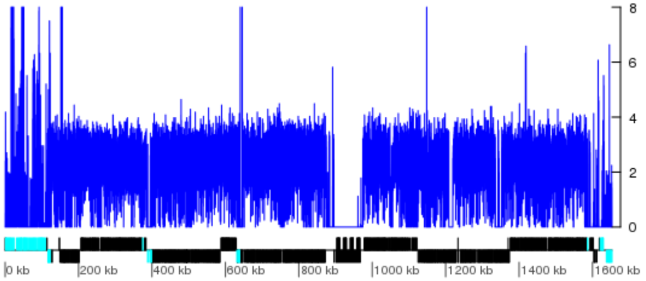

D16-Tb927.04_v5.1

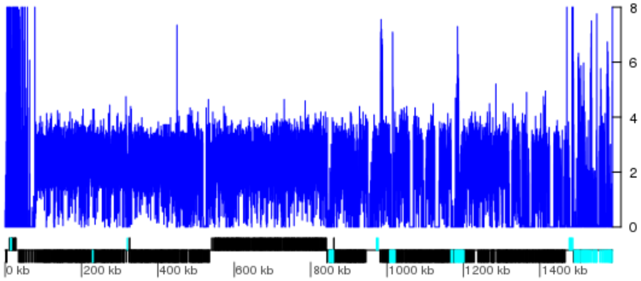

D16-Tb927.05_v5.1

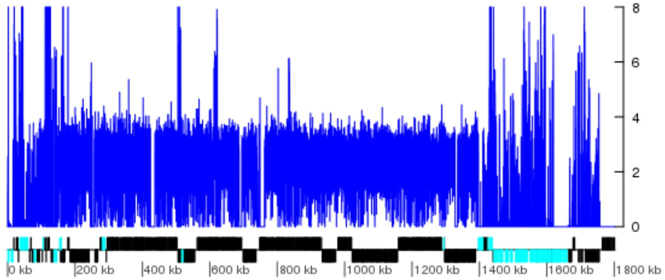

D16-Tb927.06_v5.1

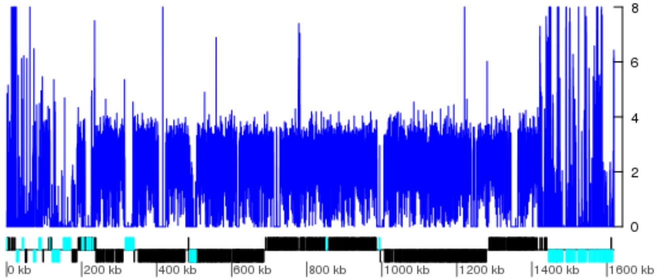

D16-Tb927.07_v5.1

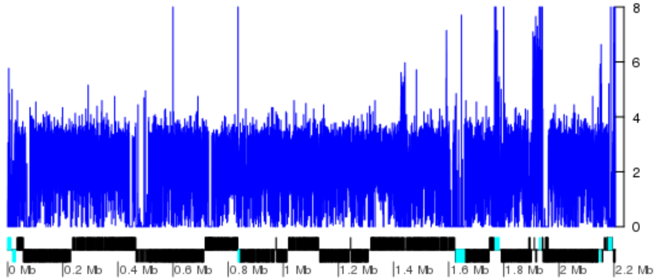

D16-Tb927.08_v5.1

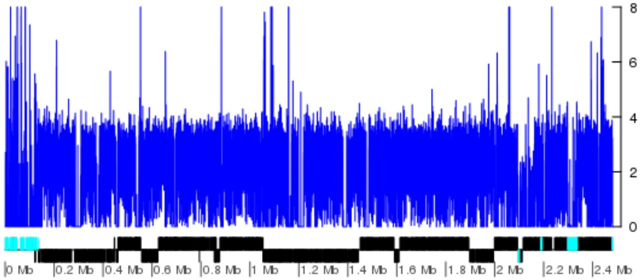

D16-Tb927.09_v5.1

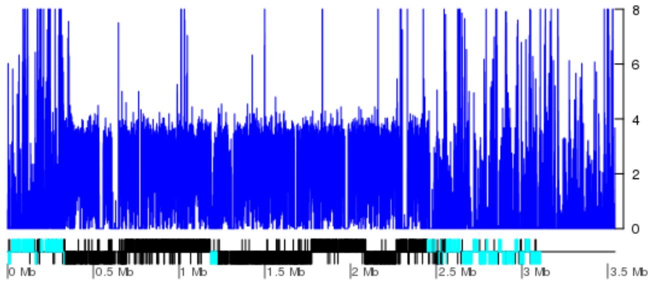

D16-Tb927.10_v5.1

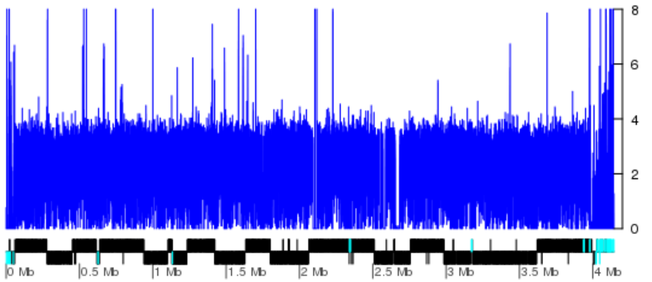

D16-Tb927.11_v5.1

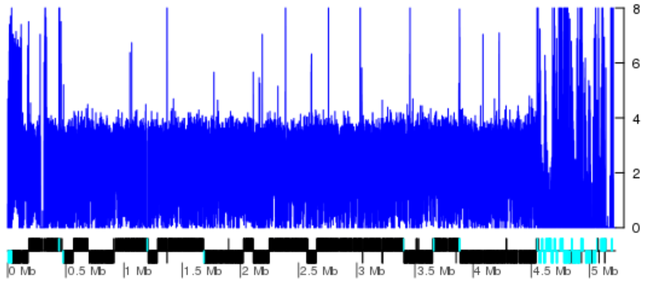

Isolate
Dog157

Dog157-Tb927.01_v5.1

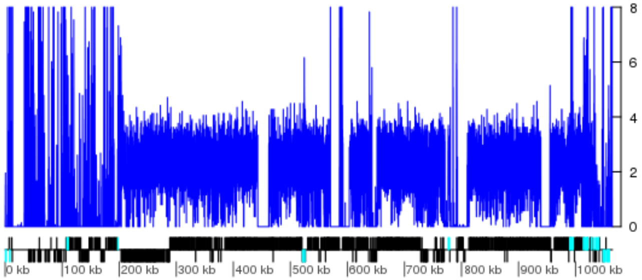

Dog157-Tb927.02_v5.1

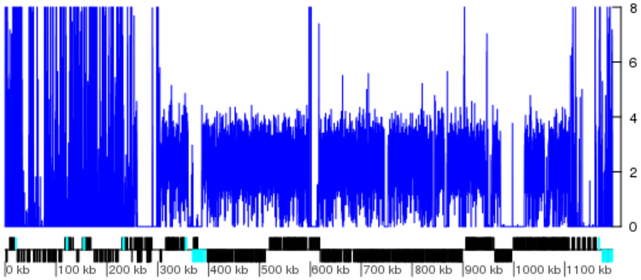

Dog157-Tb927.03_v5.1

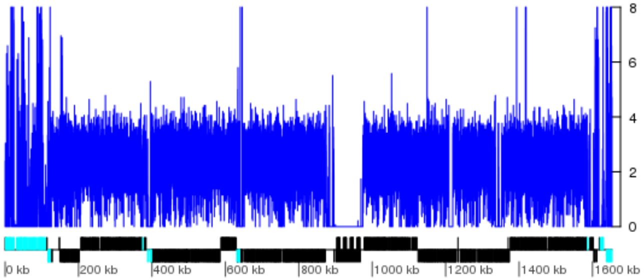

Dog157-Tb927.04_v5.1

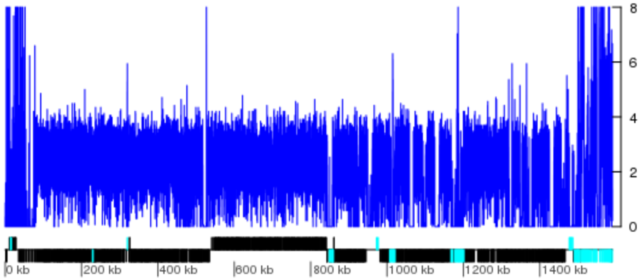

Dog157-Tb927.05_v5.1

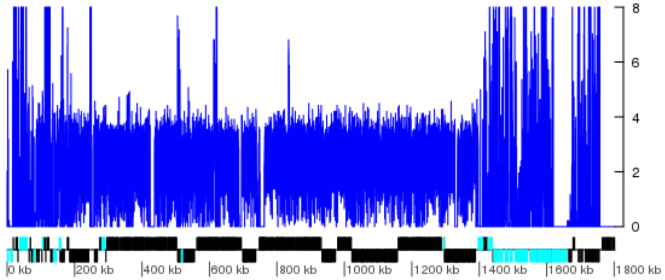

Dog157-Tb927.06_v5.1

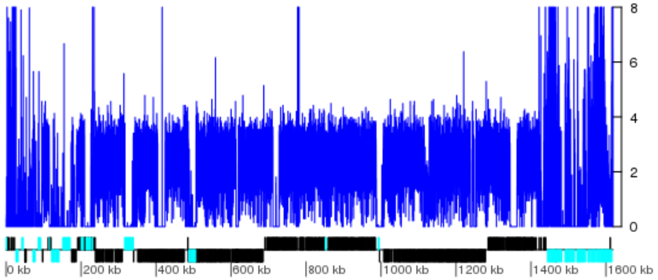

Dog157-Tb927.06_v5.1

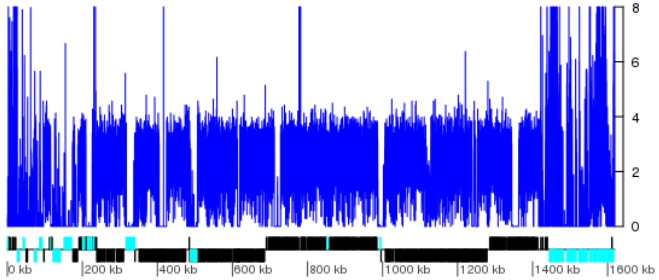

Dog157-Tb927.08_v5.1

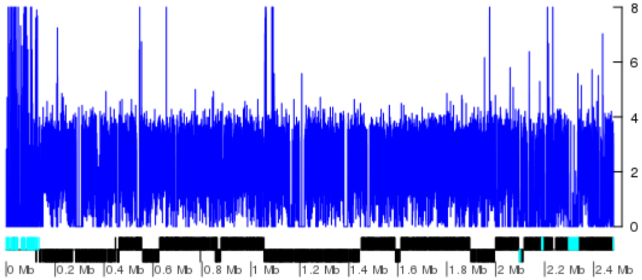

Dog157-Tb927.09_v5.1

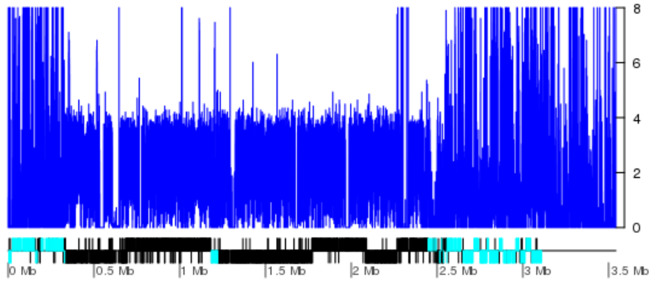

Dog157-Tb927.10_v5.1

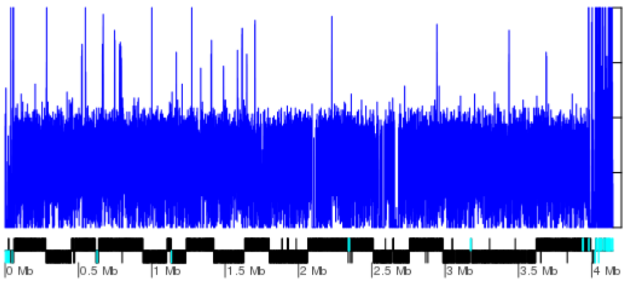

Dog157-Tb927.11_v5.1

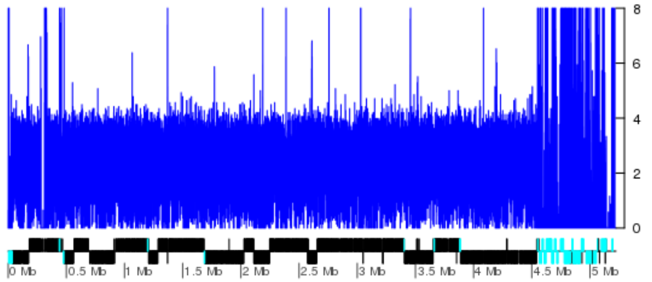

Isolate
I5

l5-Tb927.01_v5.1

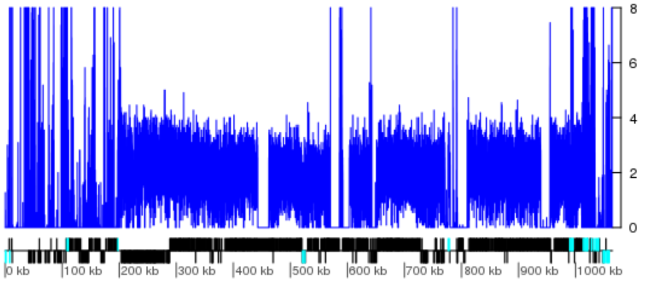

l5-Tb927.02_v5.1

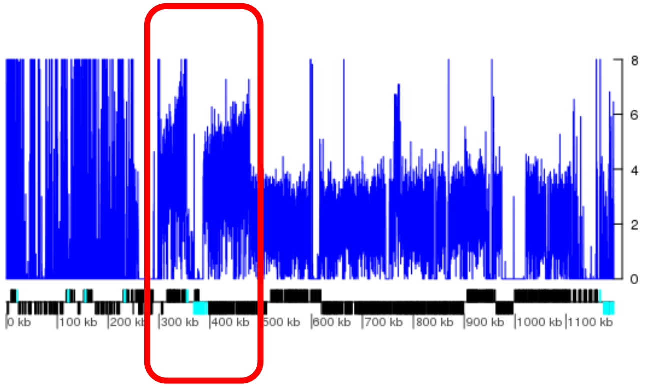

l5-Tb927.03_v5.1

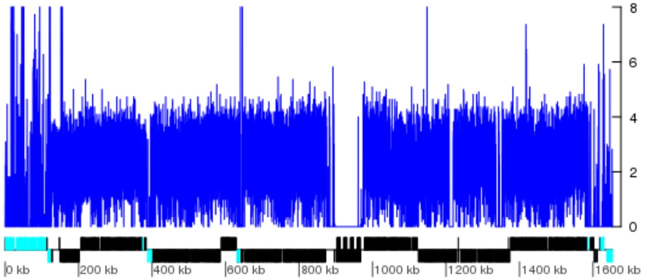

l5-Tb927.04_v5.1

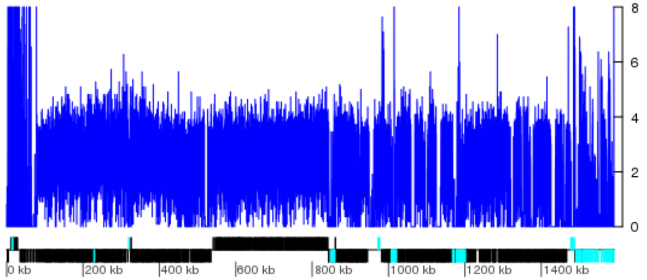

I5-Tb927.05_v5.1

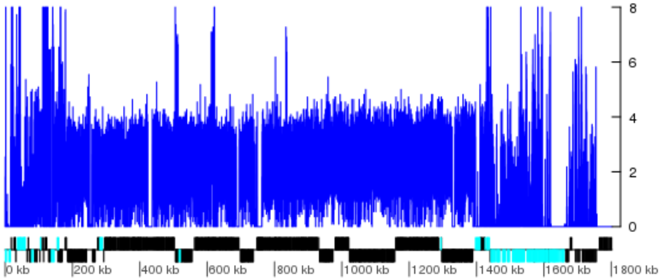

I5-Tb927.06_v5.1

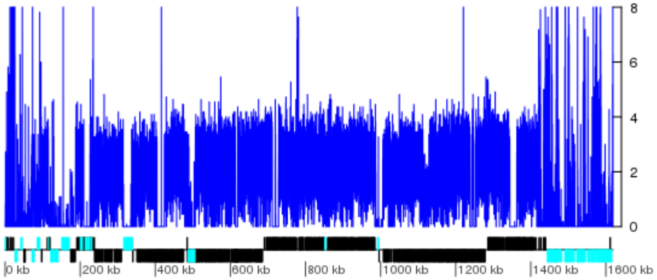

I5-Tb927.07_v5.1

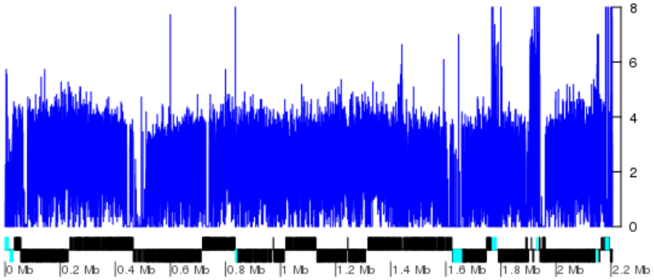

I5-Tb927.08_v5.1

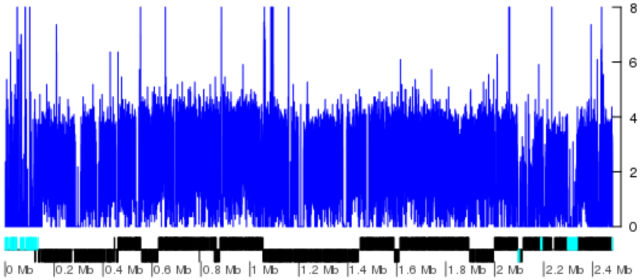

l5-Tb927.09_v5.1

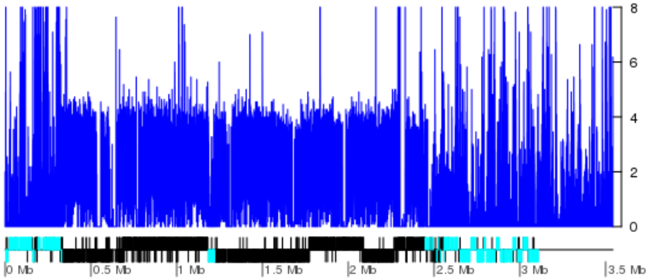

l5-Tb927.10_v5.1

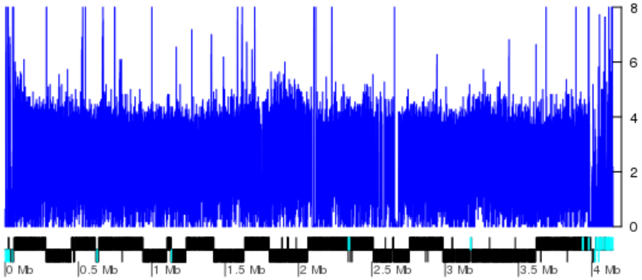

l5-Tb927.11_v5.1

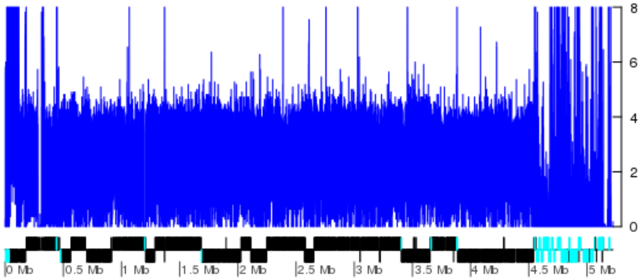

Isolate
I7

I7-Tb927.01_v5.1

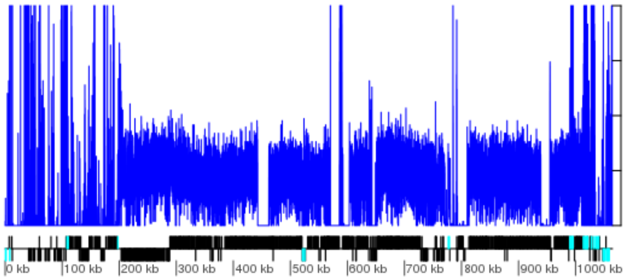

I7-Tb927.02_v5.1

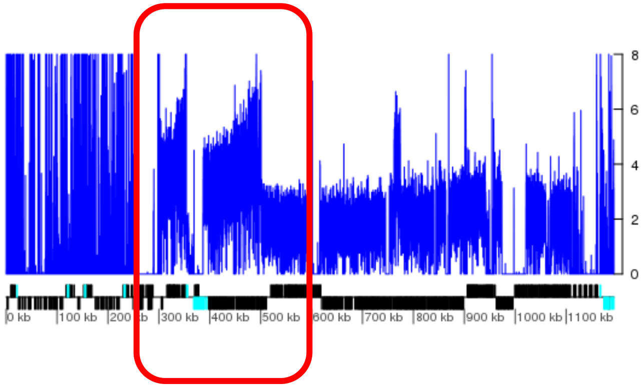

I7-Tb927.03_v5.1

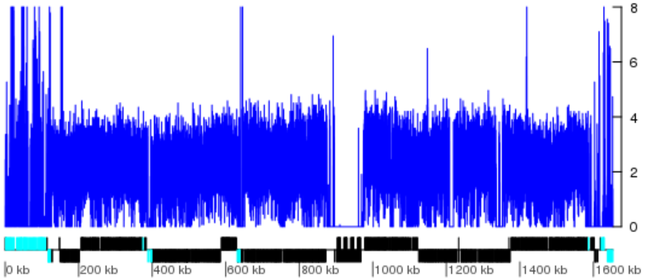

I7-Tb927.04_v5.1

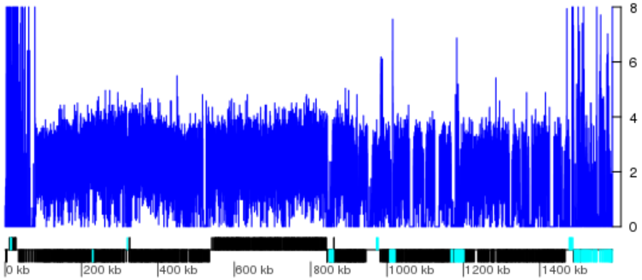

I7-Tb927.05_v5.1

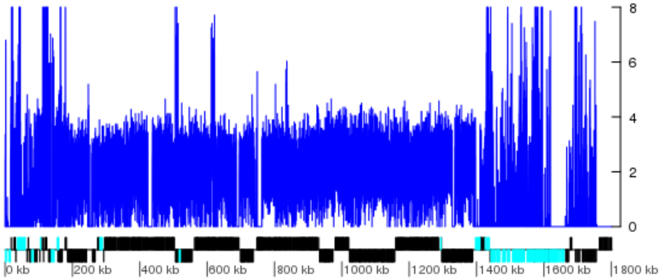

I7-Tb927.06_v5.1

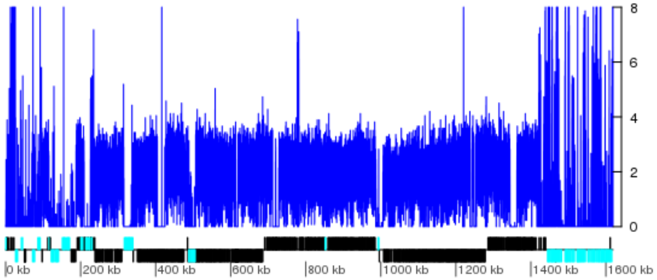

I7-Tb927.07_v5.1

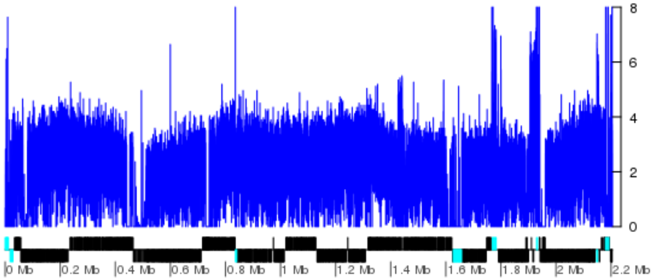

I7-Tb927.08_v5.1

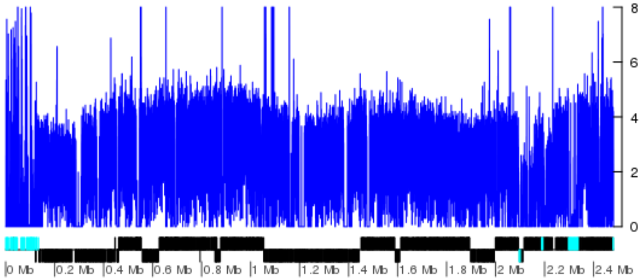

I7-Tb927.09_v5.1

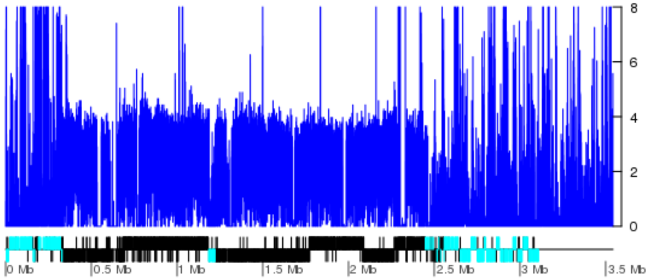

I7-Tb927.10_v5.1

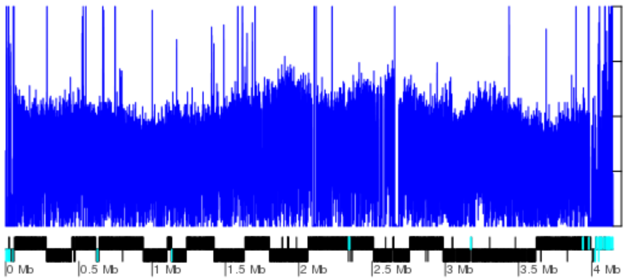

I7-Tb927.11_v5.1

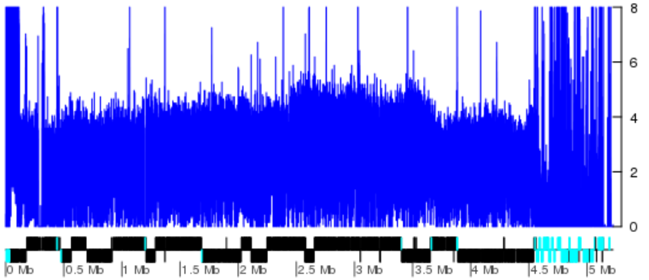

Isolate
Keko

KeKo-Tb927.01_v5.1

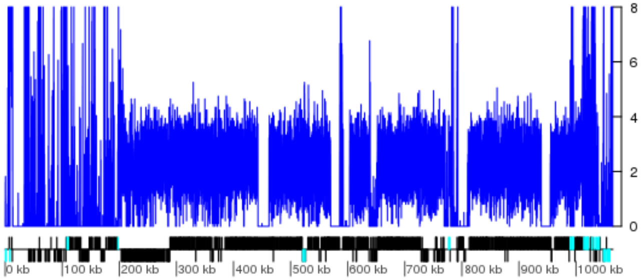

KeKo-Tb927.02_v5.1

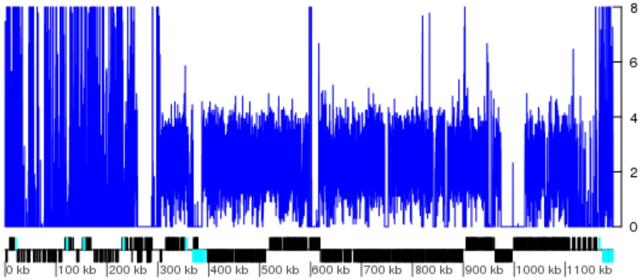

KeKo-Tb927.03_v5.1

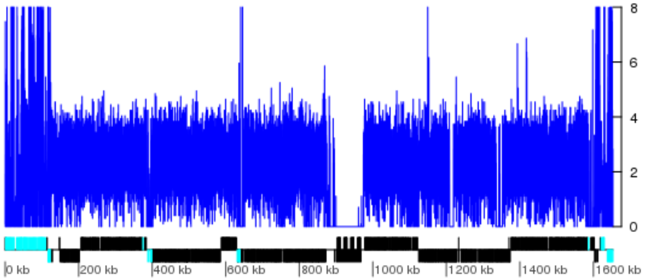

KeKo-Tb927.04_v5.1

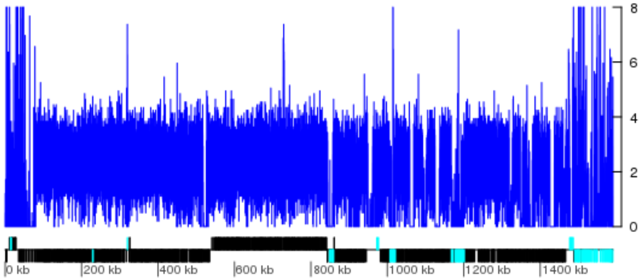

KeKo-Tb927.05_v5.1

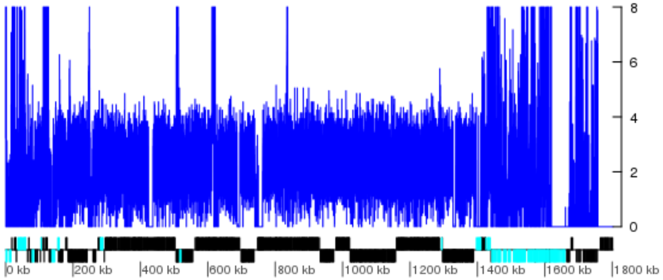

KeKo-Tb927.06_v5.1

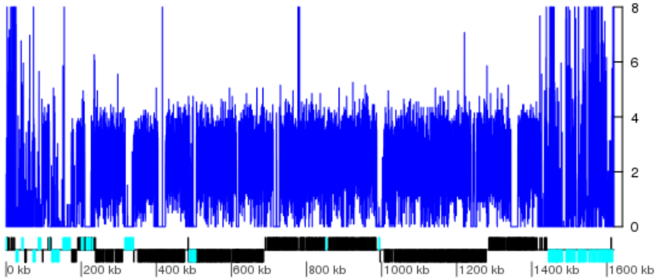

KeKo-Tb927.07_v5.1

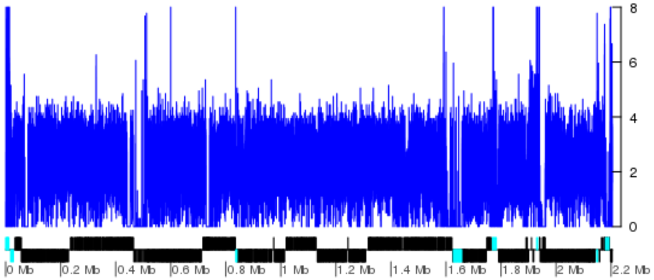

KeKo-Tb927.08_v5.1

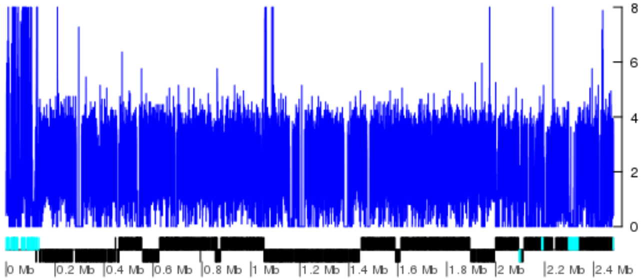

KeKo-Tb927.09_v5.1

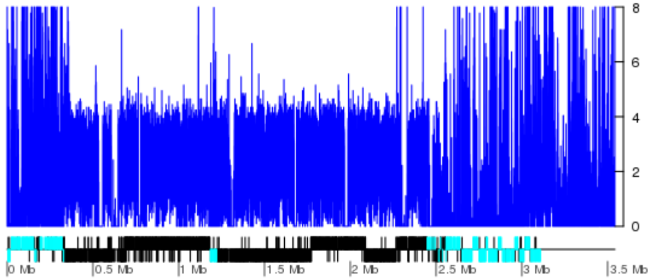

KeKo-Tb927.10_v5.1

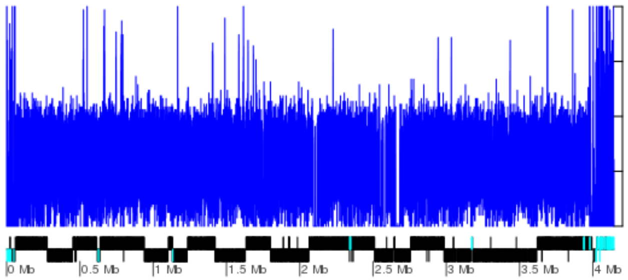

KeKo-Tb927.11_v5.1

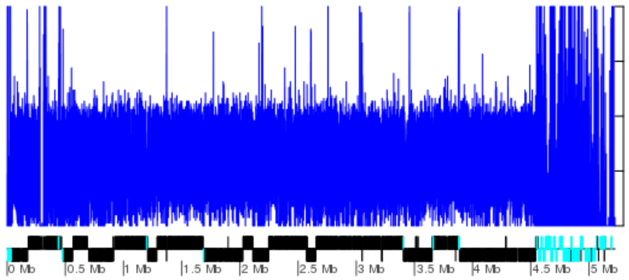

Isolate LWO07A

LWO07A-Tb927.01_v5.1

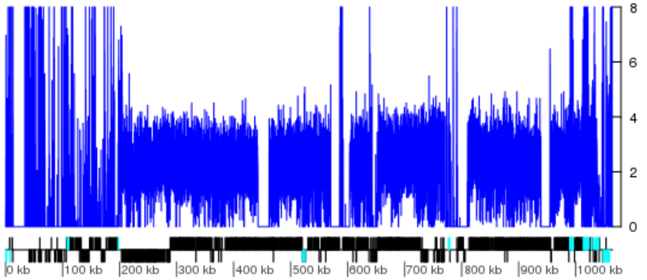

LWO07A-Tb927.02_v5.1

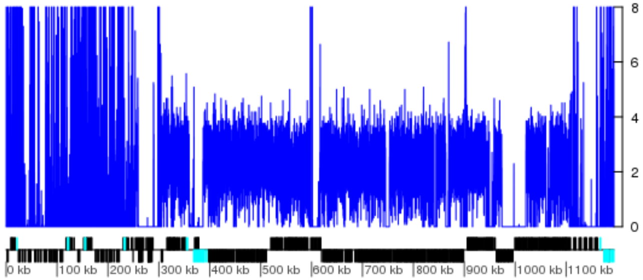

LWO07A-Tb927.03_v5.1

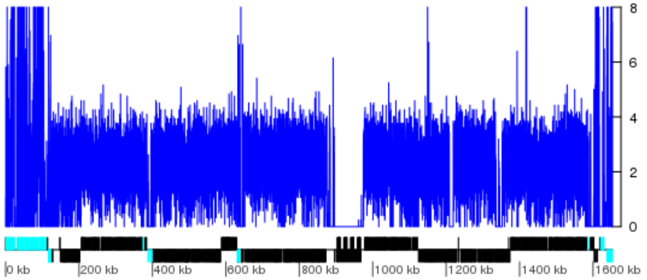

LWO07A-Tb927.04_v5.1

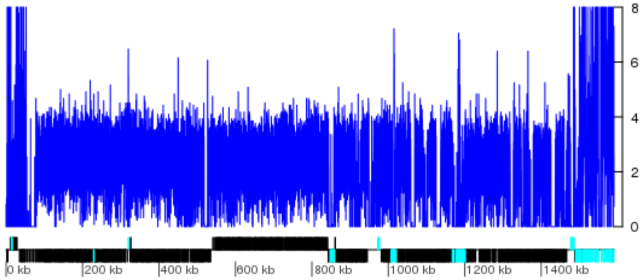

LWO07A-Tb927.05_v5.1

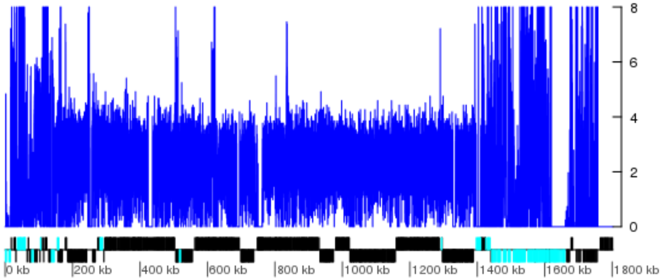

LWO07A-Tb927.06_v5.1

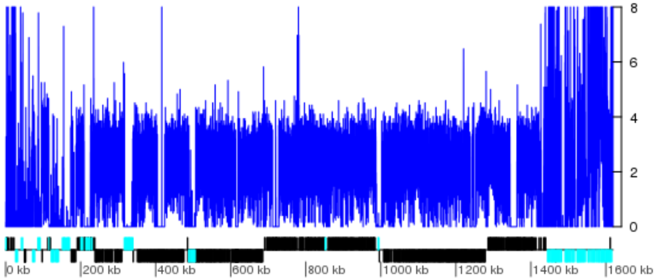

LWO07A-Tb927.07_v5.1

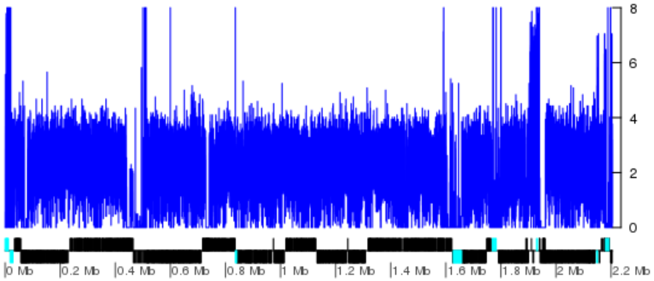

LWO07A-Tb927.08_v5.1

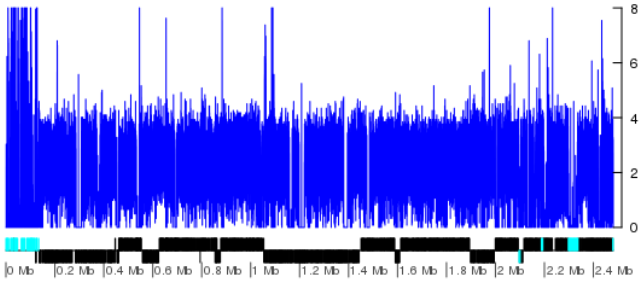

LWO07A-Tb927.09_v5.1

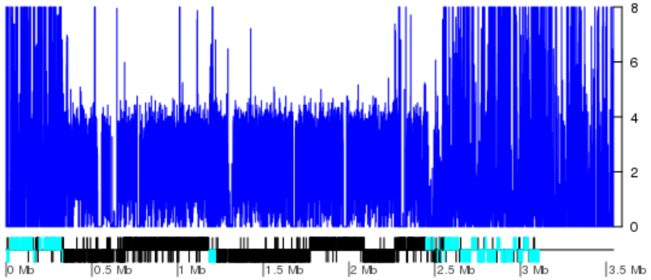

LWO07A-Tb927.10_v5.1

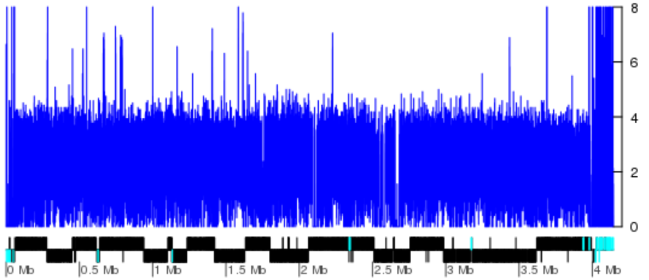

LWO07A-Tb927.11_v5.1

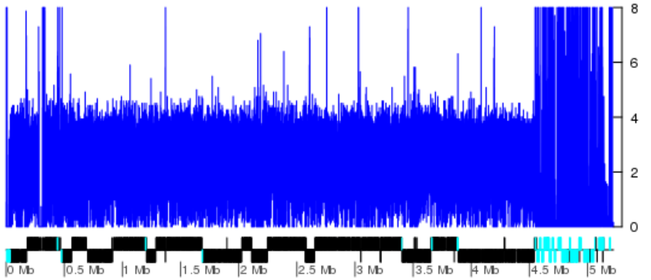

Isolate
LWO011A

LWO11A-Tb927.01_v5.1

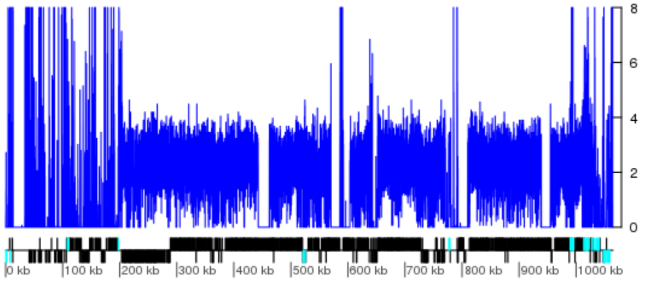

LWO11A-Tb927.02_v5.1

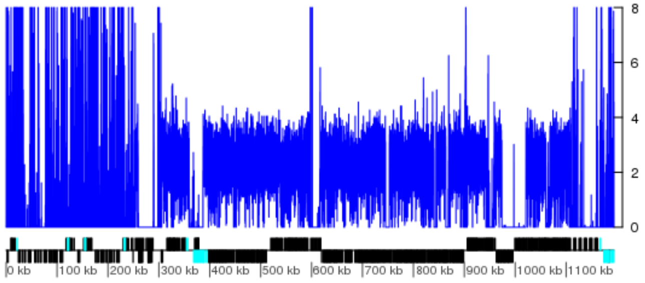

LWO11A-Tb927.03_v5.1

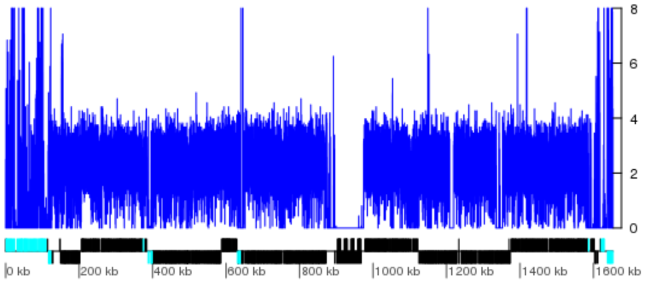

LWO11A-Tb927.04_v5.1

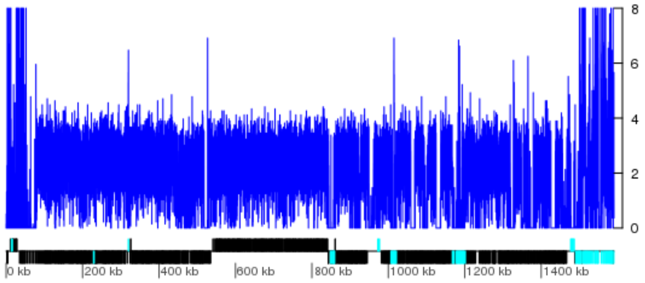

LWO11A-Tb927.05_v5.1

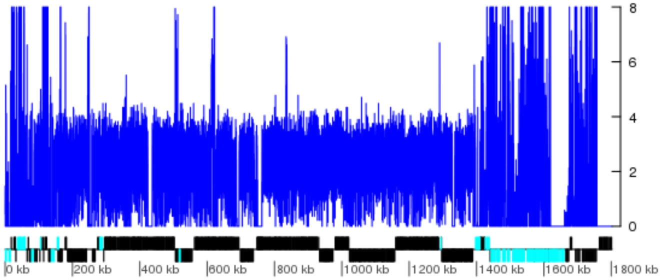

LWO11A-Tb927.06_v5.1

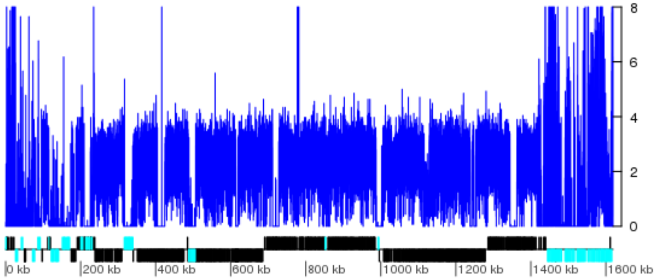

LWO11A-Tb927.07_v5.1

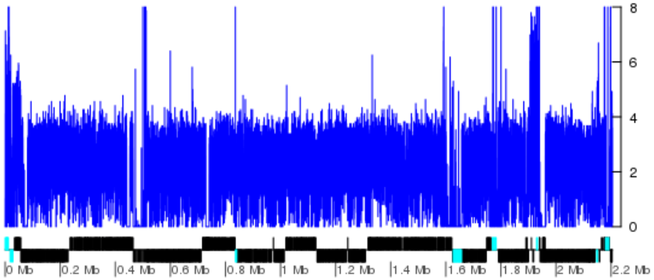

LWO11A-Tb927.08_v5.1

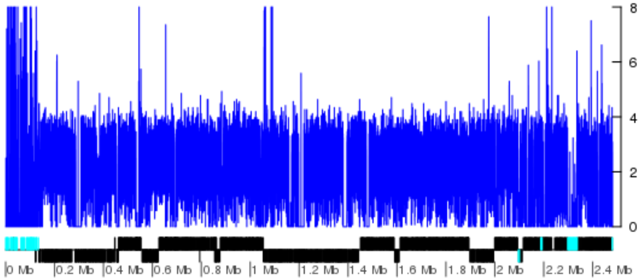

LWO11A-Tb927.09_v5.1

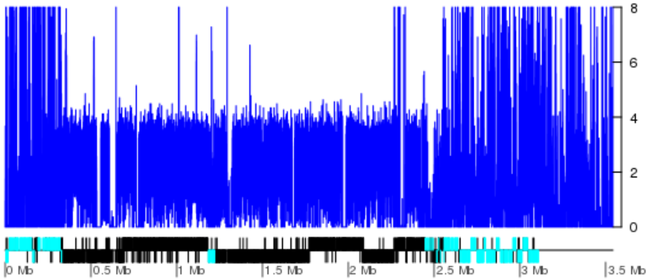

LWO11A-Tb927.10_v5.1

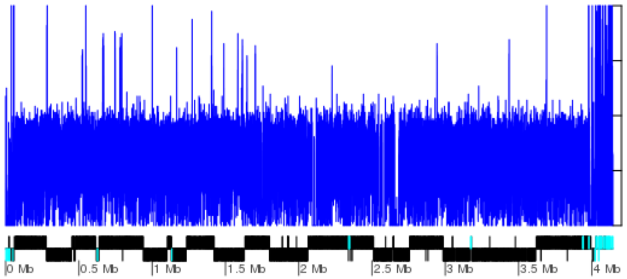

LWO11A-Tb927.11_v5.1

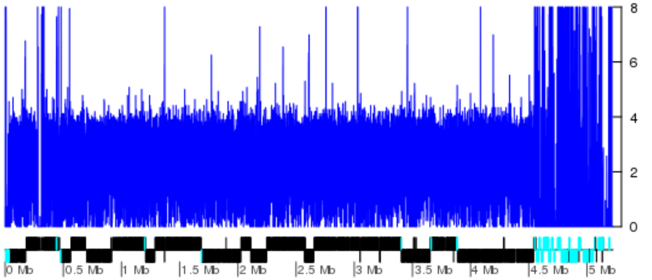

Isolate LWO024A

LWO11A-Tb927.01_v5.1

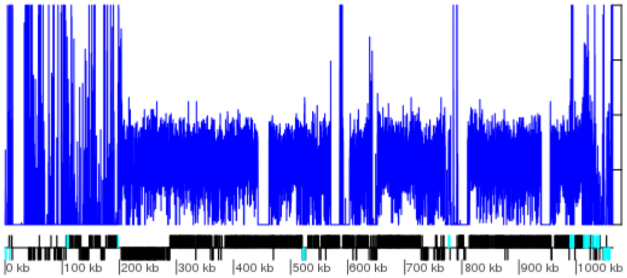

LWO11A-Tb927.02_v5.1

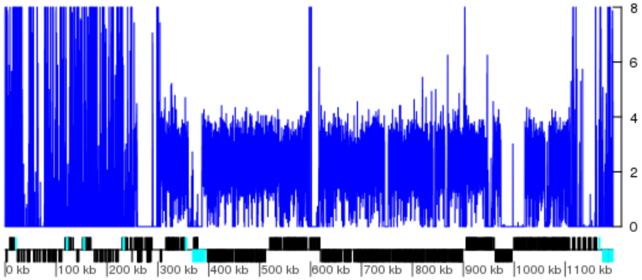

LWO11A-Tb927.03_v5.1

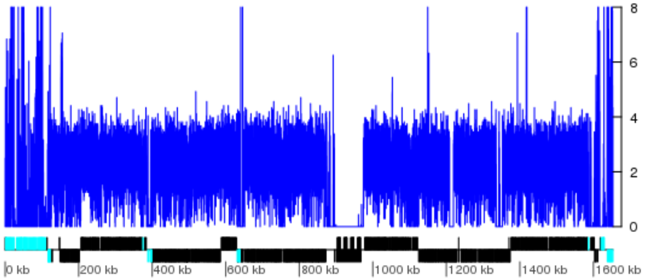

LWO11A-Tb927.04_v5.1

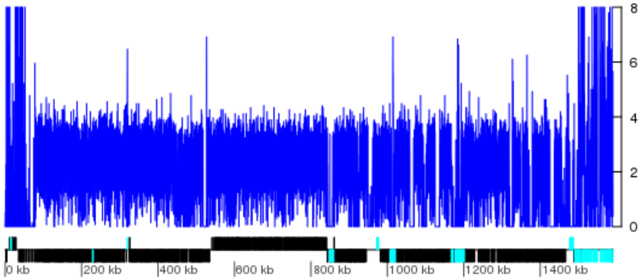

LWO11A-Tb927.05_v5.1

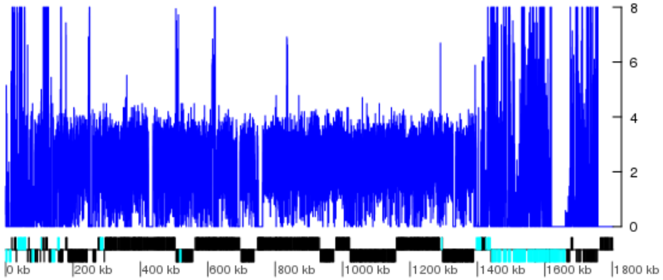

LWO11A-Tb927.06_v5.1

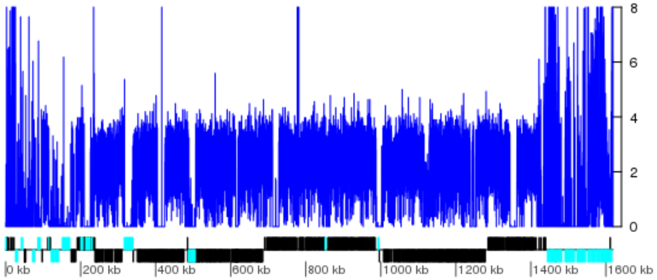

LWO11A-Tb927.07_v5.1

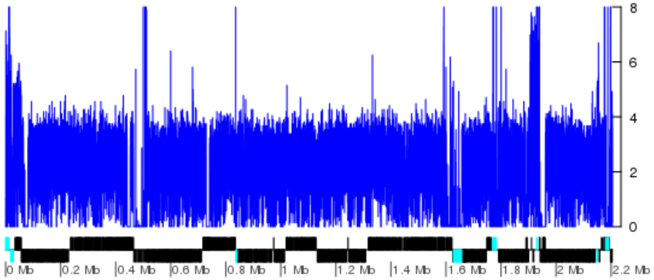

LWO11A-Tb927.08_v5.1

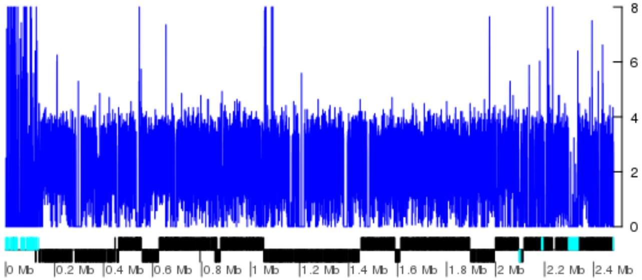

LWO11A-Tb927.09_v5.1

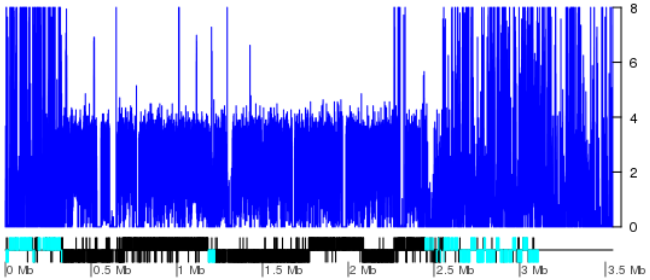

LWO11A-Tb927.10_v5.1

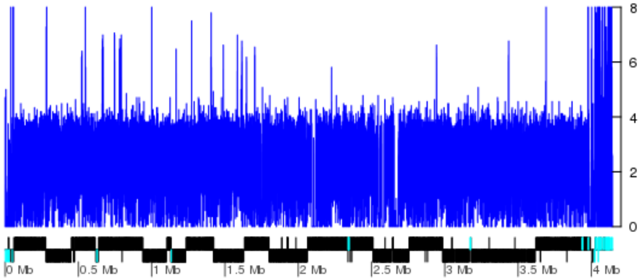

LWO11A-Tb927.11_v5.1

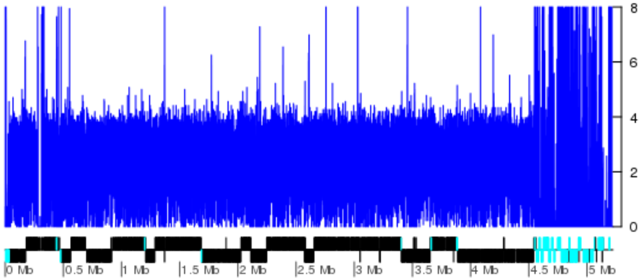

Isolate LWO030A

LWO30A-Tb927.01_v5.1

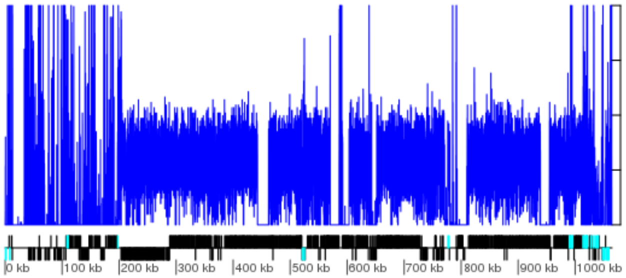

LWO30A-Tb927.02_v5.1

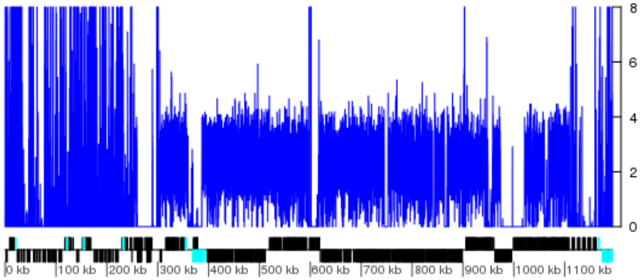

LWO30A-Tb927.03_v5.1

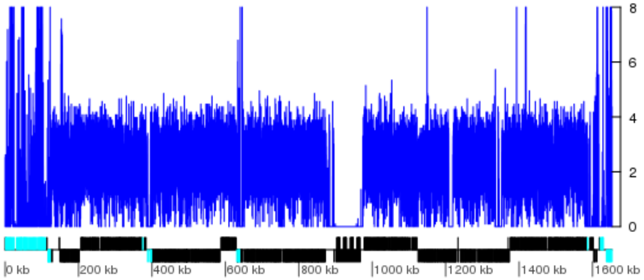

LWO30A-Tb927.04_v5.1

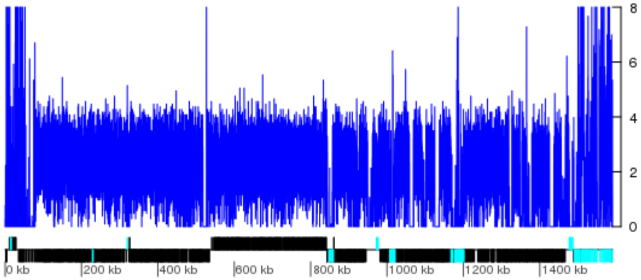

LWO30A-Tb927.05_v5.1

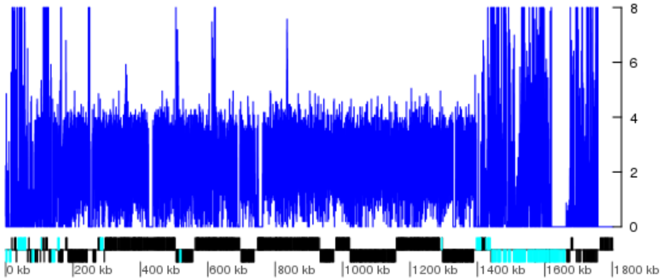

LWO30A-Tb927.06_v5.1

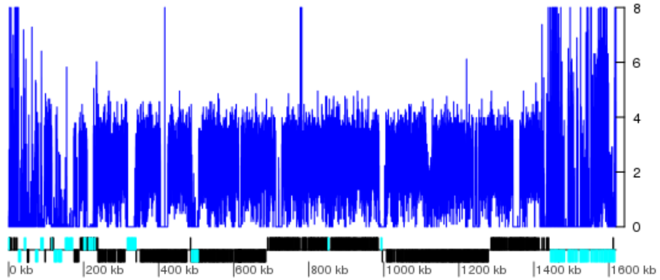

LWO30A-Tb927.07_v5.1

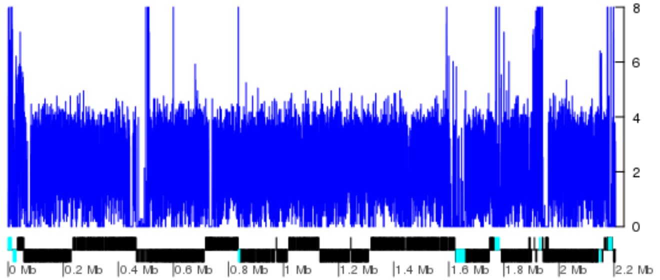

LWO30A-Tb927.08_v5.1

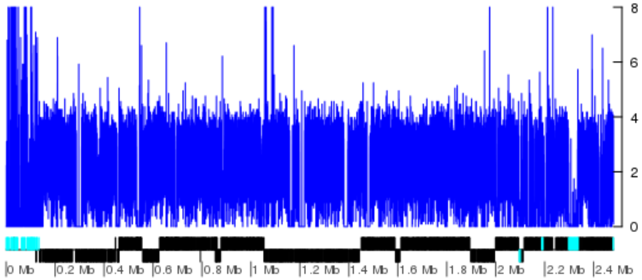

LWO30A-Tb927.09_v5.1

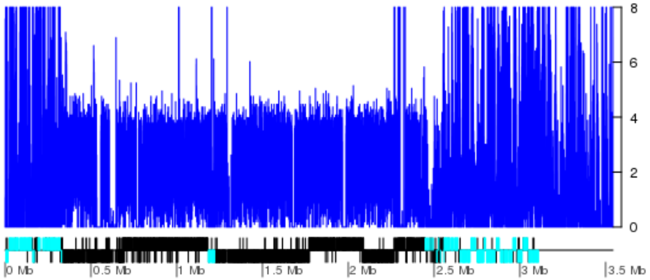

LWO30A-Tb927.10_v5.1

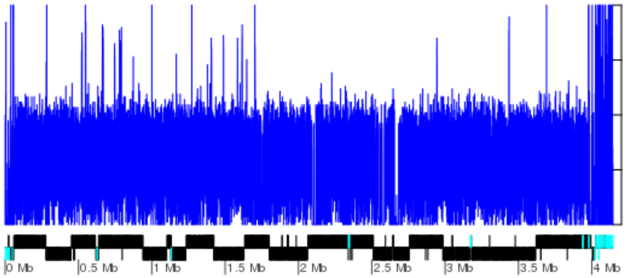

LWO30A-Tb927.11_v5.1

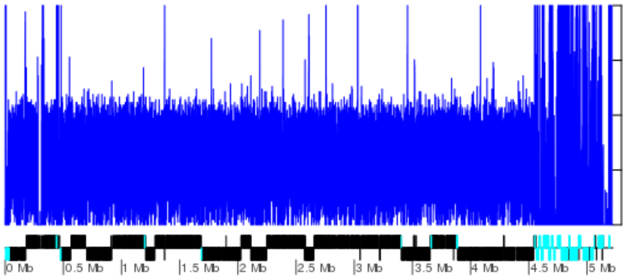

Isolate
LWO0150A

LWO150A-Tb927.01_v5.1

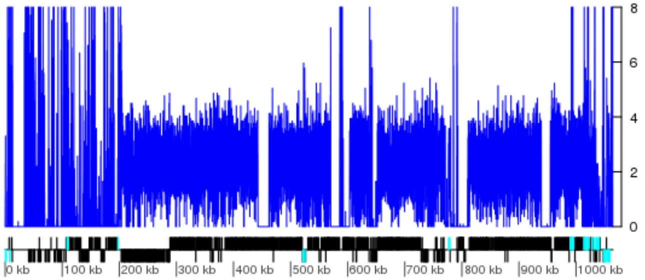

LWO150A-Tb927.02_v5.1

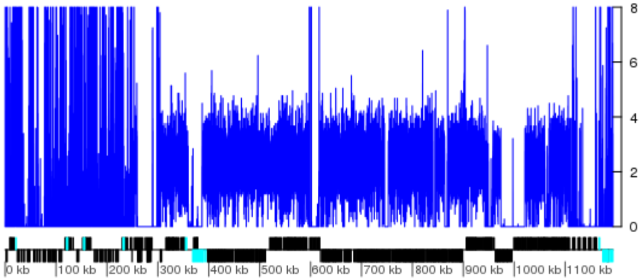

LWO150A-Tb927.03_v5.1

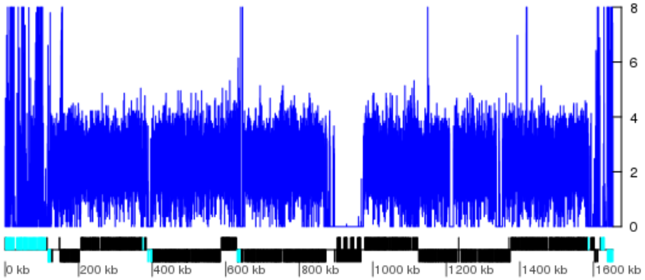

LWO150A-Tb927.04_v5.1

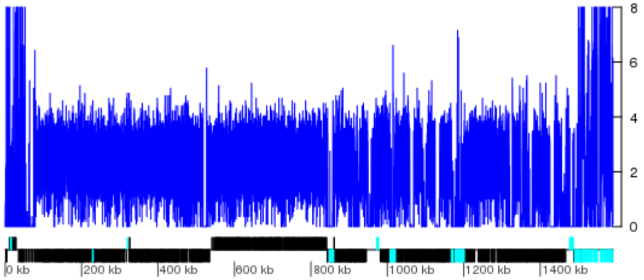

LWO150A-Tb927.05_v5.1

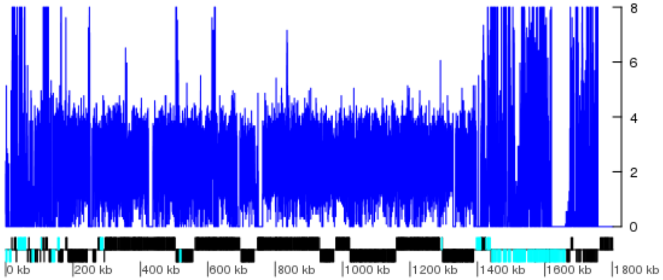

LWO150A-Tb927.06_v5.1

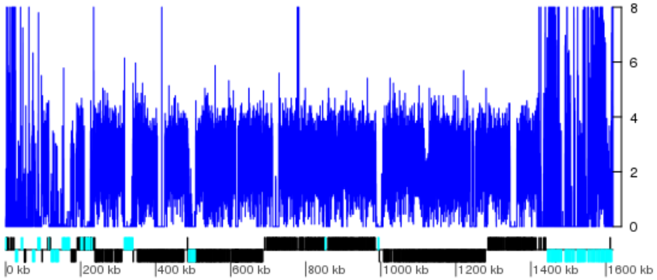

LWO150A-Tb927.07_v5.1

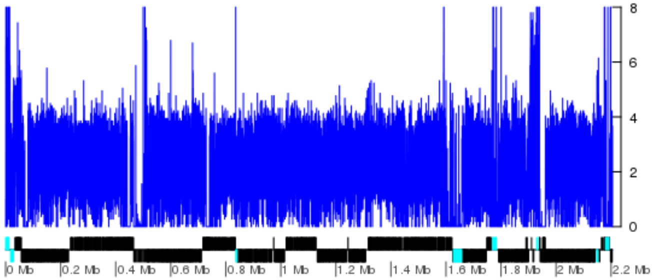

LWO150A-Tb927.08_v5.1

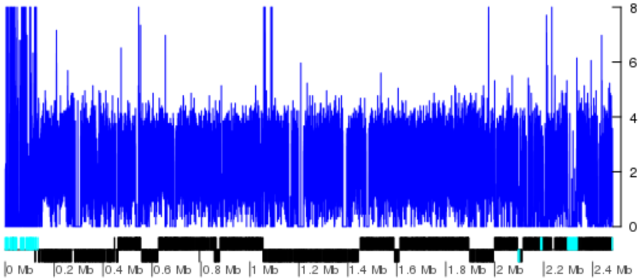

LWO150A-Tb927.09_v5.1

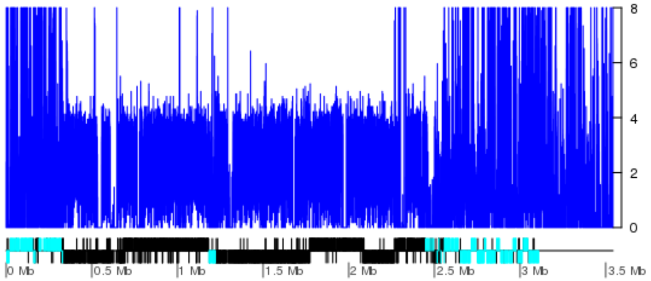

LWO150A-Tb927.10_v5.1

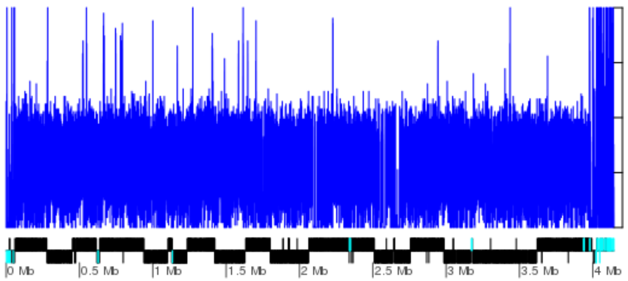

LWO150A-Tb927.11_v5.1

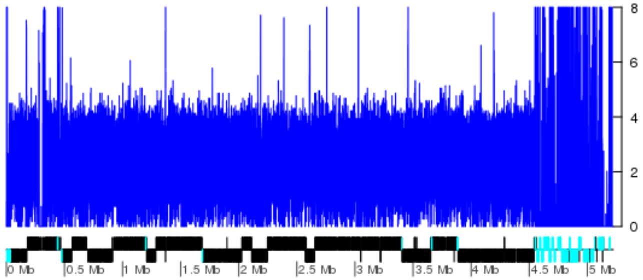

Isolate
Okware

Okware-Tb927.01_v5.1

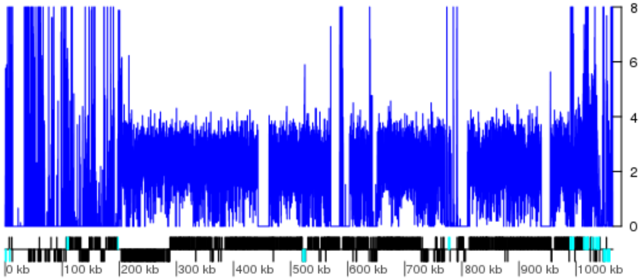

Okware-Tb927.02_v5.1

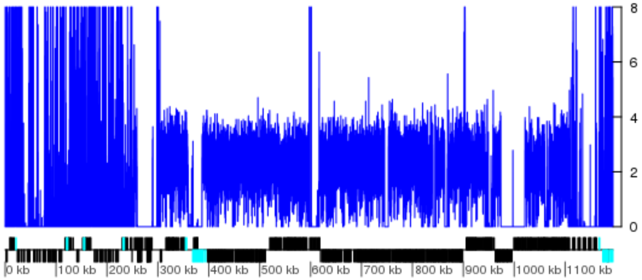

Okware-Tb927.03_v5.1

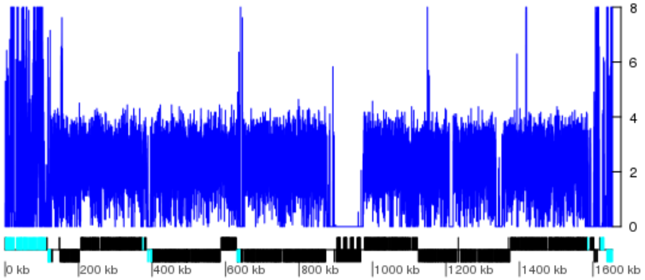

Okware-Tb927.04_v5.1

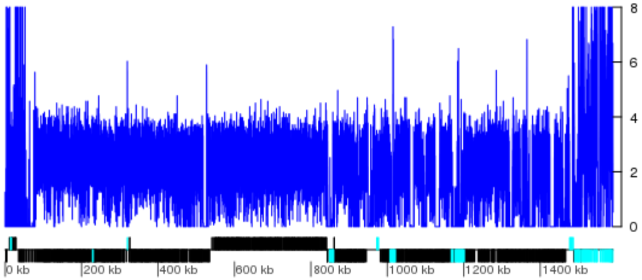

Okware-Tb927.05_v5.1

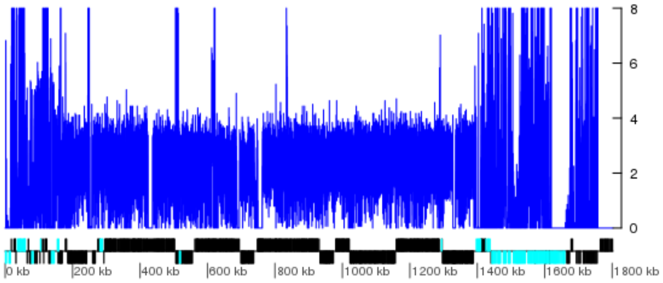

Okware-Tb927.06_v5.1

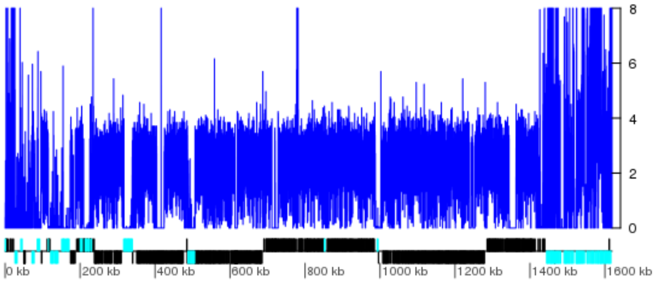

Okware-Tb927.07_v5.1

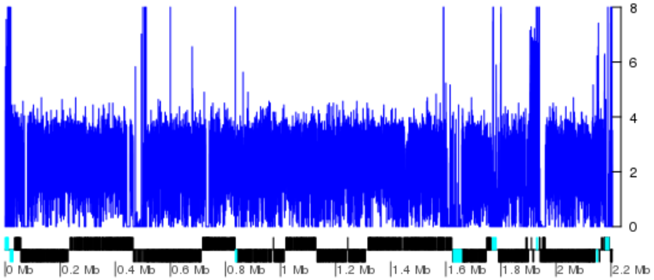

Okware-Tb927.08_v5.1

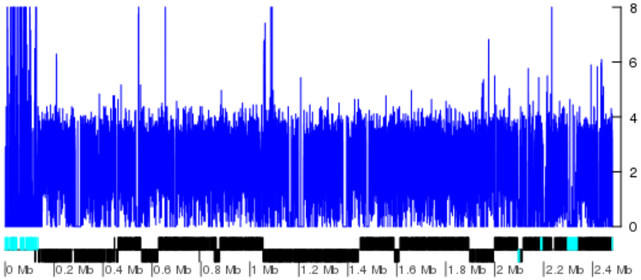

Okware-Tb927.09_v5.1

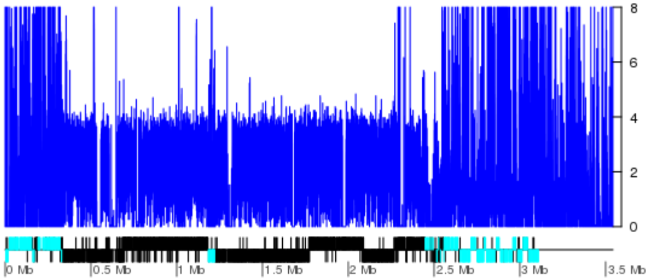

Okware-Tb927.10_v5.1

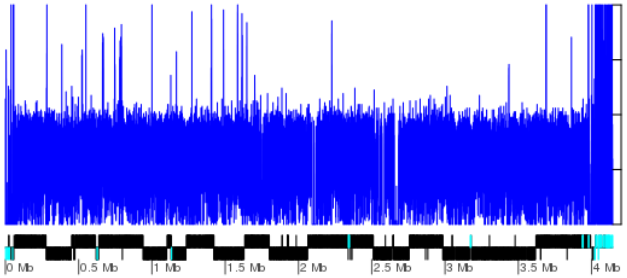

Okware-Tb927.11_v5.1

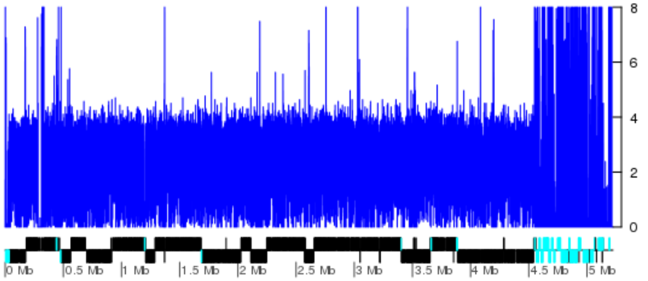

Isolate
STIB348TBABB

STIB348TBABB-Tb927.01_v5.1

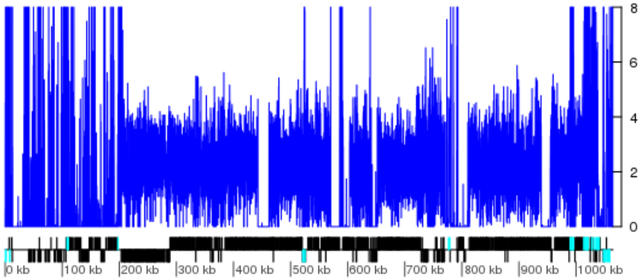

STIB348TBABB-Tb927.02_v5.1

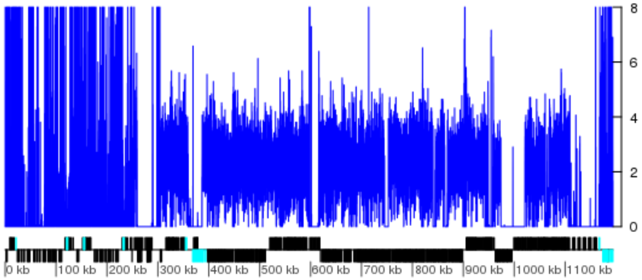

STIB348TBABB-Tb927.03_v5.1

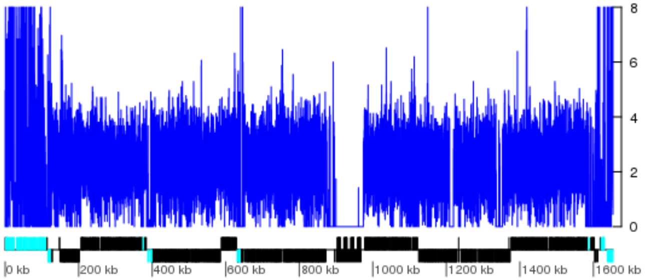

STIB348TBABB-Tb927.04_v5.1

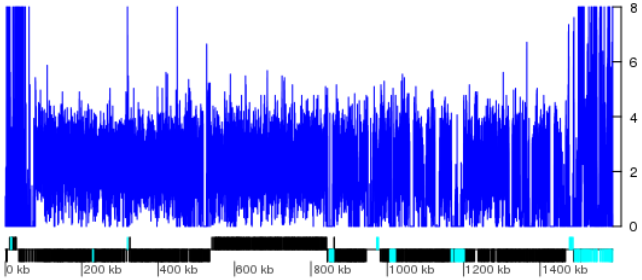

STIB348TBABB-Tb927.05_v5.1

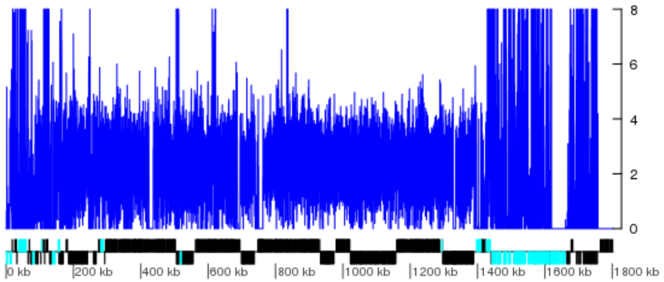

STIB348TBABB-Tb927.06_v5.1

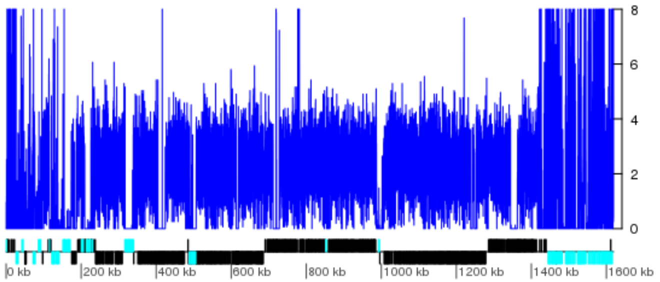

STIB348TBABB-Tb927.07_v5.1

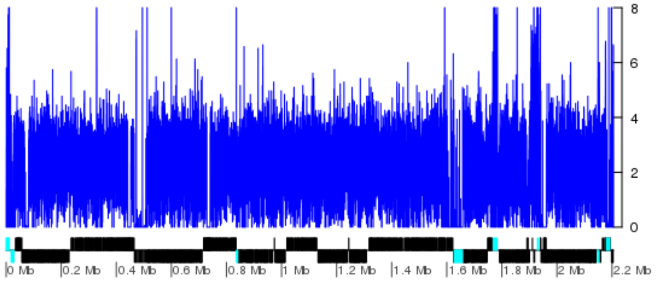

STIB348TBABB-Tb927.08_v5.1

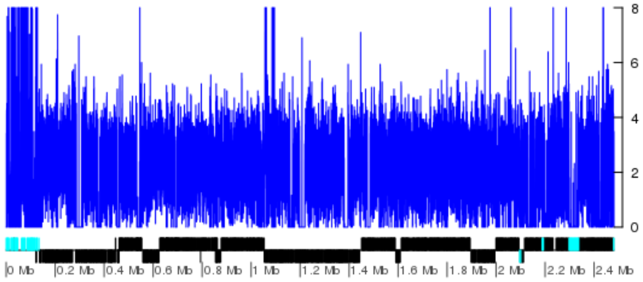

STIB348TBABB-Tb927.09_v5.1

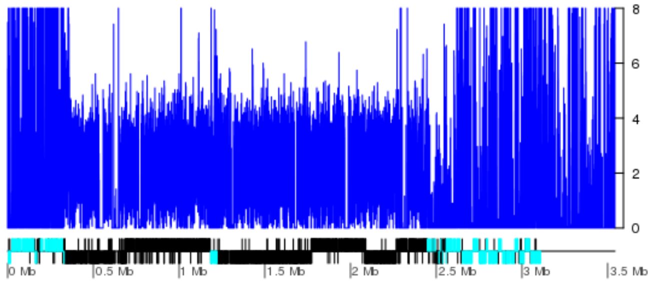

STIB348TBABB-Tb927.10_v5.1

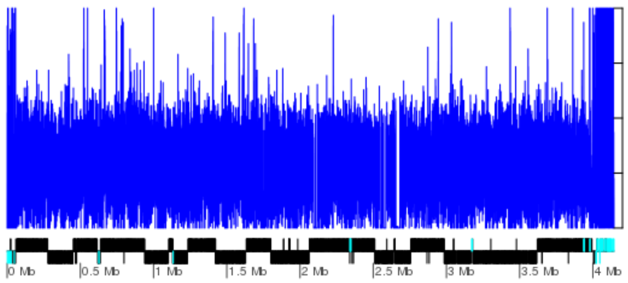

STIB348TBABB-Tb927.10_v5.1

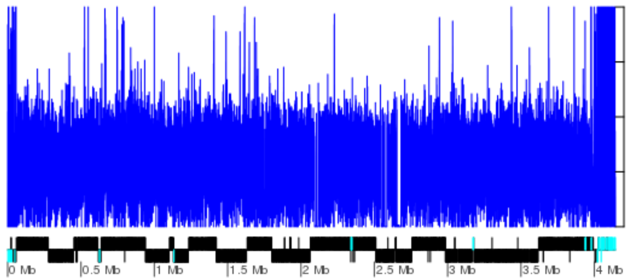

Isolate STIB704C

STIB704C-Tb927.01_v5.1

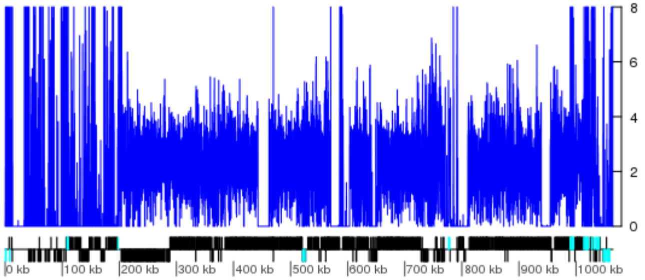

STIB704C-Tb927.02_v5.1

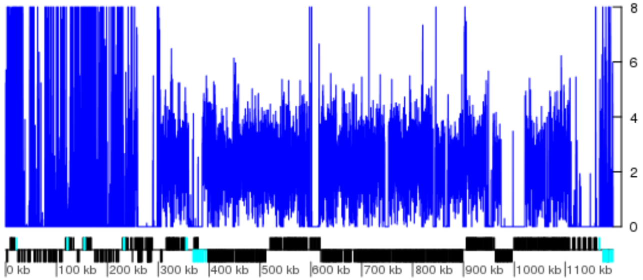

STIB704C-Tb927.03_v5.1

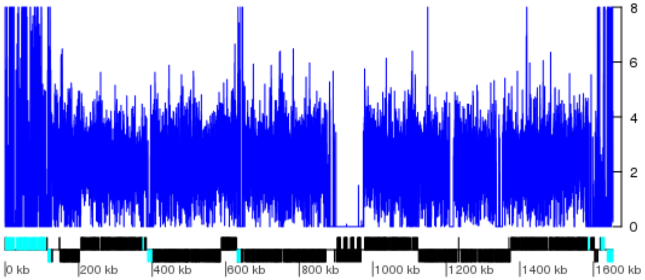

STIB704C-Tb927.04_v5.1

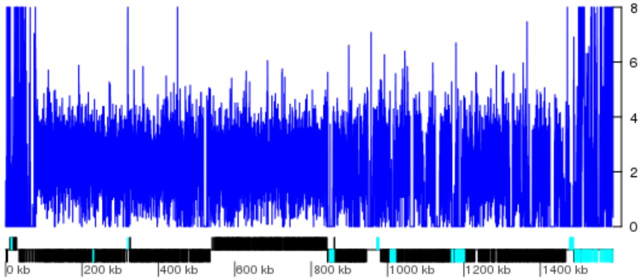

STIB704C-Tb927.05_v5.1

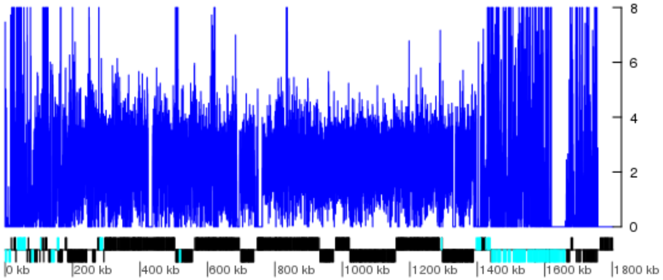

STIB704C-Tb927.06_v5.1

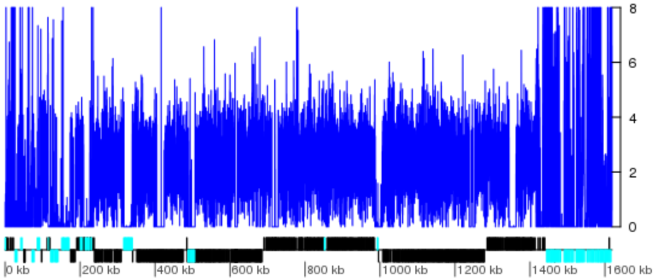

STIB704C-Tb927.07_v5.1

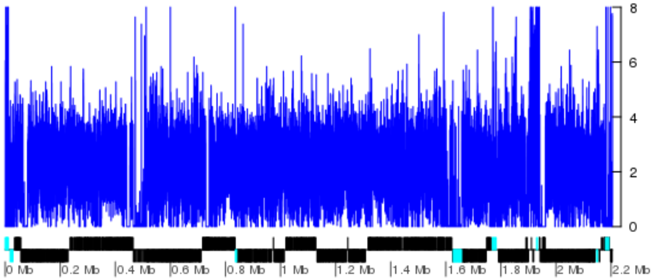

STIB704C-Tb927.08_v5.1

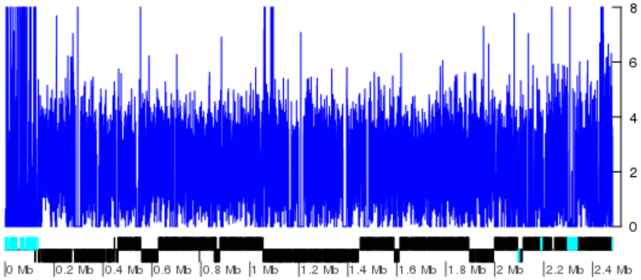

STIB704C-Tb927.09_v5.1

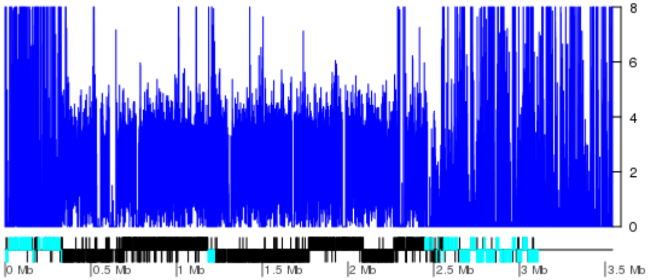

STIB704C-Tb927.10_v5.1

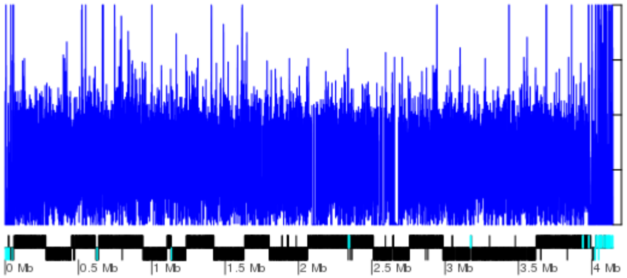

STIB704C-Tb927.11_v5.1

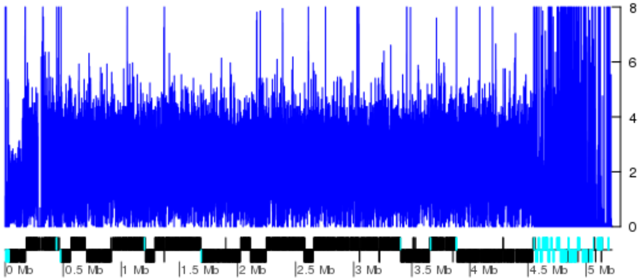

Isolate
STIB900

STIB900-Tb927.01_v5.1

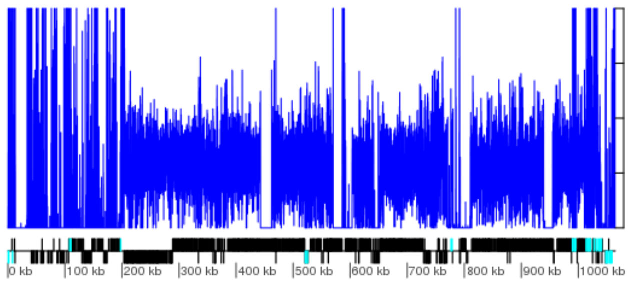

STIB900-Tb927.02_v5.1

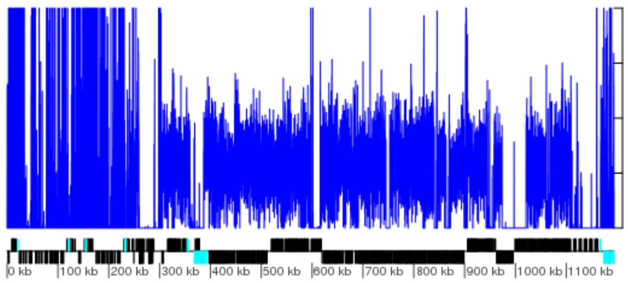

STIB900-Tb927.03_v5.1

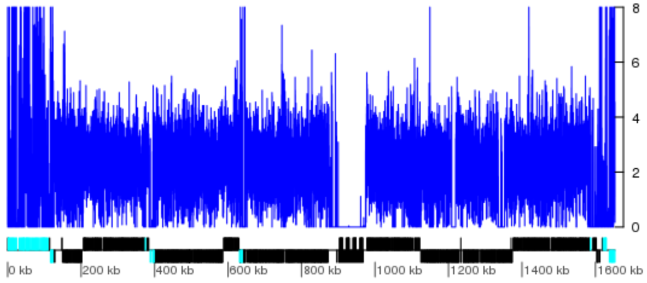

STIB900-Tb927.04_v5.1

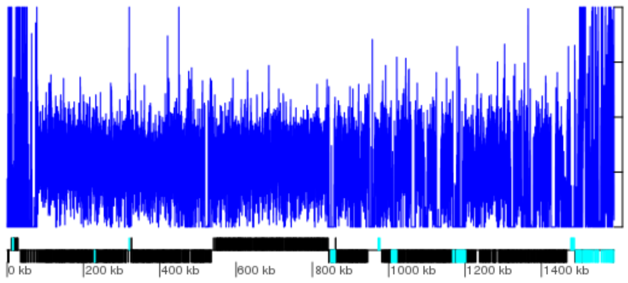

STIB900-Tb927.05_v5.1

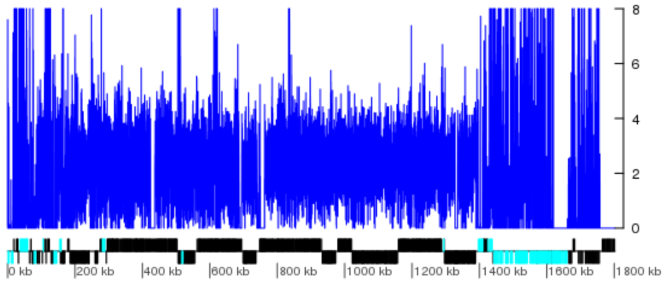

STIB900-Tb927.06_v5.1

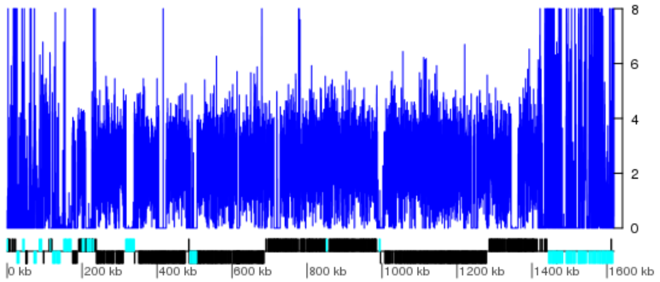

STIB900-Tb927.07_v5.1

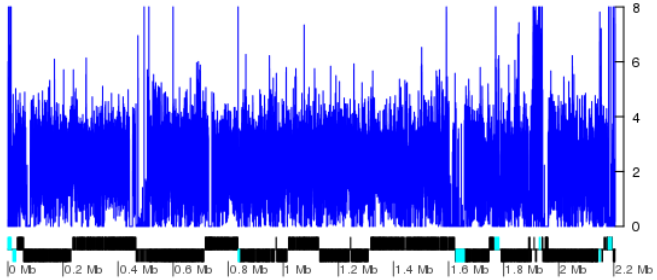

STIB900-Tb927.08_v5.1

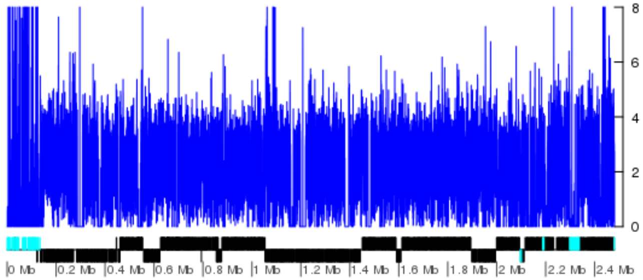

STIB900-Tb927.09_v5.1

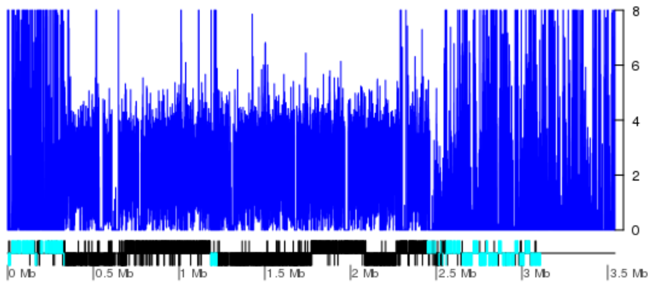

STIB900-Tb927.10_v5.1

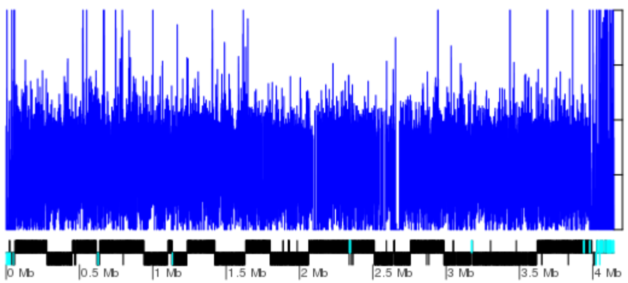

STIB900-Tb927.11_v5.1

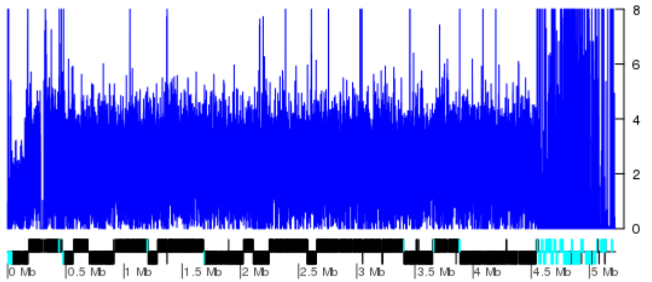

Isolate
STIB920

STIB920-Tb927.01_v5.1

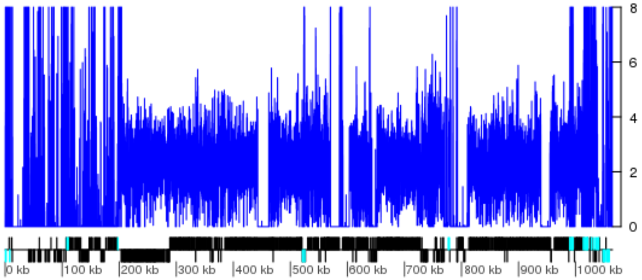

STIB920-Tb927.02_v5.1

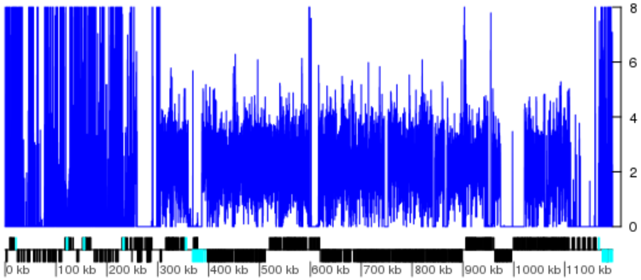

STIB920-Tb927.03_v5.1

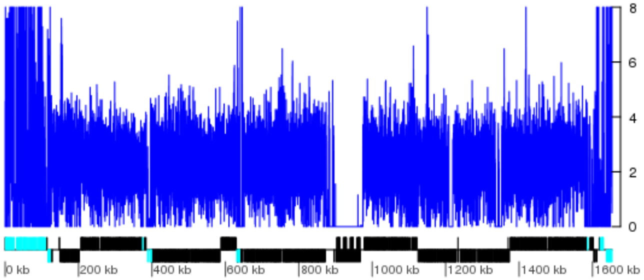

STIB920-Tb927.04_v5.1

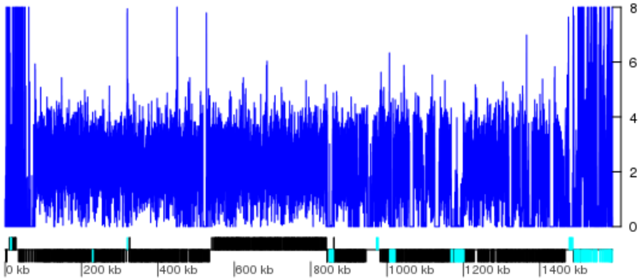

STIB920-Tb927.05_v5.1

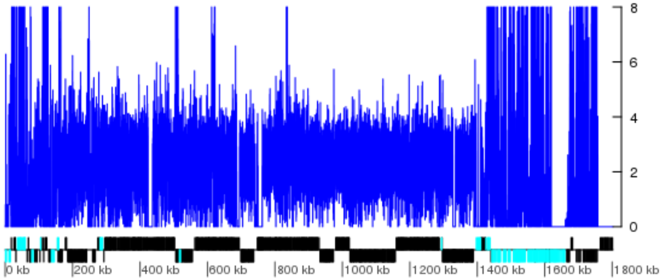

STIB920-Tb927.06_v5.1

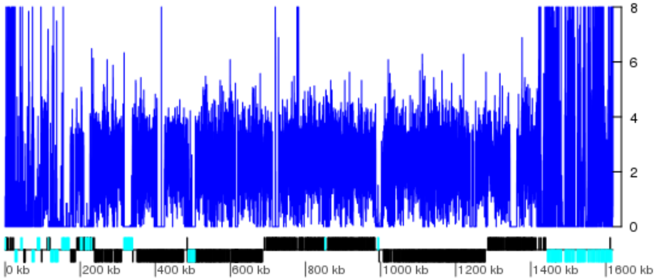

STIB920-Tb927.07_v5.1

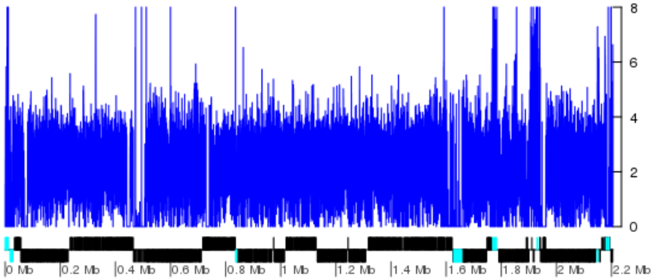

STIB920-Tb927.08_v5.1

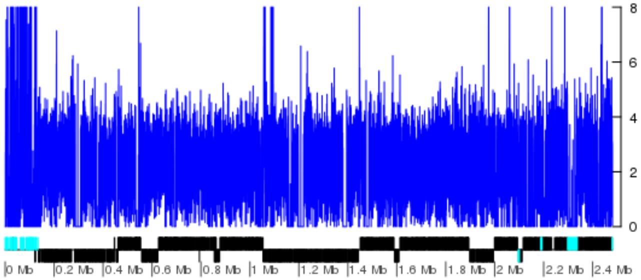

STIB920-Tb927.09_v5.1

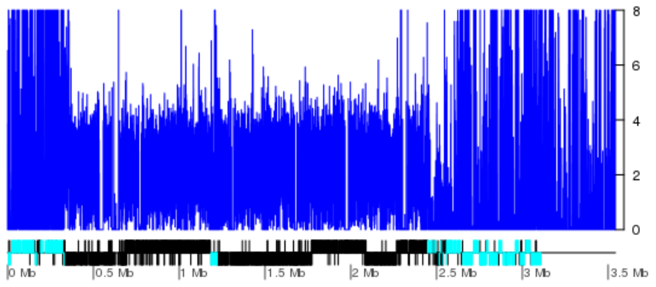

STIB920-Tb927.10_v5.1

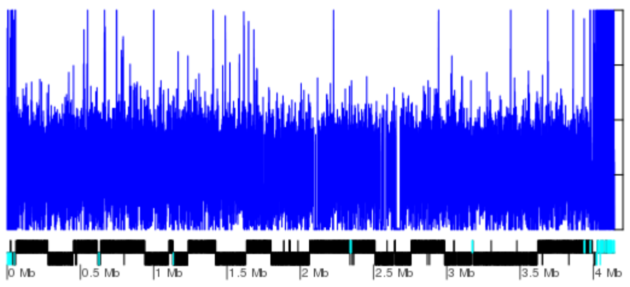

STIB920-Tb927.11_v5.1

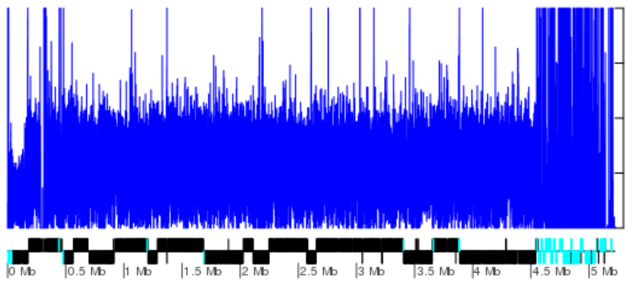

Isolate
ytat

ytat-Tb927.01_v5.1

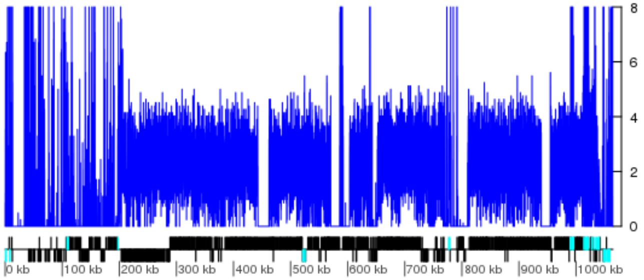

ytat-Tb927.02_v5.1

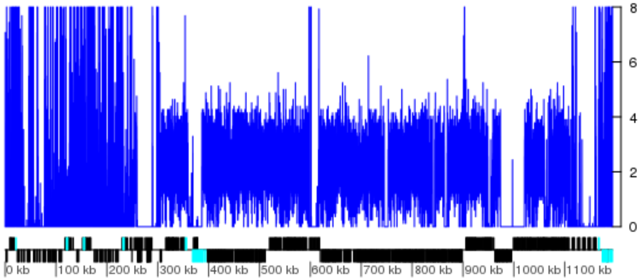

ytat-Tb927.03_v5.1

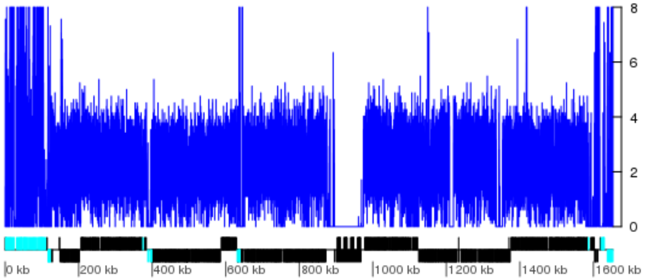

ytat-Tb927.04_v5.1

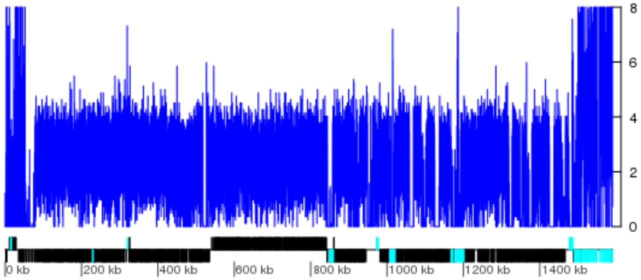

ytat-Tb927.05_v5.1

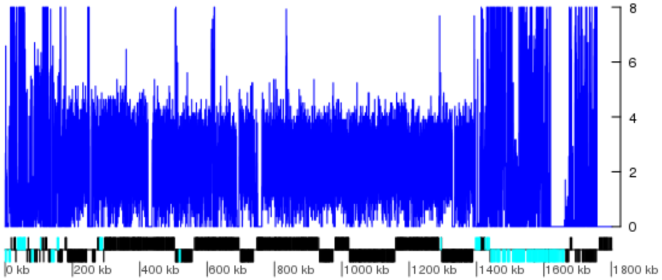

ytat-Tb927.06_v5.1

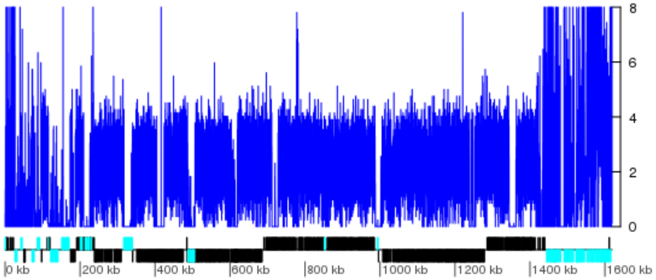

ytat-Tb927.07_v5.1

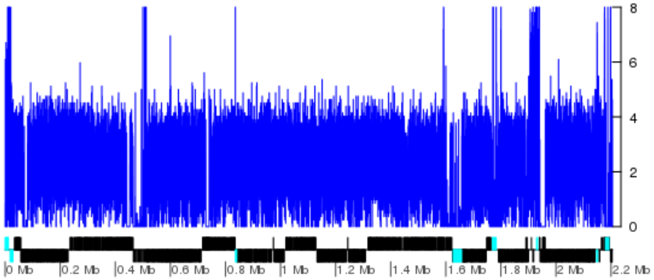

ytat-Tb927.08_v5.1

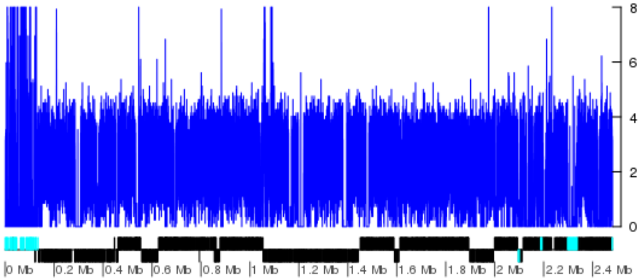

ytat-Tb927.09_v5.1

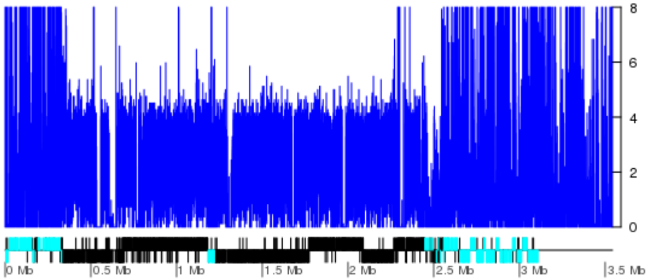

ytat-Tb927.10_v5.1

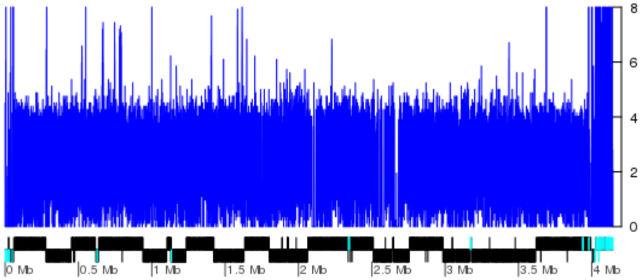

ytat-Tb927.11_v5.1

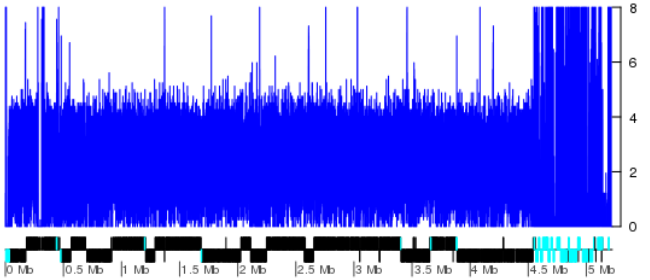

Supplement: Supplementary File 1 [file mgen-4-223-s001.pdf]
